# Identification, evolution, expression, and docking studies of fatty acid desaturase genes in wheat (*Triticum aestivum* L.)

**DOI:** 10.1186/s12864-020-07199-1

**Published:** 2020-11-10

**Authors:** Zahra Hajiahmadi, Amin Abedi, Hui Wei, Weibo Sun, Honghua Ruan, Qiang Zhuge, Ali Movahedi

**Affiliations:** 1 Department of Agricultural Biotechnology, Faculty of Agricultural Sciences, University of Guilan, Rasht, 4199613776 Iran; 2 Co-Innovation Center for Sustainable Forestry in Southern China, Key Laboratory of Forest Genetics & Biotechnology, Ministry of Education, Nanjing Forestry University, Nanjing, 210037 China

**Keywords:** Expression pattern, Gene family, Molecular docking, Protein structure

## Abstract

**Backgrounds:**

Fatty acid desaturases (FADs) introduce a double bond into the fatty acids acyl chain resulting in unsaturated fatty acids that have essential roles in plant development and response to biotic and abiotic stresses. Wheat germ oil, one of the important by-products of wheat, can be a good alternative for edible oils with clinical advantages due to the high amount of unsaturated fatty acids. Therefore, we performed a genome-wide analysis of the wheat *FAD* gene family (*TaFADs*).

**Results:**

68 *FAD* genes were identified from the wheat genome. Based on the phylogenetic analysis, wheat *FADs* clustered into five subfamilies, including *FAB2*, *FAD2/FAD6*, *FAD4*, *DES/SLD*, and *FAD3/FAD7/FAD8*. The *TaFADs* were distributed on chromosomes 2A-7B with 0 to 10 introns. The Ka/Ks ratio was less than one for most of the duplicated pair genes revealed that the function of the genes had been maintained during the evolution. Several cis-acting elements related to hormones and stresses in the *TaFAD*s promoters indicated the role of these genes in plant development and responses to environmental stresses. Likewise, 72 SSRs and 91 miRNAs in 36 and 47 *TaFAD*s have been identified. According to RNA-seq data analysis, the highest expression in all developmental stages and tissues was related to *TaFAB2.*5, *TaFAB2.12*, *TaFAB2.15*, *TaFAB2.17*, *TaFAB2.20*, *TaFAD2.1*, *TaFAD2.6*, and *TaFAD2.8* genes while the highest expression in response to temperature stress was related to *TaFAD2.6*, *TaFAD2.8*, *TaFAB2.15*, *TaFAB2.17*, and *TaFAB2.20*. Furthermore, docking simulations revealed several residues in the active site of TaFAD2.6 and TaFAD2.8 in close contact with the docked oleic acid that could be useful in future site-directed mutagenesis studies to increase the catalytic efficiency of them and subsequently improve agronomic quality and tolerance of wheat against environmental stresses.

**Conclusions:**

This study provides comprehensive information that can lead to the detection of candidate genes for wheat genetic modification.

**Supplementary Information:**

The online version contains supplementary material available at 10.1186/s12864-020-07199-1.

## Background

Polyunsaturated fatty acids (PUFAs) are essential components of the plasma membrane. Various PUFAs have crucial roles in plant physiological and cellular processes such as cold acclimation, defense mechanisms against biotic and abiotic stresses, and chloroplast development [1]. PUFAs biosynthesis occurs through different and complex pathways of desaturation and elongation steps [2]. Fatty acid desaturase (FAD) enzymes introduce double band into fatty acids hydrocarbon chain. Two groups of FAD have been identified in plants, including acyl–acyl carrier protein (acyl-ACP) desaturases and membrane-bound FADs or acyl-lipid desaturases [3]. While identified FADs in plants, animals, algae, and fungi are membrane-bound desaturase, the plant acyl-ACP desaturase (FAB2/SAD) is the only soluble FAD [4, 5]. The acyl-ACP desaturases introduce the first double band into the acyl chain of saturated fatty acid in plastids. Besides, Membrane-bound FADs exist in chloroplast and endoplasmic reticulum (ER). Desaturation processes occur through two different pathways in the chloroplast and the ER [6]. In the chloroplast and ER, double bond formation requires NADPH/ferredoxin and NADH/cytochrome b_5_ systems as the electron donors, respectively [7].

On the other hand, the quality of edible oils depends on the unsaturated fatty acids content [8]. FADs are essential to determine the quality of edible oils [9]. They have been attracted more attention due to their ability to adjust the level of unsaturated fatty acids to increase the quality of these oils and plant resistance against various stresses including drought, salt, heat, cold, and pathogen [10–13]. For instance, the cell membrane is the primary site for cold-induced injury, and the melting temperature of the unsaturated fatty acids is less than saturated fatty acids. Therefore, adjustment of membrane lipid fluidity through manipulation of FADs and changing the levels of unsaturated fatty acids might seem helpful for cold acclimation [14]. To date, several studies have been conducted to assess the expression of genes encoding fatty acid desaturase in response to biotic and abiotic stresses [12, 15–17]. Investigation of the expression of *SACPD-A* and *SACPD-B* genes (encoding soluble Δ9 stearoyl-ACP desaturases) and the amount of stearic acid (C18:0) and oleic acid (C18:1) in soybean revealed that the number of transcripts of both genes and oleic acid had been dramatically increased in low temperature. Reversely, we observed an increased amount of C18:0 and decreased the expression of the genes above at high temperatures [18]. Wang et al. (2012) ascertained the expression of oleate desaturase (*GbFAD2* and *GbFAD6*) and *GbSAD* genes under various temperatures in *Ginkgo biloba* L. leaves. Based on their results, the expression of *GbFAD2* and *GbSAD* genes has been increased in 4 and 15 °C, while it has been prevented in 35 and 45 °C.

In contrast, the expression of *GbFAD6* was constant at different temperatures [19]. The expression of *FAD2–1* and *FAD2–2* genes of olive has been increased in response to wounding [20]. Likewise, *FAD2* and *FAD6* genes are necessary for salt tolerance during early seedling in Arabidopsis [21, 22]. Zhang et al. (2005) developed transgenic tobacco plants with the overexpressing *FAD3* or *FAD8* genes. According to their findings, the over-expression of *FAD8* or *FAD3* genes caused enhanced tolerance to drought [23]. The importance of FADs in plant pathways has been confirmed previously. A homologous region based on a conserved sequence of a gene family can be applied to identify new genes. The *FAD* gene family is vital for the production of PUFAs in plants; thus, a comprehensive understanding of *FAD* genes using bioinformatics studies can help disclose their functions in the studied plants.

**Fig. 1:**
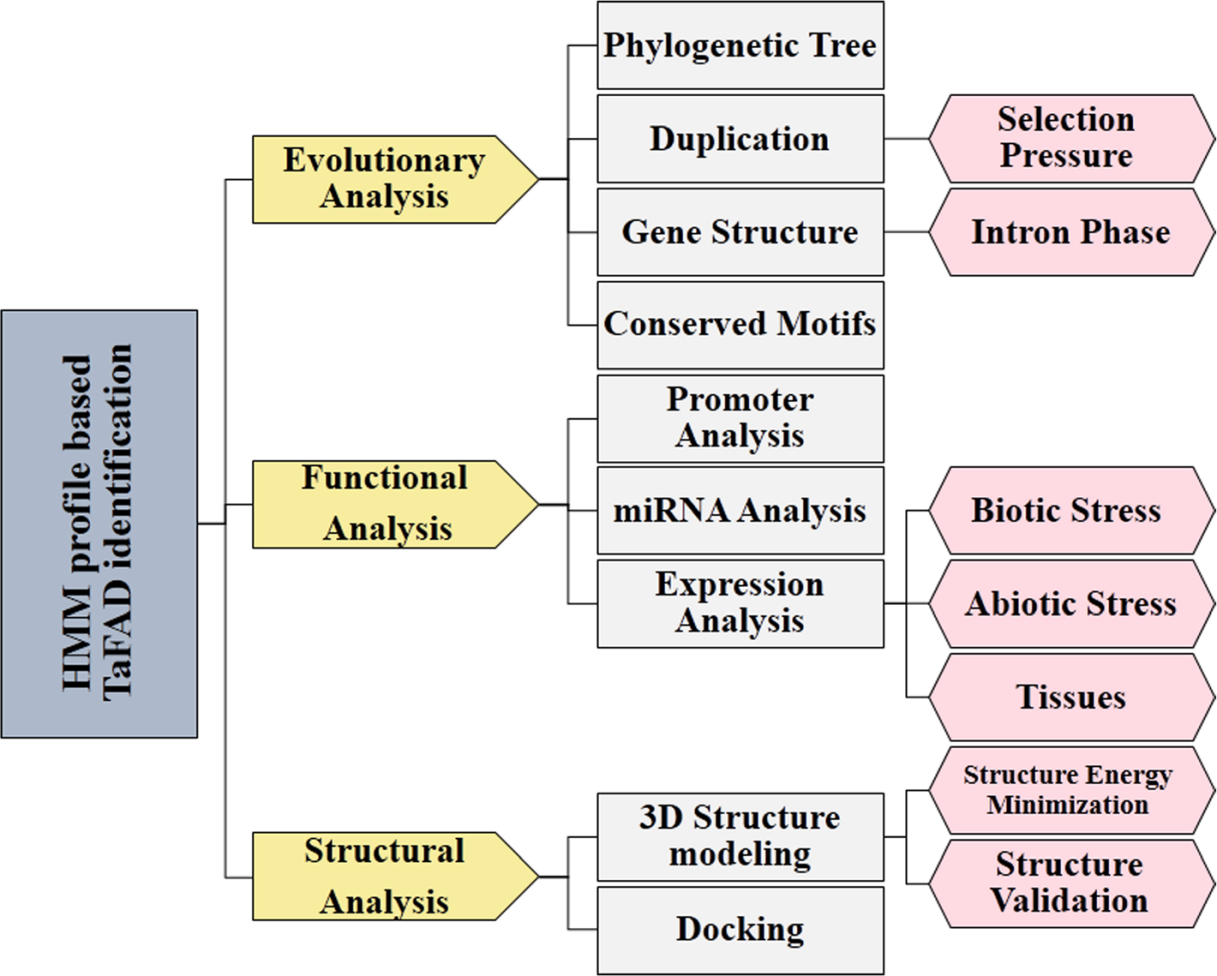
A flow-chart of the data analysis process

Wheat (*Triticum aestivum* L.) is one of the most important cereal crops. Because of the high amount of unsaturated fatty acids, wheat germ oil, one of the essential by-products of wheat, can be a good alternative for edible oils with clinical benefits. Based on studies, wheat germ oil contain different fatty acids, including linoleic acid (C 18:2), palmitic acid (C 16:0), oleic acid (C 18:1), linolenic acid (C18:3), and stearic acid (C 18:0) [24]. Wheat is a good source of edible oil, and the characterization and analysis of the *FAD* family in wheat plants have not yet been performed. On the other hand, comprehensive analyses on gene families help to address a better understanding of their evolutions and functions in plants [25]. Therefore, in this study, identification, evolutionary relationship, duplication and selection pressure, exon-intron structure, promoter analysis, transcript-targeted miRNA and simple sequence repeat markers prediction, RNA-seq data analysis, three-dimensional structure, and docking studies of the *TaFADs* have been investigated in wheat using bioinformatics tools. Figure 1 provides a flow-chart of the data analysis process.

## Results

### Identification of *T. aestivum FAD* genes

In the current study, 82 TaFAD (coding by 68 genes) have been identified. All identified *FAD* genes were named based on a phylogenetic dendrogram, and their numbering was according to the location of genes on chromosomes. The physicochemical study of the identified genes was carried out using the ProtParam tool. Based on the results, these genes are different in the number of amino acids, molecular weight (MW), and isoelectric point (pI). The protein sequence encoded by these 68 *FAD* genes ranged in length from 281 amino acids of TaFAD4.2 to 518 amino acids of TaFAD6.2. The theoretical molecular weights of these proteins ranged from 29.74 to 59.63 kDa, with the isoelectric points varied from 5.42 to 9.72 (Additional file 1: Table S1). The results of cellular localization of proteins revealed that they are active in chloroplast, mitochondria, endoplasmic reticulum, and plasma membrane. The spatial diversity of these genes is likely related to the diverse functional roles of these genes in different cell processes.

### Phylogenetic analysis of the wheat *FAD* gene family

**Fig. 2:**
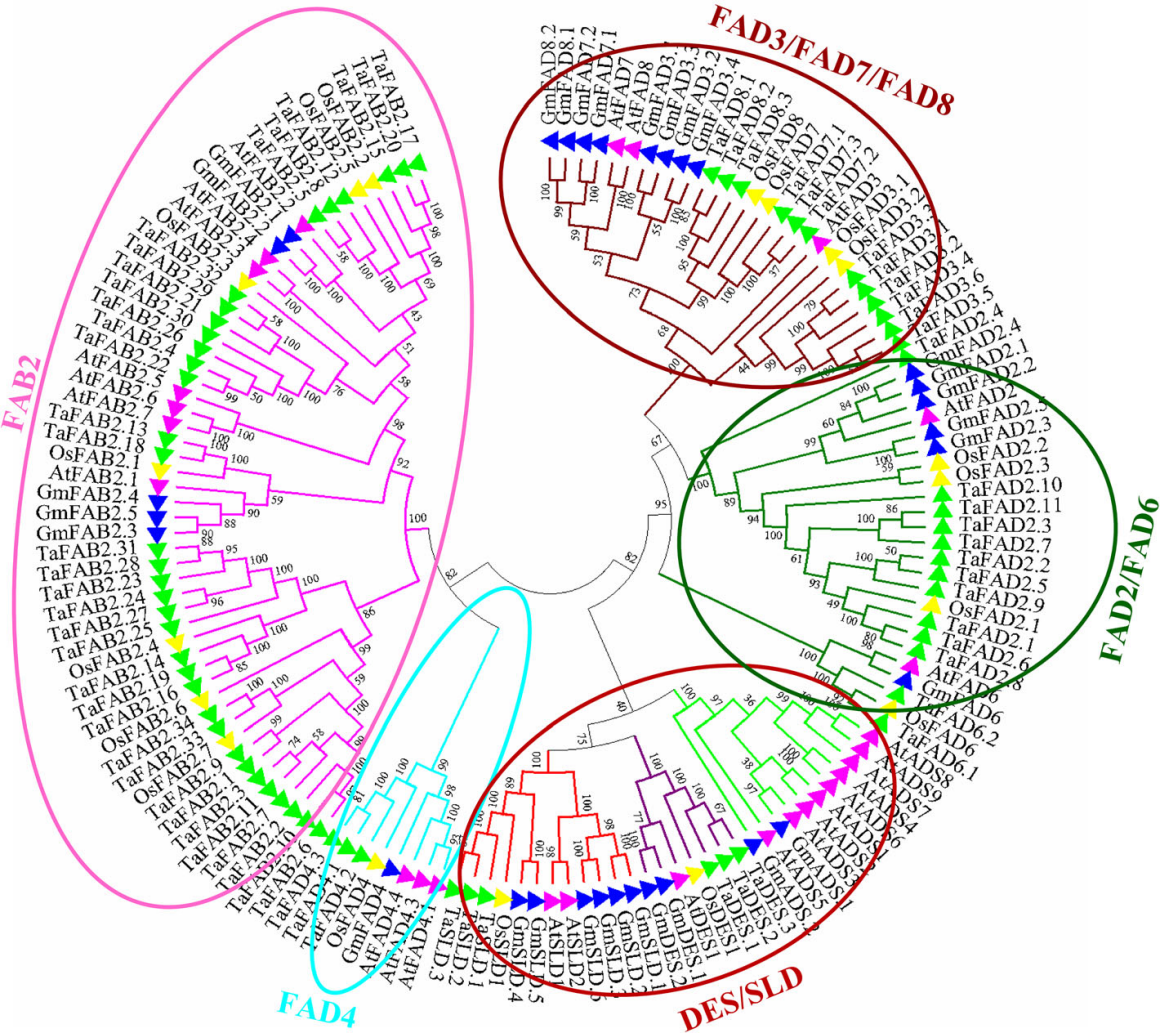
Phylogenetic relationships of *FAD* genes from wheat, Arabidopsis, rice, and soybean. The colored branch shows a different subfamily. The tree was constructed using MEGA 7 by the neighbor-joining (NJ) method with 1000 bootstraps

We carried out a phylogenetic tree using the Neighbor-joining method to investigate relationships of wheat FAD proteins. According to Fig. 2, wheat FAD proteins were divided into five groups. The first group is FAB2, which is called stearoyl-ACP desaturase (SADs) in plants. It can introduce a double bond into the stearoyl-ACP at the delta-9 position. Based on the phylogenetic tree, the *FAB2* subfamily has been noticeably separated from other subfamilies. All members of wheat and rice FAB2 were clustered together, whereas FAB2 of Arabidopsis and soybean (as dicot plants) were grouped. FAD4 group introduces a double bond into a saturated acyl chain at the delta-3 position. Likewise, all FAD4 proteins of monocot plants were clustered together and separated from dicot plants clade. In *FAD2/FAD6* (Delta-12 desaturase, omega 6) subfamily, FAD2 and FAD6 were divided into two separate clades due to their subcellular localization (Additional file 1: Table S1). FAD2 is endoplasmic reticulum-type omega 6, while FAD6 is chloroplast-type omega 6. In *FAD3/FAD7/FAD8* (Delta-15 desaturase, omega 3) subfamily, the FAD7 clade is closer to the FAD8 clade in wheat, which may be due to the high similarity of their sequences. The subcellular localization of FAD7 and FAD8 is the chloroplast, while the FAD3 is endoplasmic reticulum-type omega 3.

### Gene location on a chromosome, gene duplication, and selection pressure of *FAD* genes

**Fig. 3:**
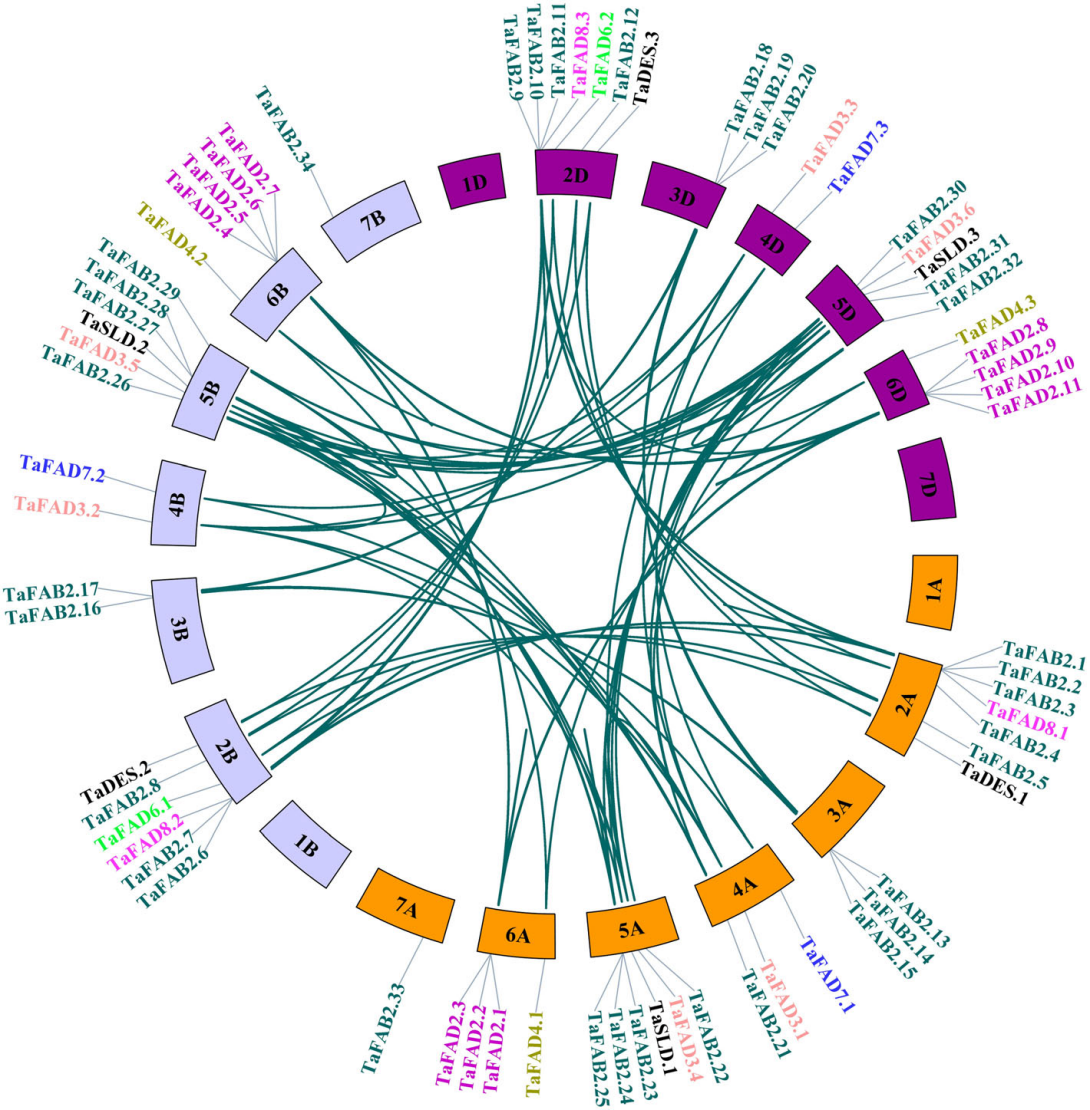
Chromosomal locations of wheat fatty acid desaturase genes. Chromosomes are represented by Colored boxes. Dark teal curves connecting the genes indicate duplications. The location of genes on chromosomes and the duplication relationship between them were presented using TBtools

To assess the distribution of *FAD* family genes, a chromosome map has been constructed, and an uneven distribution of 68 *FAD* genes on wheat chromosomes was determined (Fig. 3). Chromosomes (Chr) 2A, 2D, 2B, 5A, and 5B had the highest number of genes while there was no gene of this family on chr1A-1D and 7D. Only one gene has been found on chr7A and chr7B related to *TaFAB2.33* and *TaFAB2.34*, respectively. Different members of the *FAB2* subfamily are located on chr2A-7B except for ch4B and ch4D. In *SLD* and *DES* subfamilies, the genes were determined on chr5A-5D and chr2A-2D, respectively. *FAD3*, *FAD6*, *FAD7*, and *FAD8* subfamilies have been found on chr4A-4D, chr4A-5D, chr2A-2D, and chr2B-2D, respectively.

Two types of tandem and segmental duplication were observed in the wheat *FAD* gene family. However, according to the higher frequency of segmental duplication, its role in the expansion of the *FAD* gene family is much greater than tandem duplication. Gene duplication is one of the important mechanisms to increase genetic diversity and generate new genes in plants. In the current study, it was found that three genes on chr6A (*TaFAD2.1–3*), four genes on chr6B (*TaFAD2.4–7*), and four genes on chr6D (*TaFAD2.8–11*) are the result of tandem duplication. Likewise, threes genes on chr2A (*TaFAB2.1–3*), chr2D (*TaFAB2.9–11*), chr5B (*TaFAB2.26–28*), and chr5A (*TaFAB2.23–25*) are the result of tandem duplication. We investigated Ka, Ks, and Ka/Ks parameters for 153 paired genes (Additional file 1: Table S2) to reveal a functional selection pressure between duplicated genes. In general, Ka/Ks > 1, Ka/Ks = 1, and Ka/Ks < 1 indicate positive, neutral, and negative selections, respectively [26]. The Ka/Ks ratio was less than one for most of the paired genes. This negative selection is to maintain the function of the *FAD* gene family in wheat plants, and they were under a slow evolutionary process, and almost their role in evolution has been maintained. However, the Ka/Ks ratio for seven paired genes (*TaFAD2.4/TaFAD2.7*, *TaFAD2.6/TaFAD2.5*, *TaFAD2.2/TaFAD2.6*, *TaFADB2.1/TaFADB2.2, TaFADB2.9/TaFADB2.10*, *TaFADB2.15/TaFADB2.20,* and *TaFADB2.1/TaFADB2.11*) was greater than one, indicated the aforementioned paired genes were under the positive selection during evolution and they have different functions due to the mutations that have been occurred during their evolution. The divergence time of duplications was estimated at 1.98–47.75 Myra.

### Exon-intron structures and conserved motifs

**Fig. 4:**
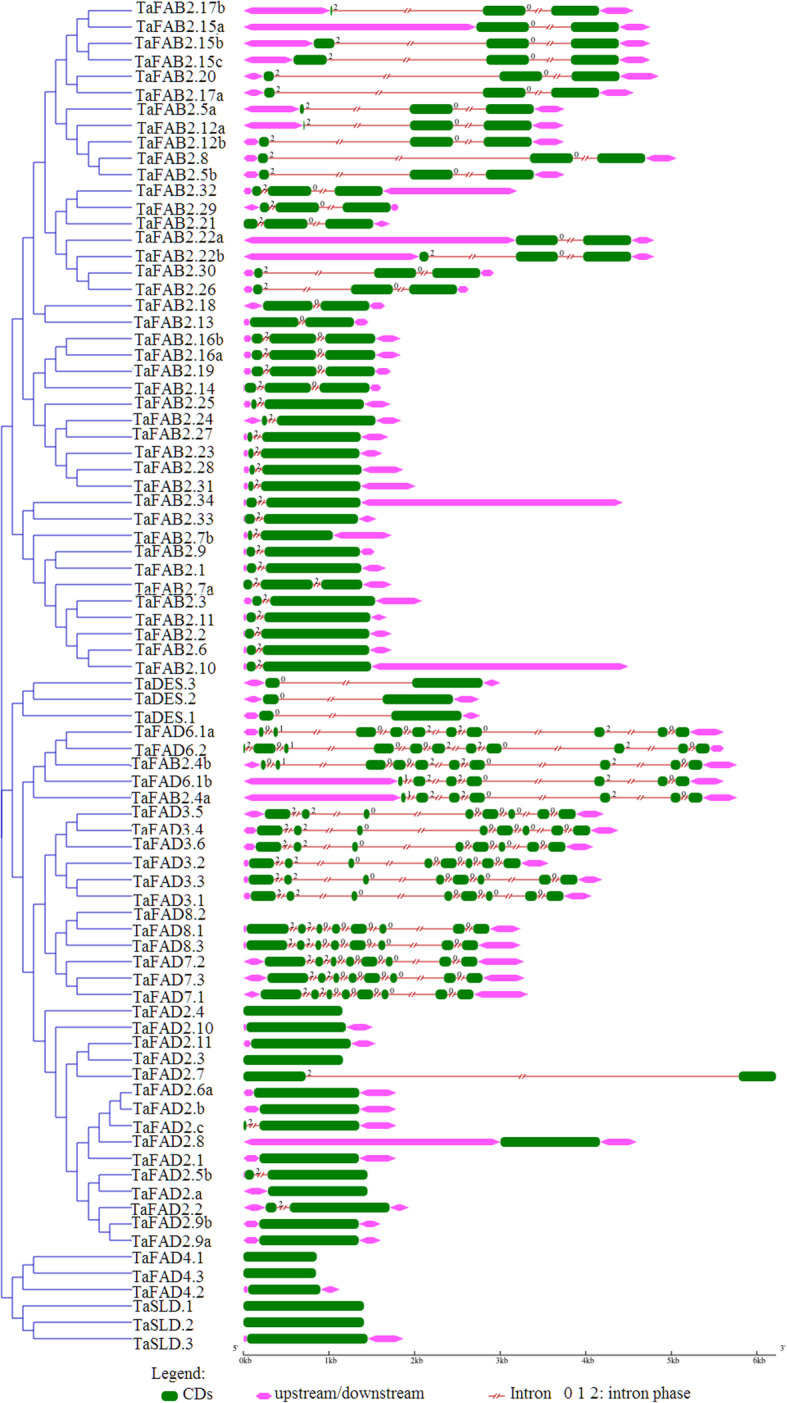
The exon-intron structure of *FAD* genes in wheat. Exons and introns were represented by green boxes and red lines, respectively. The exon-intron structure of the *BnATGs* was determined using a gene structure display server (GSDS)

Based on the analysis of the exon-intron structure of the *FAD* gene family, they had 0 to 10 introns with high structural diversity (Fig. 4). 21.95% of wheat *FAD* genes are intronless. The longest intron is related to the *TaFAD2.7* gene. Three intron splicing phases have been observed for the wheat *FAD* gene family, including phase zero, splicing after the third nucleotide of the codon; phase one, splicing after the first nucleotide of the codon; and phase two, splicing after the second nucleotide [27]. Most of the genes in this family have phases zero and two, whereas *TaFAD6.1–2* and *TaFAB2.4* genes have all three intron splicing phases. In *TaFAB2.13*, *TaFAB2.18*, *TaFAB2.22a*, *TaFAB2.15*, and *TaDES.1–3* genes only phase zero have been observed. Likewise, *TaFAB2.1–3*, *TaFAB2.23–25*, *TaFAB2.27–28*, *TaFAB2.6–7*, *TaFAB2.9–11*, *TaFAB2.33–34*, and *TaFAB2.31* genes demonstrated only phase two. The genes of the same subfamily were more similar in exon-intron structure compared with the genes of the other subfamilies. The *FAD3/7/8* and *DES* subfamilies have eight and two exons, respectively. However, the *FAB2* subfamily has 2–3 exons except for *TaFAB2.4b* with 10 exons. The *SLD*, *FAD2*, and *FAD4* subfamilies have one exon except for *TaFAD2.2*, *TaFAD2.5b*, *TaFAD2.6c*, and *TaFAD2.7* with two exons. The *FAD6* subfamily contains 10 exons, while *TaFAD6.1b* has seven exons. A comparison of gene structure and phylogenetic tree demonstrated that the gene structure and intron splicing phases were similar in each gene cluster except *TaFAB2.4.* (Fig. 4).

**Fig. 5:**
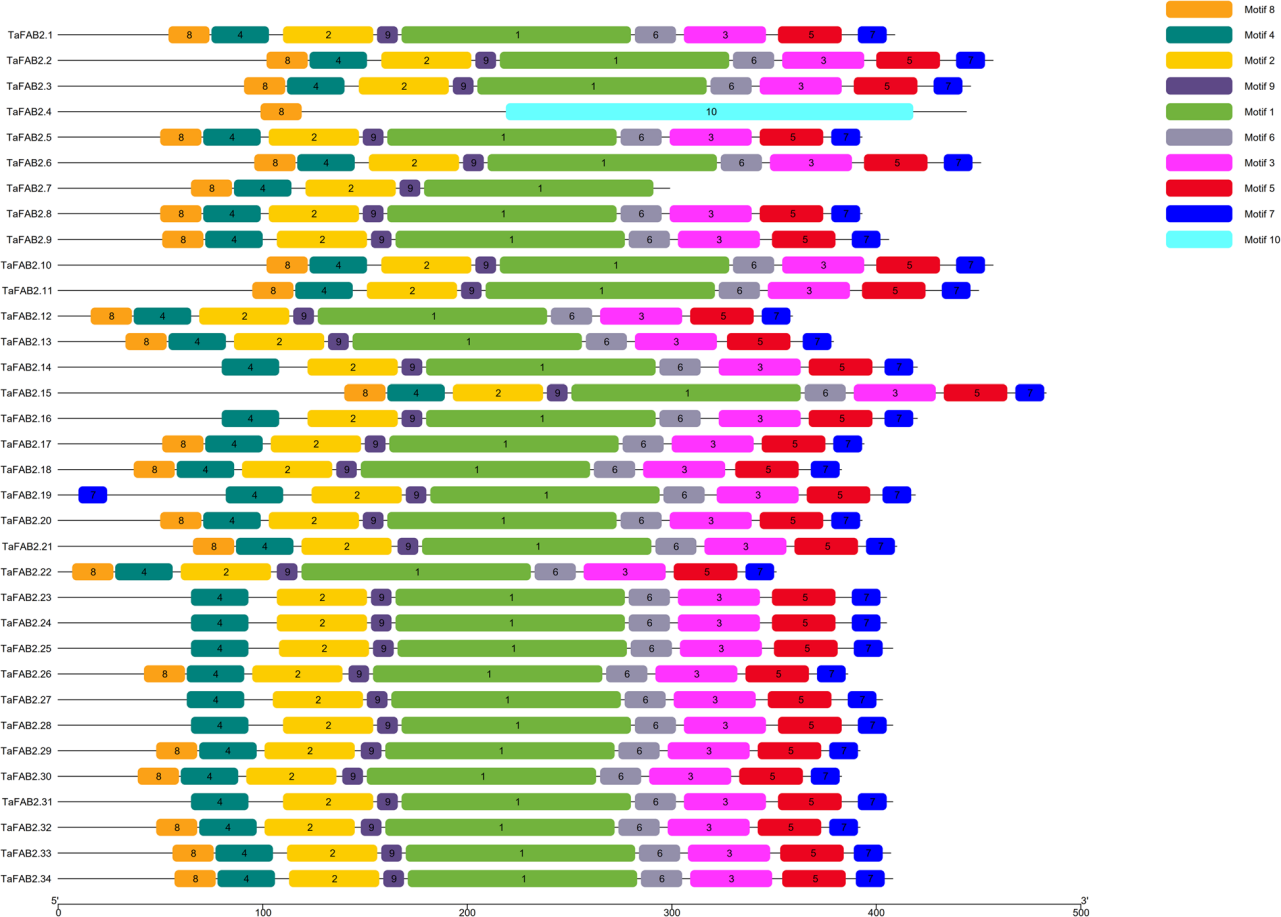
The conserved motifs of the *TaFAB2* subfamily. Different motifs are presented in different colors. Motifs were detected using the MEME online tool

**Fig. 6:**
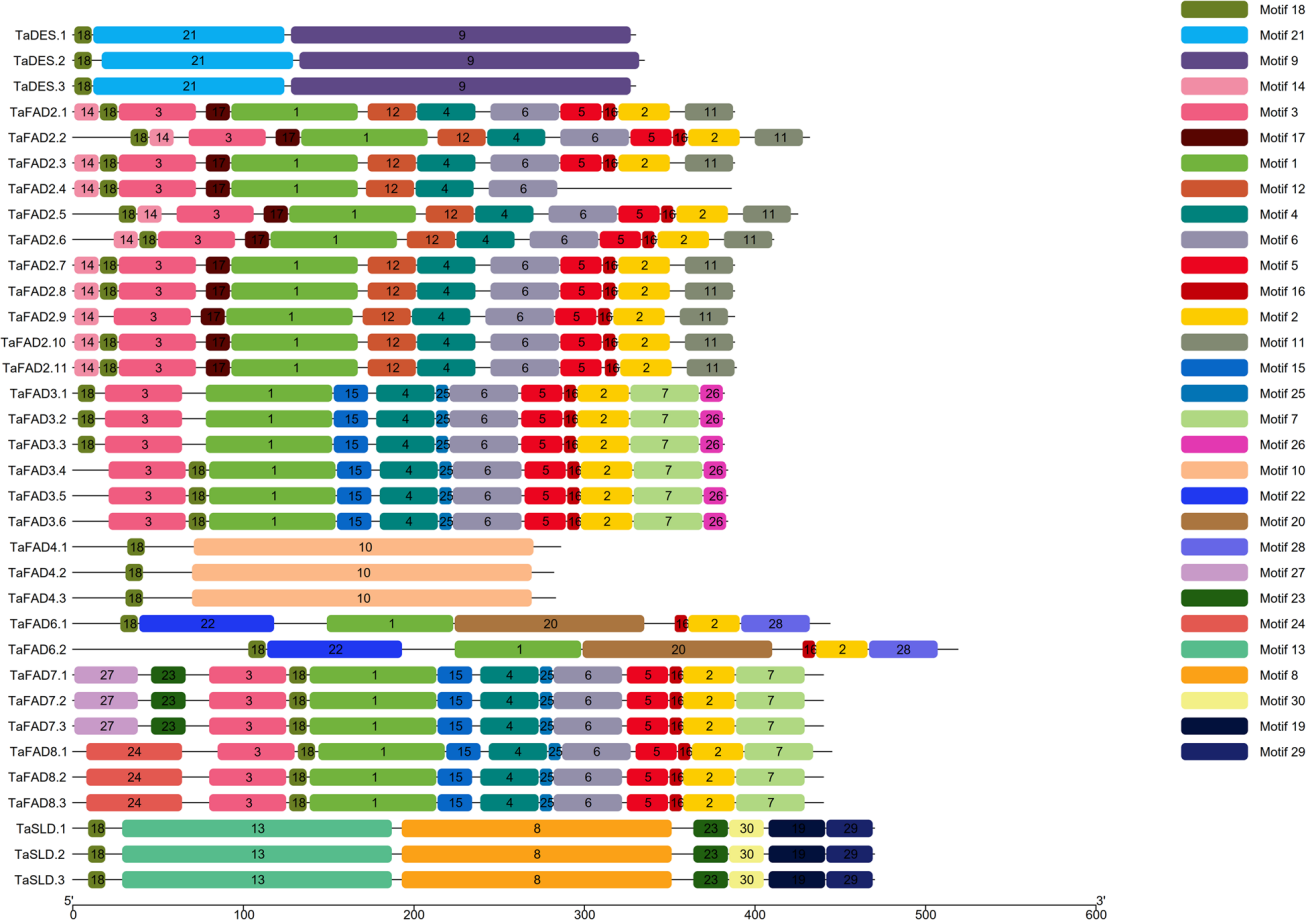
The conserved motifs of the *TaFAD* subfamily. Different motifs are presented in different colors. Motifs were detected using the MEME online tool

MEME tool has been applied to detect conserved motifs in protein sequences of the *FAD* family (Additional file 1: Table S3 and S4). Based on the results, 30 and 10 conserved motifs in the *FAD* and *FAB2* subfamilies have been identified, respectively (Figs. 5 and 6). Evaluation of motifs with Pfam showed that motifs 1, 2, 8, 9, and 10 in the *FAD* subfamily are related to the *FAD* gene family. The motifs 1–5 in the *FAB2* subfamily are also related to the fatty acid desaturases. Conserved motif 13 (Cytochrome b5-like Heme/Steroid binding domain) was presented only in the TaSLD1–3 (Additional file 1: Table S4). Motif 18 (AAAARADSGEA) with no function was found in all the members of the *FAD* subfamily. The lowest number of motifs was related to TaFAD4.1–3 with two motifs (Motifs 18 and 10). Motifs 1, 2, 4, and 9 were common in all the members of the *FAB2* subfamily except TaFAB2.4. TaFAB2.4 has only motifs 8 and 10 with no function. All members of the *FAD* subfamily contain three conserved histidine boxes (Additional file 1: Table S5). The amino acid composition of His-boxes is highly conserved in the same subfamilies. The third His-box with consensus sequence HX_2_HH exists in all TaFAD except in the TaSLD1–3 that the first amino acid is glutamine instead of histidine, which is vital for its enzymatic activity. All members of the *FAB2* subfamily contain two conserved histidine boxes. The first His-box with consensus sequence EENRHG exists in all TaFAB2 except TaFAB2.8 with the consensus sequence of EENRML, whereas the sequence of the second His-box (DEKRHE) is identical in all *FAB2* subfamily members.

### Detection of cis-acting elements in TaFAD promoters

To better understanding the *TaFAD* genes expression regulation mechanisms, the search of cis-acting elements 1500 bp upstream of starting codon (ATG) of *TaFAD* genes was performed using the PlantCare database. In total, 82 motifs were identified in this family that the abundance of these elements was highly varied for each gene (Additional file 1: Table S6). The highest frequency of motifs was related to STRE (95.58%), MYC (94.11%), MYB (89.70%), TGACG-motif (88.23%), CGTCA-motif (88.23%), as-1 (88.23%), ABRE (85.29%), and G-box (83.82%). Likewise, the lowest frequency of motifs was related to BoxIII (only in *TaFAB2.21*), AT1-motif (only in *TaFAD2.11*), xhs-MA1a (only in *TaFAB2.24*), L-box (only in *TaFAD3.2*), LAMP-element (only in *TaFAD8.1*), AAAC-motif (only in *TaFAB2.10*), F-box (only in *TaFAB2.3*), Plant_AP_2_like (only in *TaFAB2.15*), re2f-1 (only in *TaFAB2.23*), and OCT (only in *TaFAB2.28*).

### Simple sequence repeats (SSRs) in TaFAD genes and TaFAD-targeted miRNAs prediction

72 SSRs have been identified in 36 of the 68 *TaFAD* genes including 33, 25, 11, 2, and 1 SSRS in *FAD3/FAD7/FAD8*, *FAB2*, *FAD2/FAD6*, *DES/SLD*, and *FAD4* subfamilies, respectively (Additional file 1: Table S7). Most genes had a single SSR except *FAB2.22* (5SSRs), *FAD8.1* (5 SSRs), *FAD8.3* (5 SSRs), *FAD2.7* (5 SSRs), *FAD3.1* (4 SSRs), *FAD3.3* (4 SSRs), *FAD3.2* (3 SSRs), *FAD3.4* (3 SSRs), *FAD3.6* (2 SSRs), *FAD8.2* (3 SSRs), *FAB2.15* (3 SSRs), *FAB2.20* (2 SSRs), *FAB2.34* (3 SSRs), *FAB2.31* (2 SSRs), and *FAD2.8* (2 SSRs). The highest frequency was related to tri-nucleotide repeats (36 SSRs) followed by tetra-nucleotide repeats (6 SSRs), penta-nucleotide repeats (13 SSRs), hexa-nucleotide repeats (4 SSRs), and di-nucleotide repeats (3 SSRs). 91 miRNAs for 47 *TaFADs* possible targets have been identified (Additional file 1: Table S8). The relationship between miRNAs and their targets was not one by one and many miRNAs had a common target. For instance, *TaSLD3* transcript was co-targeted by 5 miRNAs named tae-miR9659-3p, tae-miR1137a, tae-miR395a, tae-miR395b, and tae-miR9677b. On the contrary, one miRNA had multiple transcript targets such as tae-miR5384-3p can suppress the expression of *TaFAB2.1*, *TaFAB2.9*, *TaFAB2.11*, *TaFAB2.21*, *TaFAB2.32*, *TaFAB2.33*, and *TaFAB2.34*.

### Expression analysis of *TaFAD* genes

**Fig. 7:**
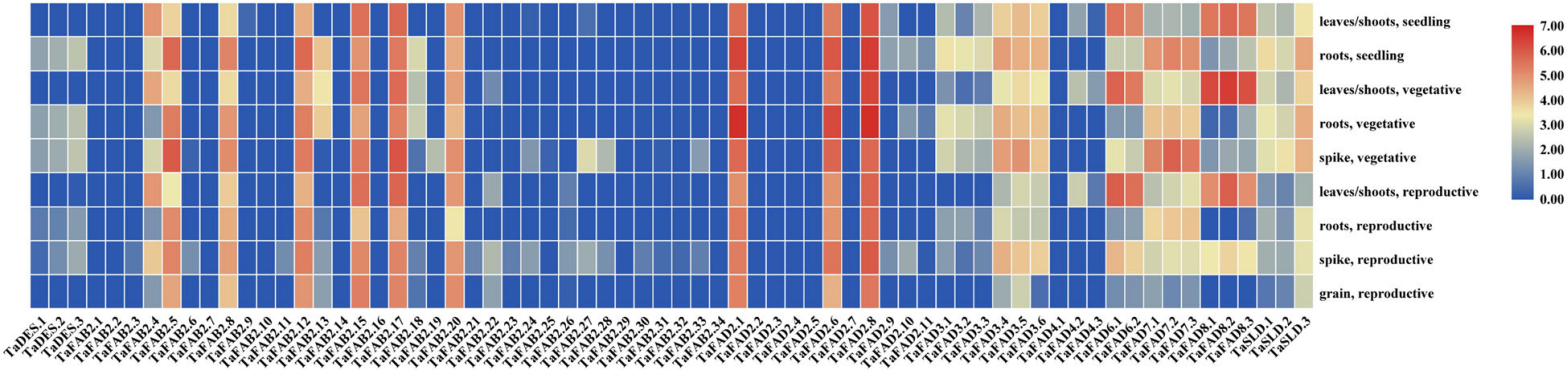
The expression pattern of *TaFAD* genes at different developmental stages and tissues. The color boxes indicate expression values, the lowest (green), medium (Pale goldenrod), and the highest (red). The heatmap was generated using log10 (TPM + 1) values using TBtools software

**Fig. 8:**
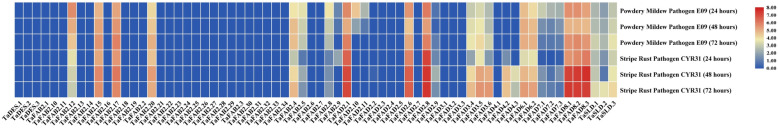
The expression pattern of *TaFAD* genes under biotic stress. The color boxes indicate expression values, the lowest (blue), medium (pale goldenrod), and the highest (red). The heatmap was generated using log10 (TPM + 1) values using TBtools software

**Fig. 9:**
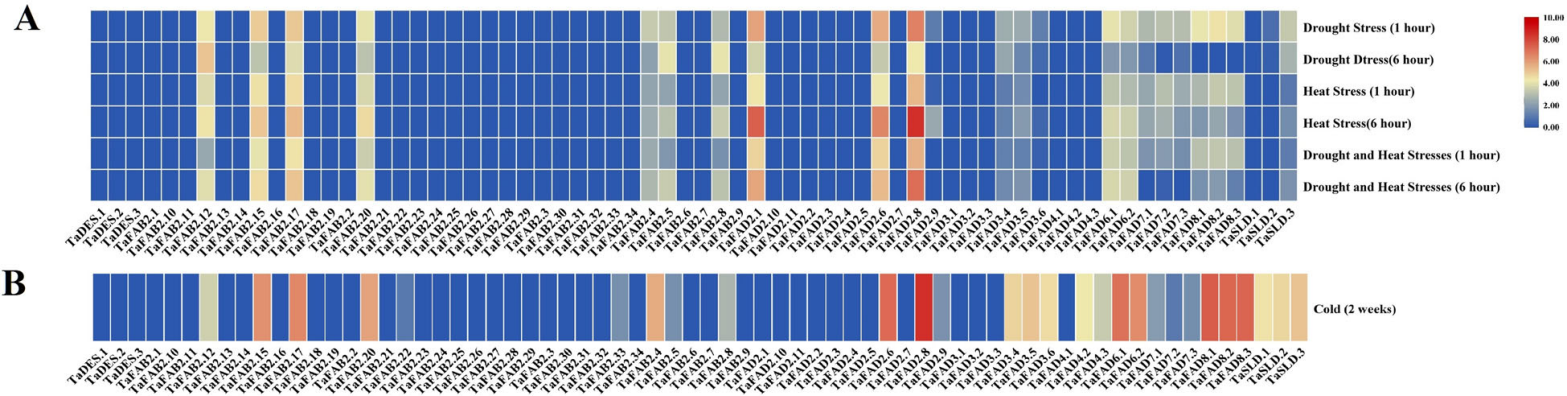
The expression pattern of *TaFAD* genes under heat, drought (**a**), and low temperature (**b**) conditions. The color boxes indicate expression values, the lowest (blue), medium (pale goldenrod), and the highest (red). The heatmap was generated using log10 (TPM + 1) values using TBtools software

To understand the functional role of *TaFAD* genes, their expression patterns of different developmental stages have been studied in the wheat root, shoot, leave, grain, and spike tissues (Fig. 7) (Additional file 1: Table S9). The members of the *TaFAD* family showed different expression patterns. All members of *DES/SLD* subfamily revealed low transcript level at all developmental stages and tissues except *TaSLD3* (reduced expression in leaves and shoots of seedling and growth cycles, high expression at the vegetative stage in roots and spikes), *TaSLD1* (moderate expression at seedling stage in roots), and *TaSLD2* (reduced expression at the vegetative stage in spikes). In the *FAD2/FAD6* subfamily, all *TaFAD2* genes had low expression except *TaFAD2.1*, *TaFAD2.6*, and *TaFAD2.8* with a high level of transcripts in all stages and tissues. However, *TaFAD6.1–2* genes had a high level of expression in shoots, leaves at the all studied developmental stages, and spike at the reproductive phase. Based on RNA-seq data analysis of the *TaFAD4* subfamily, all members demonstrated a low level of transcripts in all stages and tissues. In the *FAD3/FAD7/FAD8* subfamily, the low level of transcript expression of *TaFAD7.1–3* genes has been observed in vegetative, reproductive, and seedling tissues, while high abundance expression of them has been detected in roots and vegetative phase of spikes. *TaFAD8.1–3* genes had a high level of expression in leaves and shoots of all wheat developmental stages. However, *TaFAD8.1*, *TaFAD8.3*, and *TaFAD8.2* genes had low, moderate, and high expressions at the reproductive stage in spikes, respectively. The expression patterns of *TaFAD3.1–3* genes were similar and low in all stages and tissues except *TaFAD3.1* with moderate expression in roots at the seedling stage. However, *TaFAD3.4–6* genes showed a high level of expression in all tissues at vegetative and spiked at reproductive stages. In *FAB2* subfamily, *TaFAB2.1–3*, *TaFAB2.6–7*, *TaFAB2.9–11*, *TaFAB2.14*, *TaFAB2.16*, *TaFAB2.18–19*, and *TaFAB2.21–34* revealed a low level of transcripts in all stages and tissues whereas *TaFAB2.4–5*, *TaFAB2.12*, *TaFAB2.15, TaFAB2.17*, and *TaFAB2.20* had a high abundance of transcripts in all developmental stages except *TaFAB2.4* with low expression in roots and spike at growth phase, roots at seedling, roots, and grains at reproductive stage. Likewise, *TaFAB2.8* had high expression in all conditions except seedling phases with moderate expressions. Finally, *TaFAB2.13* showed a low level of transcripts in all conditions except roots at seedling and growth cycles with high expression and leaves/shoots at the growth cycle with moderate expression. Besides, we investigated the expression patterns of *TaFAD* genes to predict their roles responding to biotic and abiotic stresses (Figs. 8 and 9) (Additional file 1: Table S9). In response to powdery mildew pathogen, we observed the up-regulated expression of *TaFAB2.4* after 24 h of treatment. In this observation, *TaFAD8.1–3*, *TaFAB2.1*, *TaFAB2.15*, and *TaFAB2.17* revealed high expressions during the stress. On the other hand, the expression of the *TaFAD3.4*, *TaFAD3.5*, *TaFAD3.6*, *TaFAD3.8*, *TaSLD1–3*, and *TAFAB2.20* (moderate expression) remained unchanged and it has been down-regulated in *TaFAB2.5* and *TaFAB2.12* at 72 h compared with 24 h. The expression of *TaFAD8.1–3*, *TaFAD4.2*, *TaFAD4.3*, and *TaFAD3.5–6* has been increased in response to the stripe rust pathogen CYR31. Although, the transcript level of *TaFAD2.1*, *TaFAD2.6, TaFAD2.8*, *TaFAB2.1*, *TaFAB2.12*, *TaFAB2.17* (high expression) and *TaSLD1–3*, *TaFAD6.1–2*, *TaFAB2.20* (moderate expression) remained unchanged. After 6 h of drought stress, the expression of all *TaFAD* genes has been down-regulated, obviously except *TaFAB2.5*, *TaFAB2.8*, and *TaFAB2.12* that showed up-regulation. The *TaFAD2.1*, *TaFAD2.6*, *TaFAD2.8*, *TaFAB2.5*, *TaFAB2.8*, *TaFAB2.12*, *TaFAB2.15*, *TaFAB2.17*, and *TaFAB2.20* could be up-regulated by heat stress after 6 h. Under drought and heat treatment, the expression of *TaFAD7.1–3* and *TaFAD8.1–3* has been decreased whereas the expression of *TaFAD2.1*, *TaFAD2.6*, *TaFAD2.8*, *TaFAB2.5*, *TaFAB2.8*, *TaFAB2.12 TaFAB2.15*, *TaFAB2.17*, and *TaFAB2.20* have been induced more significantly at 6 h. The expression of 11 *TaFAD* genes has been up-regulated under cold stress including *TaFAD2.6*, *TaFAD2.8*, *TaFAD6.1–2*, *TaFAD8.1–3*, *TaFAB2.15*, *TaFAB2.17*, and *TaFAB2.20* while the transcript level of the *TaFAB2.4*, *TaFAB2.5*, *TaFAB2.8*, *TaFAB2.12*, *TaFAD3.4–6*, *TaFAD4.2–3*, and *TaSLD1–3* was moderate.

### TaFAD2.6 and TaFAD2.8 structural modeling and docking studies

**Fig. 10:**
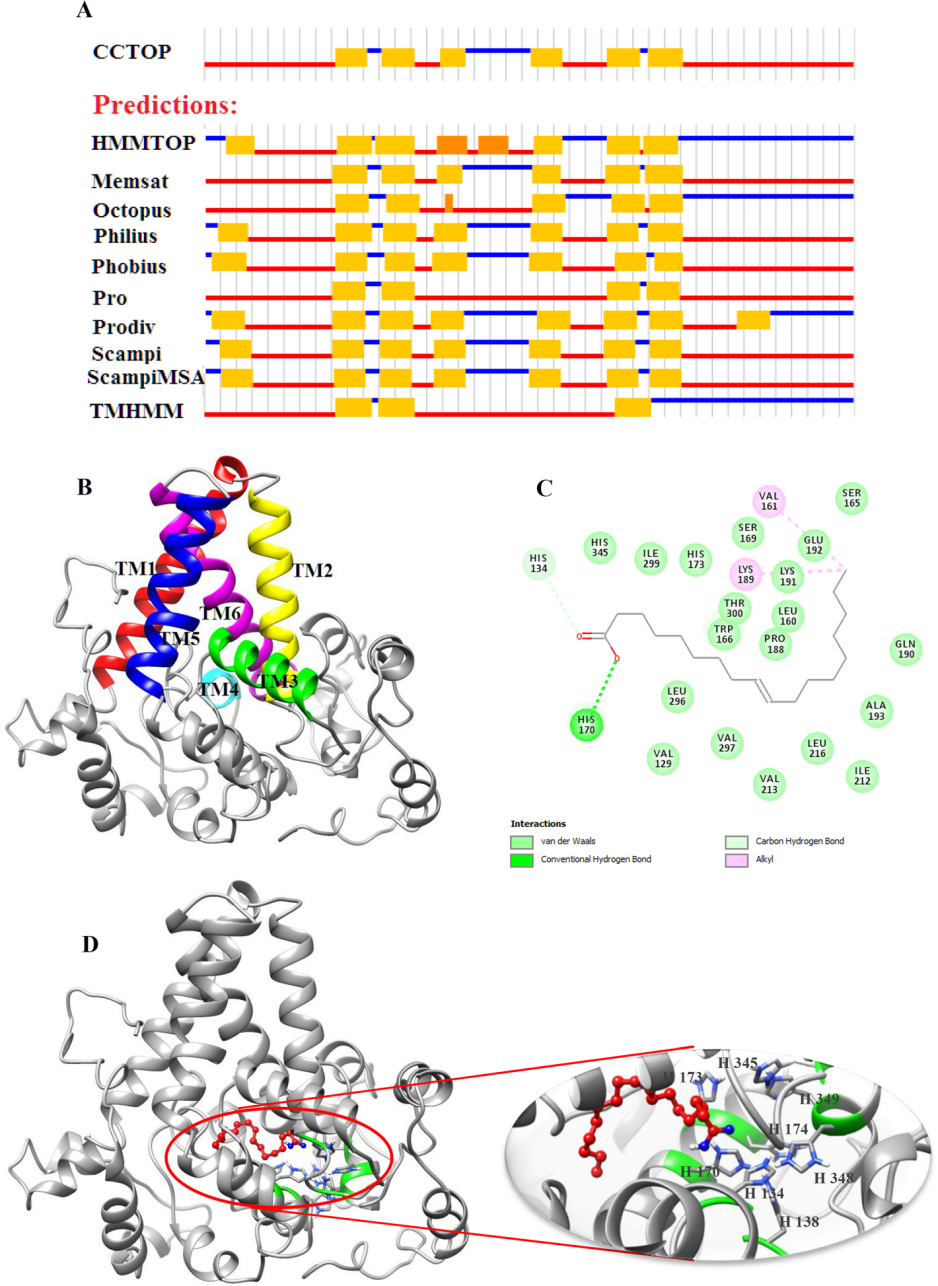
The TaFAD2.6 protein features. The predicted transmembrane (TM) topology from CCTOP prediction (**a**). Three-dimensional model structure of TaFAD2.6 (**b**). Six transmembrane domains were found in the protein structure that is shown in red, yellow, green, cyan, blue, and magenta for TM1, TM2, TM3, TM4, TM5, and TM6, respectively. The residues involved in the TaFAD2.6-oleic acid interaction (**c**). Docking studies of the three-dimensional structure of oleic acid onto the predicted model of TaFAD2.6 (**d**). The ligand and histidine boxes are shown in red and green (side chain in blue), respectively. To analyze ligand-enzyme interaction, AutoDock v4.2.6 has been applied

**Fig. 11:**
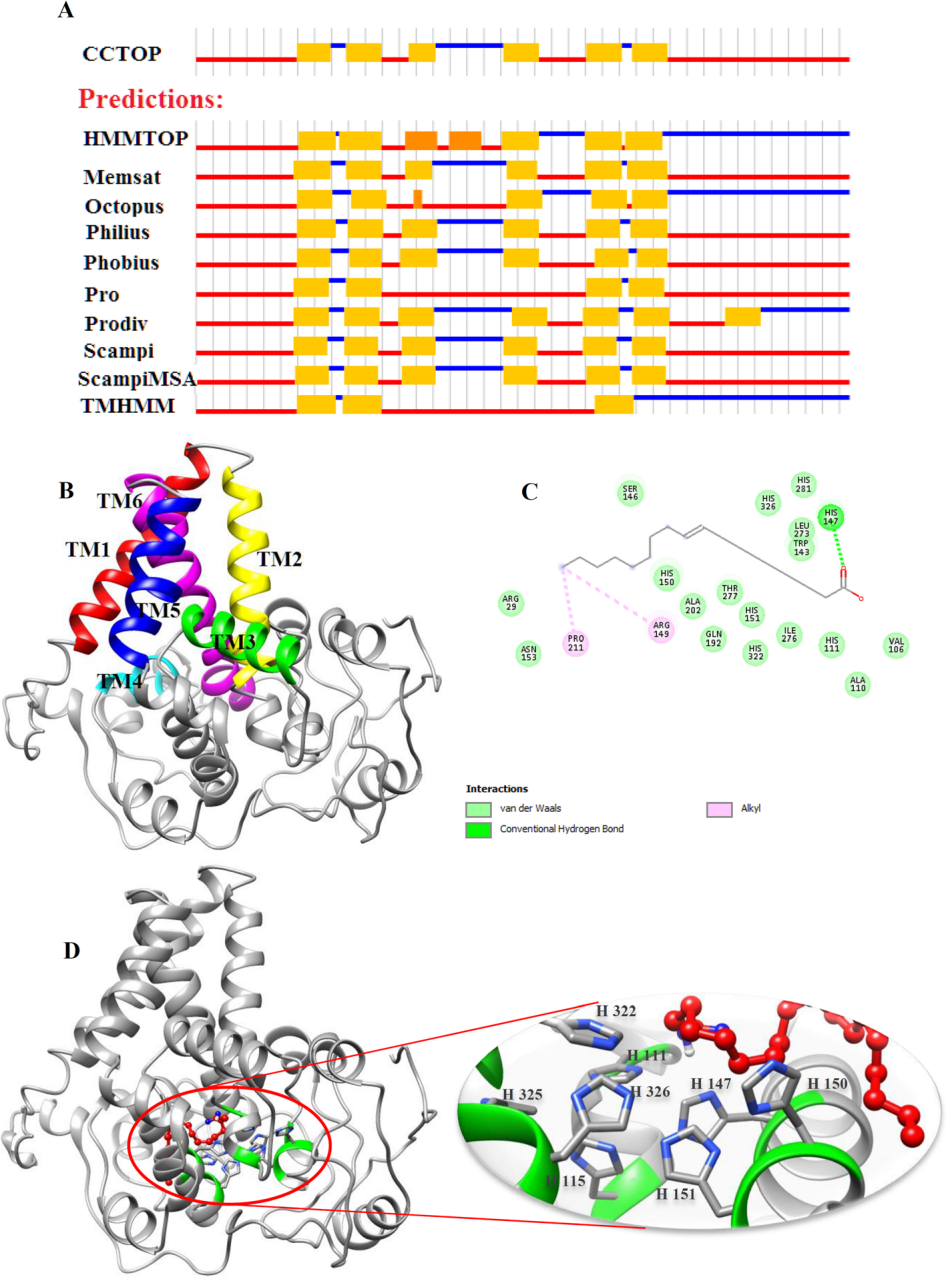
The TaFAD2.8 protein features. The predicted transmembrane (TM) topology from CCTOP prediction (**a**). Three-dimensional model structure of TaFAD2.8 (**b**). Six transmembrane domains were found in the protein structure that is shown in red, yellow, green, cyan, blue, and magenta for TM1, TM2, TM3, TM4, TM5, and TM6, respectively. The residues involved in the TaFAD2.8-oleic acid interaction (**c**). Docking studies of the three-dimensional structure of oleic acid onto the predicted model of TaFAD2.8 (**d**). The ligand and histidine boxes are shown in red and green (side chain in blue), respectively. To analyze ligand-enzyme interaction, AutoDock v4.2.6 has been applied

The FAD2 members have been shown to play important roles in wheat response to environmental stresses. Microsomal omega-six fatty acid desaturases (FAD2) introduce a double bond to the oleic acid (C18:1) carbon chain resulting in linoleic acid (C18:2) [28]. Linoleic acid is a precursor of other polyunsaturated fatty acids, and it is essential for the growth of all eukaryotes [29]. On the other hand, among the FAD2 members, TaFAD2.6 and TaFAD2.8 proteins had the highest expression in all studied stresses. Therefore, in the present study, their molecular structure and interaction of ligand-enzyme have been evaluated. Three-dimensional structures of TaFAD2.6 and TaFAD2.8 proteins have been predicted and refined by I-TASSER and ModRefinder servers, respectively. According to the results of the Ramachandran analysis of primary and refined models, the residue count increased in favored regions from 68.4 to 86.0% and from 69.1 to 87.0% in TaFAD2.6, and TaFAD2.8, respectively, confirming that the refined models are of good qualities. The modeled structure for TaFAD2.6 has 18 α-helices and 19 loops (Fig. 10b) while TaFAD2.8 structure has 19 α-helices and 20 loops (Fig. 11b). Most of the predictor programs predicted that the TaFAD2.6 and TaFAD2.8 proteins have six transmembrane (TM) α-helices (Fig. 10a and Fig. 11a) with a consensus domains of TM1 (Val86-Val109), TM2 (Ala114-Ile132), TM3 (Ser145-Trp158), TM4 (Arg208-Val213), TM5 (Gln254-Val273), TM6 (Trp280-Gln303) for TaFAD2.6 and TM1 (Asp65-Val86), TM2 (ALA91-Ile109), TM3 (Ser122-Trp135), TM4 (Trp197-Asn204), TM5 (Asp236-Ser251), TM6 (Phe255-Thr282) for TaFAD2.8. Three conserved histidine boxes have been found in the TaFAD2.6 and TaFAD2.8. Based on the reports in other plant species, the histidine boxes interact with two irons at the catalytic sites of the FAD2 enzymes [30, 31]. The predicted structures of TaFAD2.6 contains three conserved histidine boxes with eight histidine residues of His134, His138 at position loop 7, His170, His173, His174 at positions α-helix 8, and His345, His348, His349 at position α-helix 16 of the protein structure (Fig. 10d). Likewise, the TaFAD2.8 structure possesses three conserved histidine boxes with eight histidine residues of His111, His115 at position α-helix 4, His147, His150, His151 at positions α-helix 6, and His322, His325, His326 at position α-helix 17 (Fig. 11d). The histidine residues revealed essential catalytic features in plant FADs [32].

Delta-12 desaturase introduces a double bond at the delta-12 position of oleic acid to produce linoleic acid. We carried out docking studies of oleic acid on the refined model structure to assess the ligand specificity of wheat omega-6 desaturases using AutoDock 4.2. Based on the docking simulation with the ligand-enzyme binding energy of − 5.94 kcal/mol, Val129, His134, Leu160, Val161, Ser165, Trp166, Ser169, His170, His173, Pro188, Lys189, Gln190, Lys191, Glu192, Ala193, Ile212, Val213, Leu216, Leu296, Val297, Ile299, Thr300, and His345 of TaFDA2.6 formed closed contacts with the docked oleic acid, while Arg29, Val106, Ala110, His111, Trp143, Ser146, His147, Arg149, His150, His151, Asn153, Gln192, Ala202, Pro211, Leu273, Ile276, Thr277, His281, His322, and His326 were involved in ligand-TaFAD2.8 binding with − 4.75 kcal/mol docking energy. The oleic acid formed a hydrogen band with His170 and His147 in TaFAD2.6 and TaFAD2.8, respectively (Fig. 10c and Fig. 11c). Based on the Fig. 10d, in TaFDA2.6, His134, His138, His170, His173, His174, His345, His348, and His349 residues involved substrate-binding are part of HECGH, HRRHH, and HVAHH histidine boxes. In the TaFAD2.8, His111, His115, His147, His 150, His151, His322, His325, and His326 residues involved in the active site are also part of three conserved histidine boxes (Additional file 1: Table S5). These results suggest that the conserved histidine boxes play critical roles in the binding of the ligand to the active site. The results mentioned above may be useful for future site-directed mutagenesis studies to increase the catalytic efficiency of the FAD2 enzymes and subsequently enhance wheat tolerance to different stresses.

## Discussion

In the current study, 68 members of the *FAD* gene family have been founded in wheat that are significantly larger than the number of *FAD* genes in *A. thaliana* (25) [16], *O. sativa* (19) [33], *Glycine max* (29) [34], and *Medicago trancatula* (20) [35]. Therefore, the expansion of the *FAD* gene family is species-specific in different plants, and this expansion is the result of gene duplication events [36]. One possible reason for this expansion may be related to the larger genome of wheat compared to other species [37]. The uneven distribution of the *FAD* gene family has been observed in wheat, which reported in soybean [34], alfalfa [35], cotton [16], and rapeseed [12] as well. The *FAD* family genes have been widely distributed in the wheat genome, which may indicate that they originated from different ancestors [34]. Studying the structure of genes in life sciences is necessary, and it can be a great guide to identifying the evolution of genes. The stability of genes structure is a prerequisite to maintain their functional role, while variation in gene structure is essential for the functional evolution of gene families [38]. In the present survey, segmental duplication (83.66%) of the *FAD* gene family in wheat was far more than tandem duplication (16.33%). In a study on duplication of 50 large gene families in Arabidopsis, it was concluded that there was a negative relationship between tandem and segmental duplications and when each of these duplication events was greater, the other type played the least role in the expansion of gene family members [39]. It should be noted that if the duplicated genes are maintained in evolution, they generally change in their regulatory or coding region. Changing in coding regions, especially if they cause changes in function, are caused by amino acid substitution or changes in exon-intron structure [40]. The study of the selection pressure of the *FAD* gene family in wheat showed that a strong purifying selection has been occurred indicating the importance of the functional role of these genes. Generally, the divergence time of tandem duplication is more recent phenomena than segmental duplication and stress-responsive genes tend to located on tandem clusters [41, 42].

There are two major groups of desaturases, including soluble desaturase and membrane-bound desaturase [34]. Based on the phylogenetic study, five desaturase subfamilies have been identified, including delta-15 desaturase (FAD3/FAD7/FAD8), stearoyl-ACP desaturase (FAB2), delta-12 desaturase (FAD2/FAD6), delta-3 desaturase (FAD4), and front-end desaturase (DES/SLD). To date, stearoyl-ACP desaturase is the only identified soluble desaturase. The phylogenetic tree and the type of clustering of FAD proteins are consistent with the other plants, including sunflower [10], cucumber [43], and soybean [34]. Each cluster had similar amino acid compositions, and it can be concluded that the phylogenetic distribution of wheat FAD proteins is related to their motif contents. All members of the FAB2 cluster contained motifs 1, 2, 4, and 9 except TaFAB2.4 with motifs 8 and 10. The *FAD2/FAD6* subfamily had common motifs 1, 2, 16, and 18. The members of the FAD6 group contained specific motifs 20, 22, and 28, while the members of the FAD2 cluster had special motifs 11, 12, 14, and 17 which may be the reason for the separation of these groups in the dendrogram. All members of the *FAD* subfamily contained motif 1 except DES, SLD, and TaFAD4. The main difference between TaSLD1–3 and TaDES1–3 was the presence of specific motifs 8, 19, 29, and 30 in TaSLD1–3. On the other hand, the FAD4 group had only two motifs (10 and 18); thus, it was completely separated from the other groups of *FAD* subfamily. The members of the FAD3/FAD7/FAD8 group had six common motifs as well. TaFAD7 and TaFAD8 had quite similar motifs. Therefore, their clusters were closer to each other than to FAD3. The protein sequence of FAD7 and FAD8 is very similar. The transcript levels of FAD7 and FAD8 increase at high temperatures and low temperatures, respectively [16, 44]. Two and three His-boxes have been observed in the *FAB2* and *FAD* subfamilies, respectively. The identified His-boxes in the *FAB2* subfamily are related to their active sites [7]. All members of the *FAD* subfamily contain three His-boxes except TaFAD2.4, which had two His-Boxes and also found in a separate clade from the other TaFAD2 in the phylogenetic tree. The third His-box was located in the carboxy-terminus of TaFAD proteins, and its consensus sequence was H/QX_2_HH, which is similar to FAD protein sequences in various plants [7]. The number of residues between the first and second His-boxes varied between different members of the *TaFAD* subfamily (Additional file 1: Table S5). For instance, the amino acid length between His-box 2 and His-box 3 was 111 or 171 in TaFAD2.1–11, 156 in TaFAD6.1–2, 162 in TaFAD3.1–6, 163 in TaFAD7.1–3 and TaFAD8.1–3, 173 in TaSLD1–3, and 127 in TaDES1–3. The exception was the TaFAD4 cluster, in which this length was only 25 amino acids. Synthesis of fatty acids occurs in plastids but their desaturation is in plastids and endoplasmic reticulum [45]. In most studies, subcellular localization of the *FAB2* and *FAD* subfamilies is chloroplast and chloroplast/ER, respectively. However, according to our analysis, the subcellular localization of the *FAB2* subfamily was both chloroplast and mitochondria (Additional file 1: Table S1) which is in line with Diaz et al. (2018) findings. Likewise, it was observed that all members of TaFAD4 and DES/SLD clusters were localized in the plasma membrane. The *FAD* gene family in wheat revealed all three types of splicing phase (zero, one, and two). The splicing phase of wheat *FAD* gene family was almost similar to those of other plants, indicating a high degree of the *FAD* genes conservation during evolution [21, 34, 35, 43]. Eighteen members of the wheat *FAD* gene family lacked introns. In eukaryotic genomes, there are three ways to form intronless genes including horizontal gene transfer from prokaryotes, duplication of intronless genes, and retrotransposons [46].

Investigating the promoter regions helps to understand the interaction and function of genes. Transcription factors play an essential role in the regulation of signaling pathways in response to abiotic stresses. The proteins of these factors bind to the promoter of the target genes, regulating their induction or repression activity [47]. The TGACG and CGTCA motifs are on the genes that respond to methyl jasmonate [48]. ABRE and MBS are also the regulatory elements in response to abscisic acid (ABA) and drought, respectively. Jasmonate involves in seed germination, senescence, and response to biotic and abiotic stresses [49]. ABRE motif with the sequence of TACGGTC activates in response to ABA, resulting in plant tolerance against drought and salinity. The high abundance of cis-acting elements related to response to jasmonate, ABA, drought, cold, pathogen, auxin, gibberellin, and ethylene suggest that the *TaFAD* genes are involved in response to a wide range of stress. However, the existence of a particular cis-acting element in the promoter of a gene is not a definite reason for the expression of that gene in response to the same stress or hormone. This could be due to the complexity of the mechanism of gene expression regulation and the limitation of bioinformatics tools in predicting the cis-acting elements of the promoters [50]. Therefore, using experimental methods such as qRT-PCR is essential for identifying functional regulatory elements in *TaFAD* promoters. In general, it is known that desaturases can respond to different stresses. For instance, when plants exposed to low temperatures, changes in the expression of their *FAD* genes occur that result in changes in membrane lipid fluidity and eventually increase cold tolerance [51]. It has been reported that the changes in the desaturation of fatty acids result from the regulation of the *FAD* genes in response to different stresses [23].

SSRs are tandem repeats of 1–6 nucleotides that have been reported to play important roles in regulating gene expression [52]. The location of tri-nucleotide SSRs in untranslated regions can induce gene silencing. Their distribution in the coding regions also affects transcription, translation, and gene function [53]. In the current study, the highest abundance has been observed for the tri-nucleotide repeats (50%) in *TaFADs* that is in line with previous studies in the wheat genome [54, 55]. The dominant SSRs are different in various plant species. For instance, in monocots (except maize), most of the dominant SSRs are CCG/CGG/CGC/GCG/GCC/GGC, while most of the dominant SSRs in dicots (except Arabidopsis) are AAT/ATT/ATA/TAT/TAA/TTA [56]. Based on the results of Qin et al. (2015), the type of dominant SSRs is taxon-dependent, and the abundance of AT in the dicots genome is higher than that of the monocots. In the future, SSR polymorphisms in *TaFADs* may be investigated in different cultivars and may be suitable for marker-assisted selection development in wheat breeding programs to select genotypes with a better quality of germ oil. MicroRNAs (miRNA) are kind of non-coding small RNAs usually 19–24 bp in length. They play an important role in controlling post-transcriptional changes. miRNAs are widespread in plants, animals, and viruses. Likewise, they play important roles in plant development and responses to biotic and abiotic stresses [57]. Predicting the target of miRNAs using bioinformatics tools has made it convenient and fast. In *FAB2*, *FAD2*, and *FAD4* subfamilies, 24, 8, and 1 transcript were targeted by wheat miRNAs, respectively. However, all members of *FAD7*, *FAD8*, and *SLD* transcripts were targeted by miRNAs. In wheat, studies have been carried out to identify miRNAs and their crucial role in stress tolerance [58–61]. miR159 plays a role in leaf development. The members of the MYB family influence response to stress through hormones and signaling networks [62]. miR160 can play a role in response to cold stress by targeting MYB3 transcription factors [63]. They also involve in root development [64]. In the current study, the putative target of miR160 was *TaFAD8.2* with an auxin-responsive element in the promoter. The accumulation of auxin-responsive elements through miR160 downregulation enhances the response to auxin and resulting in enhancement of root and leaf development [65]. Induction of miR408 expression occurs under root and leaf dehydration stress [66]. miR395 and miR530 are more expressed in the leaves of plants and control the expression of the genes related to plant development [59]. Therefore, *TaFAB2.3* and *TaFAD8.3* are probably involved in leaf development. miR1120 is important in regulation of the meiosis and early anther development in wheat [67], thus, the *TaFAB2.15* may be involved in the reproductive development of wheat plants. miR164 is also essential for reproductive development and responding to various abiotic stresses [68, 69] that targets *TaDAD2.5* in this study. miR408 is involved in response to copper deprivation [70] and drought stress [71], which has also been demonstrated in wheat [72]. Likewise, miR1139 is involved in wheat’s response to phosphate deficiency [73]. miR9659 and miR9657b are seed-specific [74], whereas miR9666b and miR9670 affect grain development in wheat [75]. Therefore, *TaFAD2.1*, *TaFAD2.4*, *TaFAD2.6–8*, *TaFAD2.10–11* are probably involved in reproductive development. According to other studies, miR5048 and miR5049 are involved in response to drought [75, 76]. miR5384 can regulate the two pathways of cell death and growth [77]. Finally, miR9772 and miR9678 are essential for the response to heat stress [78] and germination of wheat seeds [79].

Gene expression analysis provides important information regarding the function of the identified genes. The expression profiles of *TaFADs* have been investigated under various biotic and abiotic stresses. The highest expression in all developmental stages and tissues was related to *TaFAB2.*5, *TaFAB2.12*, *TaFAB2.15*, *TaFAB2.17*, *TaFAB2.20*, *TaFAD2.1*, *TaFAD2.6*, and *TaFAD2.8*. The expression pattern of *TaFAD2.8* and *TaFAB2.*5 was similar to high frequency in all tissues except shoots/roots at the growth cycle with moderate abundance. Sever changes in the expression of the *TaFAB.13* gene in all of the developmental stages have been observed. The expression of this gene was low in all cycles and tissues, while the moderate and high levels of transcripts have been observed in shoots/root at the growth cycle and roots at vegetative and seedling stages, respectively. Other *FAB2* subfamily members showed high level of expression in all tissues except *TaFAB2.1–3*, *TaFAB2.6–7*, *TaFAB2.9–11*, *TaFAB2.14*, *TaFAB2.16*, *TaFAB2.18–19*, and *TaFAB2.21–34*. The expression pattern of *TaFAB2.4* of the *FAB2* subfamily was similar to *TaFAD6.1–2* and *TaFAD8.1–3* genes. They revealed drastic changes in gene expression as high expression at all phases of shoots/ leaves and reproductive phases of spikes, but low expression in roots and grains tissues. The expression patterns of the *TaFAD7.1–3* gene were almost similar with high transcripts level in roots (at all developmental cycles), grains, and spikes (at vegetative phase). These findings contradict the results of Nishiuchi et al. (1997). They reported no expression of *AtFAD7* genes in Arabidopsis roots [80]. However, our results are in agreement with Chi et al. (2011) findings. Based on their study, *GmFAD7* had expression in all tissues of soybean [34]. *TaFAD8.1–3* genes also demonstrated the same expression patterns, and they had the highest expression in shoots and leaves. These results are inconsistent with those of Soria-García et al. (2019). They detected *AtFAD8* in Arabidopsis leaves, not roots [81]. In the case of *TaFAD3.1–3* genes, the expression was relatively low in all tissues, whereas the expression level of *TaFAD3.4–6* was highly abundant in all tissues at the growth stage and in spike at the reproductive phase. The expression of *TaFAD2.1*, *TaFAD2.6*, and *TaFAD2.8* genes was high in all tissues while the expression of *TaFAD2.2–5*, *TaFAD2.7*, and *TaFAD2.9–11* was found in low abundance in all tissues and phases. The expression patterns of duplicated paired genes was mostly similar except *TaFAD2.5/TaFAD2.6*, *TaFAD2.8/TaFAD2.9*, *TaFAD2.1/TaFAD2.2*, *TaFAD2.1/TaFAD2.3*, *TaFAD2.6/TaFAD2.7*, *TaFAD2.4/TaFAD2.6*, *TaFAD2.8/TaFAD2.10*, and *TaFAD2.8*/*TaFAD2.11*. Different expression patterns of TaFAD genes under biotic and abiotic stresses indicating their different functions. The expression patterns of the *TaFAB2* subfamily were different under biotic stress (Fig. 8). For instance, the *TaFAB2.15* and *TaFAB2.17* constantly expressed at high levels, whereas other *FAB2* genes showed a minimum expression except for *TaFAB2.12* and *TaFAB2.20* with moderate to high expressions. Therefore, they have different roles in plant response to pathogen attacks. The expression of *TaFAD8.1–3* has been induced in response to pathogen infection as well. Omega 3 fatty acid desaturases convert dienoic fatty acids to trienoic fatty acids, including linolenic acid, which is subsequently converted to jasmonic acid (JA) [82]. Accumulation of JA is essential in plant response to pathogen infection [82]. The expression of *TaFAD2.1*, *TaFAD2.6*, and *TAFAD2.8* was also increased during biotic stress. The *FAD2* genes appear to be involved in response to pathogen attack through increasing biosynthesis of linoleic acid and palmito-linoleic acid in olive [83]. Temperature stress can affect plant yield [16]. Based on the other studies, several members of the *FAD* gene family play important roles in response to temperature stress in various plant species [16, 43, 84–86]. According to the Fig. 9a, the expression levels of *TaFAD2.1–2*, *TaFAD2.6*, *TaFAD2.8*, *TaFAB2.5*, *TaFAB2.8*, *TaFAB2.12*, *TaFAB2.15*, *TaFAB2.17*, and *TaFAB2.20* was increased after 6 h of heat treatment relative to their expression after 1 h of heat stress while the expression of other *TaFADs* was almost constant. Likewise, the expression of *TaFAD2.6*, *TaFAD2.8*, *TaFAB2.15*, *TaFAB2.17*, and *TaFAB2.20* was high during 2 weeks of cold treatment. The results suggest that *TaFAD2.6*, *TaFAD2.8*, *TaFAB2.15*, *TaFAB2.17*, and *TaFAB2.20* might play an important role in the response of wheat plants to temperature stress. Furthermore, the expression of *TaFAD6.1–2* and *TaFAD8.1–*3 was high in response to cold stress. These results are in line with the results reported by Wang et al. (2006). They ascertained that the over-expression of *FAD8* in rice resulted in a reduction in the cold stress damage [87]. Along with the results reported by other researchers, the expression of most *TaFAD* genes was more related to low temperature than to heat stress response [16, 86, 88, 89]. The expression level of the *TaFAD2.8* under low temperature is much higher than that of the other *TaFAD* genes, suggesting that this gene may be related to enhancing cold tolerance in wheat. The different expression patterns of the duplicated genes illustrated the theory of divergence that these duplicated genes could be the result of two mechanisms; 1- subfunctionalization, and 2- neofunctionalization. In the subfunctionalization mechanism, some of the functional aspects of new genes are different from the function of the parental genes [90]. However, in the neofunctionalization process, the new gene has a different role due to changes in its amino acid composition compared with the parental gene [91]. Taken together, the results suggest that members of the wheat *FAD* gene family may maintain their main functions or obtain different roles during evolution. The use of accurate expression assessment methods such as northern blot and qRT-PCR are necessary to determine the exact expression patterns of the wheat *FAD* gene family at various tissues and developmental stages under biotic and abiotic stresses.

## Conclusions

In recent years, using bioinformatics tools and computational biology methods is essential to clarify complicated biological problems in genomics, proteomics as well as in metabolomics. Therefore, researchers can identify various stress-related elements responding to environmental changes and subsequently develop improved crop plants in terms of quality and productivity, showing enhanced tolerance against biotic and abiotic stresses. On the other hand, fatty acid desaturases have essential roles in plant development and response to various stresses. Therefore, in the present study, 68 *TaFAD* genes were identified using bioinformatics approaches that they revealed 0 to 10 introns with high structural diversity. Some of *TaFAD* genes were intronless, and probably their genesis was based on the horizontal gene transfer from prokaryotes or retrotransposons. Based on the phylogenetic study, *TaFAD* genes were divided into five groups, including *FAB2*, *FAD2/FAD6*, *FAD4*, *DES/SLD*, and *FAD3/FAD7/FAD8*. The analysis of the mechanism of gene family expansion revealed that tandem and segmental duplications have occurred. Based on the results of the Ka/Ks ratio, the function of most of the duplicated *TaFAD* genes has been maintained during the evolution due to negative selection. Promoter analysis showed hormones and stresses-responsive elements in the *TaFAD*s promoters suggesting the role of them in plant development and responses to biotic and abiotic stresses. Likewise, the *TaFAD* gene expression patterns indicated their functional roles in different tissues and developmental stages under environmental stresses in wheat. The expression patterns of genes resulting from tandem duplication suggested new functional roles for some of the duplicated genes. In addition, several residues in the active site of TaFAD2.6 and TaFAD2.8 in close contact with the docked oleic acid were found based on docking simulations which could be useful in future site-directed mutagenesis studies to improve the catalytic efficiency of the FAD2 enzymes and thereby to enhance the resistance of wheat to different stresses. This study was the first step in understanding the potential role of the *TaFAD* genes and could be useful in future studies on the specific role of each of the *TaFAD* genes at different developmental stages and responses to biotic and abiotic stresses.

## Methods

### In silico identification of *FAD* genes

FAB enzymes have the functional FA_desaturase 2 domain, while the FAD enzymes have the FA_desaturase or TMEM189 domains. Therefore, to determine the *FAD* gene family in wheat, the HMM profile of FA_desaturase (PF00487), FA_desaturase 2 (PF03405) or TMEM189 (PF10520) domains were obtained from Pfam database [92], and hmmSearch tools in HMMER server [93] has been used to find wheat FAD proteins in Ensembl Plants database [94]. Default parameters including significance E-values 0.01 for sequence, 0.03 for hit matches, and reporting E-values 1 for both sequence and hit. Molecular weight, length, and theoretical isoelectric points of wheat FAD were calculated using the ProtParam tool of the ExPASY Bioinformatics Resource Portal [95]. To recognize the cellular localization of proteins, CELLO, and DeepLoc have been applied [96, 97].

### Evolutionary relationships of wheat *FAD* gene family

We used ClustalX 2.0.8 software to investigate the evolutionary relationships of the *FAD* gene family using full-length protein sequence alignment of wheat, Arabidopsis, rice, and soybean (Additional file 2). A phylogenetic tree of FAD proteins was constructed using MEGA 7 [98] based on Neighbor-joining (NJ) method with 1000 bootstraps [99].

### Duplication of *FAD* members and selection pressure

The location image of *FAD* genes on the wheat chromosomes has been generated using TBtools [100]. Genes on the same chromosome with a maximum spacing of 10 genes have been considered as tandem duplication [101]. Likewise, two criteria have been considered to identify segmental duplication, including the identity of the aligned region more than 90% and the coverage of alignment more than 90% compared with longer genes [102]. Also, we computed the type of selection pressure on the tandem and segmental duplication genes, substitution rates of synonymous (Ks) and non-synonymous (Ka) by DnaSP ver. 5 software [103]. Then, the type of selection on genes has been determined using the Ka/Ks rate. Divergence time (T) of the duplicated genes has been estimated using the T = Ks/2λ (MYA) formula that λ is 6.5 × 10^− 9^ in wheat [104].

### Investigation of exon-intron structure and conserved motifs

Exon-intron distributions and the type of splicing phase for wheat *FAD* genes have been appraised using a gene structure display server (GSDS 2.0) [105]. To find specific motifs of the *FAD* gene family, Multiple Em for Motif Elicitation (MEME 5.0.5) was used [106]. Various parameters, including a minimum length of motifs (6 amino acid) and a maximum length of motifs (200 amino acid), have been considered. Identification of 10 and 30 motifs have been taken into account for *FAB2* and *FAD* subfamilies, respectively. Pfam and SMART databases have been used to evaluate the function of the motifs above [107, 108].

### Promoter analysis and prediction of simple sequence repeats (SSR) markers and miRNAs targets

1500 bp upstream of starting codon (ATG) of *FAD* genes were obtained from Ensemble Plants database [109], and identification of cis-regulatory elements was carried out using PlantCare [110].

SSR markers have been identified in *TaFAD* gene sequences using the BatchPrimer3v1.0 server [111]. To find *TaFAD*-targeted miRNAs, CDS sequences of them were analyzed in the psRNATarget database by considering default parameters.

### Digital gene expression analysis

To assess the expression profile of the wheat *FAD* genes, wheat gene expression data in the expVIP database [112] was applied, including RNA-seq data related to reproductive and vegetative shoot, leaf, root, spike, and grain tissues. Likewise, the expression profile of *TaFADs* under different stresses, including drought, cold, heat, and pathogen infection, has been evaluated. The obtained log10 (TPM + 1) value related to *FAD* genes expression was used, and heat map via an Amazing Heatmap in TBtools software [100].

### TaFAD2.6 and TaFAD2.8 structural modeling and validation

In situ full-length atomic structure of TAFAD2.6 and TaFAD2.8 proteins were constructed by iterative template-based fragment assembly simulations to predict protein structures in the I-TASSER server [113]. The best models from I-TASSER were further refined by ModRefinder software [114]. The predicted structures were then validated via Ramachandran plot by measuring the backbone dihedral phi (ϕ) and psi (Ψ) angles using the PROCHECK module of the PDBSum server [115], and RAMPAGE server has been applied for further confirmation [116]. The transmembrane α-helices of the TAFAD2.6 and TaFAD2.8 has been predicted using a CCTOP program (http://cctop.enzim.ttk.mta.hu/).

### Molecular docking

The structure of oleic acid ligand has been obtained from the PubChem database [117] and converted into PDB format using Discovery Studio software. An enhanced version of the COACH server (COACH-D) has been used to predict protein-ligand binding site [118]. The server, as mentioned above, uses five methods to predict protein-ligand binding sites; four methods are template-based, including TM-SITE [119], S-SITE [119], COFACTOR [120], and FINDSITE [121] whereas the latter method (ConCavity) is structure-based [122]. Then, the results of each method have been combined using the COACH algorithm [119]. To analyze ligand-enzyme interaction, AutoDock v4.2.6 has been applied [123]. To prepare grid maps, the Auto Grid program developed with AutoDock has been used. The grid box size for x, y, and z was set at 60, 60, and 60 Å, respectively. The grid center for x, y, and z was set at 63.065, 63.843, and 63.387 Å, respectively with a grid spacing of 0.375 Å. To find the best conformers, Lamarckian Genetic Algorithm (LGA) has been selected. For ligand a limit of 100 conformers was considered during the docking process. Most of docking processes were carried out with AutoDock4’s default parameters [123]. Population size was set at150, the maximum number of tests at 2,500,000, the maximum number of generations at 27,000, the maximum number of automatically surviving top individuals 1, gene mutation rate 0.02 and crossover rate 0.8. The interaction of the enzymes and substrates has been demonstrated in 2D and 3D using Discovery Studio Visualizer and Chimera software (Avilable on https://github.com/Amin62123/Chimera/tree/TaFAD) [124], respectively.

## Supplementary Information

**Additional file 1: Table S1** Features of wheat ATG proteins. **Table S2** Ka/Ks analysis of the *TaFAD* duplicated paired genes. **Table S3** Putative conserved motifs of the *TaFAB2* subfamily. **Table S4** Putative conserved motifs of the *TaFAD* subfamily. **Table S5** Conserved histidine-rich boxes of FAD in wheat. **Table S6** List of Cis-acting regulatory elements in the *TaFAD* promoter. **Table S7** Simple sequence repeats detected in *TaFAD* genes. **Table S8** Putative *TaFAD*-targeted miRNA. **Table S9** Gene expression data of *TaFADs.*

**Additional file 2.** The full-length protein sequence of wheat (Ta), Arabidopsis (At), rice (Os), and soybean (Gm).

## Data Availability

All data generated or analyzed during this study are included in this published article and its supplementary information files.

## Abbreviations

ABA: Abscisic acid
Chr: Chromosome
FAD: Fatty acid desaturase
JA: Jasmonic acid
LGA: Lamarckian Genetic Algorithm
MW: Molecular weight
pI: Isoelectric point
SSRs: Simple sequence repeats
TaFAD: Wheat fatty acid desaturase
