# Supplementary material for: Identification, evolution, expression, and docking studies of fatty acid desaturase genes in wheat (Triticum aestivum L.)
Source: BMC Genomics. 2020 Nov 10;21:778. doi: 10.1186/s12864-020-07199-1 (PMC7653692; doi:10.1186/s12864-020-07199-1)
Supplement: Supplementary file 2 — Additional file 2. The full-length protein sequence of wheat (Ta), Arabidopsis (At), rice (Os), and soybean (Gm). [file 12864_2020_7199_MOESM2_ESM.doc]

**Additional file 2** Full-length protein sequence of wheat (Ta), *Arabidopsis* (At), rice (Os), and soybean (Gm).

>TaFAB2.5

MAFRACLPSHKASPSPSVAQRRAGNGAPPVVVMASTMNEVKTAKKPYAPPREVHLQVMHS

LPAQKQEIFDSLQSWARDNLLNLLKPVEKSWQPQDFLPDPSSEGFYDEVKELRERAKEIP

DDYFVCLVGDMVTEEALPTYQTMLNTLDGVRDETGASPTAWAVWTRAWTAEENRHGDLLN

KYMYLSGRVDMRQIEKTIQYLIGSGMDPGTENNPYMGFLYTSFQERATFISHGNTARHAK

QFGDLKLAQICGTIAADEKRHETAYTKIVEKLFEIDPDYTVLAFADMMRKKISMPAHLMY

DGEDDNLFEHFSSVAQRLGVYTAKDYADILEFLVQRWKVADLTGLSGEGRRSQDYVCTLA

TRFRRLDERAQARAKQGPVVPFSWVYDRKVQL

>TaFAB2.8

MAFRACLPSHKASPSPSVAQRRASNGAPPVVAMASTMNEVKTAKKPYAPPREVHLQVMHS

LPAQKQEIFDSLQSWARDNLLNLLKPVEKSWQPQDFLPDPSSEGFYDEVKELRERAKEIP

DDYFVCLVGDMVTEEALPTYQTMLNTLDGVRDETGASPTAWAVWTRAWTAEENRHGDLLN

KYMYLSGRVDMRQIEKTIQYLIGSGMDPGTENNPYMGFLYTSFQERATFISHGNTARHAK

QFGDLKLAQICGTIAADEKRHETAYTKIVEKLFEIDPDYTVLAFADMMRKKISMPAHLMY

DGEDNNLFEHFSSVAQRLGVYTAKDYADILEFLVQRWKVADLTGLSGEGRRSQDYVCTLA

TRFRRLDERAQARAKQGPVVPFSWVYDRKVQL

>TaFAB2.12

MAFRACLSSHKASPSPSVAQRRASNGAPPVVAMASTMNEVKIAKKPYAPPREVHLQVMHS

LPAQKQEIFDSLQSWARDNLLNLLKPVEKSWQPQDFLPEPSSEGFYDEVKELRERAKEIP

DDYFVCLVGDMVTEEALPTYQTMLNTLDGVRDETGASPTAWAVWTRAWTAEENRHGDLLN

KYMYLSGRVDMRQIEKTIQYLIGSGMDPGTENNPYMGFLYTSFQERATFISHGNTARHAK

QFGDLKLAQICGTIAADEKRHETAYTKIVEKLFEIDPDYTVLAFADMMRKKISMPAHLMY

DGEDDNLFEHFSSVAQRLGVYTAKDYADILEFLVQRWKVADLTGLSGEGRRSQDYVCTLA

TRFRRLDERAQARAKQGPVVPFSWVYDRKVQL

>TaFAB2.17

MASRMALRPHGVTPPLAAAGRAGRRSGSVRVLAIASSASAKIESKKTFAPPKEVHVQVTH

SMPPQKMEIFQSLDGWARDNLLLHLKPVEKCWQPQDFLPDPASDGFHDEVKELRERAKEI

PDDYLVCLVGDMITEEALPTYQTMLNTLDGVRDETGASPTAWAVWTRAWTAEENRHGDLL

NKYLYLTGRVDMRQIEKTIQYLIGSGMDPRTENNPYLGFIYTSFQERATFISHGNTARHA

KDFGDLKLAQICGIIASDEKRHETAYTKIVEKLFEIDPDGTVLALADMMRKKIAMPAHLM

FDGQDEKLFDHFSMVAQRLGVYTARDYADILEFLVGRWKVPELTGLSGEGHKAQDYLCTL

AGRIRKLDERAQSRAKQAGKMPFSWVYGREVQM

>TaFAB2.15

MAAAVPLILLLRSGANTTVVEAEPLPFNFLRAQATSFPVRIDPSPEPPAPMVRFAFPPHR

TRTADRQSVRSTLNSAGAFMLRAASVTVASHPIPEWLRARYHSHAVVSINCLLFQYWPAG

GRVRGSRWFRIESKKTFAPPKEVHVQVTHSMPPQKMEIFQSLDGWARDNLLLHLKPVEKC

WQPQDFLPDPASDGFHDEVKELRERAKEIPDDYLVCLVGDMITEEALPTYQTMLNTLDGV

RDETGASPTAWAVWTRAWTAEENRHGDLLNKYLYLTGRVDMRQIEKTIQYLIGSGMDPRT

ENNPYLGFIYTSFQERATFISHGNTARHAKDFGDLKLAQICGIIASDEKRHETAYTKIVE

KLFEIDPDGTVLALADMMRKKIAMPAHLMFDGQDEKLFDHFSMVAQRLGVYTARDYADIL

EFLVGRWKVPELTGLSGEGHKAQDYLCTLAGRIRKLDERAQSRAKQAGKMPFSWVYGREV

QM

>TaFAB2.20

MASRMALRPHGVTPPLAAGRGGRRNGSVRVLAVASSASAKIESKKTFAPPKEVHVQVTHS

MPPQKMEIFQSLDGWARDNLLLHLKPVEKCWQPQDFLPDPASDGFHDEVKELRERAKEIP

DDYLVCLVGDMITEEALPTYQTMLNTLDGVRDETGASPTAWAVWTRAWTAEENRHGDLLN

KYLYLTGRVDMRQIEKTIQYLIGSGMDPRTENNPYLGFIYTSFQERATFISHGNTARHAK

DFGDLKLAQICGIIASDEKRHETAYTKIVEKLFEIDPDGTVLALADMMRKKIAMPAHLMF

DGQDEKLFDHFSMVAQRLGVYTARDYADILEFLVGRWKVPELTGLSGEGHKAQDYLCTLA

GRIRKLDERAQSRAKQAGKMPFSWVYGREVQM

>TaFAB2.18

MQAQGILRVPGHLPTALPLPRRQCRRVSAAAAVAAAAPSVQRGVTHSMPPEKAEVFQSLR

GWAAGSLLPLLRPVEDIWQPADFLPDSSSEMFEHEVRELRARAAALPDEYFVVLVGDMVT

EEALPTYQTMINTLDGVRDETGASACPWAVWTRAWTAEENRHGDVLNKYMYLSGRVDMRM

VEKTVQYLIGSGMDPRTENNPYLGFVYTSFQERATAVSHGNTARLAKAHGDDVLARTCGT

IAADEKRHETAYSRIVEQLLRLDPEGAMLAIADMMRKRITMPAHLMHDGSDMDLFEHFAS

VAQRLGVYTAQDYTDIVEFLVKRWKLEALEGGLSSEGRRARDFVCGLAPRMRRAAERAAD

RAKKDEPRKVKFSWIFDREVVV

>TaFAB2.13

MQAQGLLRVPGHPAAARPLPRRQCRVSAVAAAAPSVQRGVTHSMPPEKAEVFQSLRGWAA

GSLLPLLRPVEDIWQPADFLPDSSSEMFEHEVRELRARAAALPDDYFVVLVGDMVTEEAL

PTYQTMINTLDGVRDETGASACPWAVWTRAWTAEENRHGDVLNKYMYLSGRVDMRMVEKT

VQYLIGSGMDPRTENNPYLGFVYTSFQERATAVSHGNTARLARAHGDDVLARTCGTIAAD

EKRHETAYSRIVEQLLQLDSDGAMLAIADMMRKRITMPAHLMHDGSDMSLFEHFASVAQR

LGVYTAQDYTDIVEFLVKRWKLEALEGGLSGEGRRARDFVCGLAPRMRRAAERAADRAKK

DEPRKVRFSWIFDREVVV

>TaFAB2.22

MYLMAYMPSCGTFHAPLVSSCLRRKFTVVATASKAKVGTPKKASTQVRFTQPFPPEKKEI

FDSLERWAEENILVLLKPVEKSWQPQDYLPDPSSDGFYDEVKELRERAKEIPDDYLVCLV

GDMVTEEALPTYQTMLNILDGGVGDDTGTSPASWAVWTRAWTAEENRHGDLMNKYMYLTG

RVDMRQIEKTIQYLLGAGMDPKTEGNPYEGYIYTSFQERATFISHGNTARHARKYGDLKL

AQICGTIAADEKRHETAYTKIVEKLFEVDPDYTVLAFAAMMRKKVTMPAHLMYDGQDDNL

FEHFSAVAQRLGVYTAMDYADILDFLVQRWNVANLTGLSGEGRRAQDFLCSLGPRFRKLE

ERAQGRAKQLPVVPFSWIHGRQVQL

>TaFAB2.26

MYLMAYMPSCGTFHAPLVSSCLRRKLTVVATASKAKVGTPEKASTQVRFAQPFPPEKKEV

FDSLERWAEDNILVLLKPVEKSWQPQDYLPDPSSDGFHDEVKELRERAKEIPDDYLVCLV

GDMVTEEALPTYQTMLNILDGGVGDDTGTSPASWAVWTRAWTAEENRHGDLMNKYMYLTG

RVDMRQIEKTIQYLLGAGMDPKTEGNPYQGYIYTSFQERATFISHGNTARHARKYGDLKL

AQICGTIAADEKRHETAYTKIVEKLFEVDPDYTVLAFAAMMRKKVTMPAHLMYDGQDDNL

FEHFSAVAQRLGVYTAIDYADILDFLVQRWNVANLTGLSGEGRRAQDFLCSLGPRFRKLE

ERAQGRAKQLPVVPFSWIHGRQVQL

>TaFAB2.30

MAYMPSCGTFHAPLVSSCLRRKFTVVATASKAKVGTPEKAPTRVRFAQPFPPQKKEVFDS

LERWAEDNILVLLKPVEKSWQPQDYLPDPSSDGFYDEVKELRERAKEIPDDYLVCLVGDM

VTEEALPTYQTMLNILDGGVGDDTGTSPASWAVWTRAWTAEENRHGDLMNKYMYLAGRVD

MRQIEKTIQYLLGAGMDPKTEGNPYQGYIYTSFQERATFISHGNTARHARKYGDLKLAQI

CGTIAADEKRHETAYTKIVEKLFEVDPDYTVLAFAAMMRKKVTMPAHLMYDGQDDNLFEH

FSAVAQRLGIYTAMDYADILDFLVQRWNVANLTGLSGEGRRAQDFLCSLGPRFRKLEERA

QGRAKQLPVVPFSWIHGRQVQL

>TaFAB2.21

MNHTISVCKHPSNRKRPTMHLMASLPSRGASQAPLWSSCPRSKITVMAMASTAKDGVPGK

AFSPRKAPHRDRVAHSLPPEKREIFDSLNSWAEDNILVLLKPVERSWQPQDYLPDPSSDR

FYDEVKELRERAEEIPDEYLVCLVGDMVTEEALPTYQKMLNILDGGVRDETGSSPTSWAV

WTRAWTAEENRHGDLMNKYIYLTGRADMRQVEKTIQYLVGAGMDPKTEANPYEFFIYTSF

QERATFISHGNTARHARKYGDQKLAQICGTIAADERRHELAYTKIVEKLFEVDPDYTVLA

FASIMKKKITMPAHLMYDGQEDNLFEHFSAVAQRLGVYTAMDYADILEFLVQRWNVAGLT

GLSGEGRRAQDYLCSLGPRFRKLVERAQGSGKQLPVVPFSWIYGRQVQL

>TaFAB2.34

MATAATAVKMSPRSTGYPSSCKPSNTSCYCKPPAAAPMSTLRCRSAVSSRGGPTAGRREE

EEEWRRYLAPERLEVLAQLEPWAEANMLPLLKPVDEAWQPADMLPDAAALGADGFQAACI

ELRARAEGVPDPHLVCLVGNMVTEEALPTYQSMSNRFEGTRDATGADGTAWARWIRGWSA

EENRHGDVLSRYMYLSGRLDMRQVERTVHRLIASGMAMHAPASPYHGFIYVAFQERATSI

SHGNTARQVRAHGDAALARICGAIASDEKRHEAAYTRVVAKLFEVDPDAAVRAMEYMMRR

RITMPAALMDDGRDADLFAHYAAAAQQAGVYTASDYRGILEHLIRQWRVEELSTGLSGEG

RRARDYVCALPEKIRRMEERAHDRVRKEPTPVPFSWIFDRPVSVVLH

>TaFAB2.29

MYLMASLPSHGAFHTPLWYSCSRSTTMVMATASKAKDGVPGKAFSPRKASHRDRVAHSLP

PEKREVFDSLNSWAEDNILVLLKPVERSWQPQDYLPEPSSDRFYDEVKELRERAEEIPDD

YLVCLVGDMVTEEALPTYQKMLNILDGGVRDETGSSQTSWAVWTRAWTAEENRHGDLMNK

YIYLTGRADMRQVEKTIQYLVGAGMDPKTEANPYEFFIYTSFQERATFISHGNTARHARK

YGDLKLAQISGTIAADERRHELAYTKIVEKLFEVDPNYTVLAFASIMKKKITMPAHLMYD

GQENNLFEHFSAVAQRLGVYTAMDYADILEFLVQRWNVAGLTGLSGEGRRAQDYLCSLGP

RFGKLLERAQGSQKKLPVVPFSWIYGRQVQL

>TaFAB2.32

MYLMASLPSHGAFHAPLWYSCSRSKTMVMATASKAKDGVPGKAFSPRKASHRDRVAHSLP

PEKREIFDSLNSWAEDNILVLLKPVERSWQPQDYLPDPSLDRFYDEVKELRERAEEIPDD

YLVCLVGDMVTEEALPTYQKMLNILDGGVRDETGSSPTSWAVWTRAWTAEENRHGDLMNK

YIYLTGRADMRQVEKTIQYLVGAGIDPKTEANPYEFFIYTSFQERATFISHGNTARHARK

YGDLKLAQICGTIAADERRHELAYTKIVEKLFVVDPDYTVLAFASIMKKKITMPAHLMYD

GQEDNLFEHFSAVAQRLGVYTAMDYADILEFLVKRWNVAGLTGLSGEGRRAQDYLCSLGP

RFRKLVERAQGSGKQLPVVPFSWIYGRKVQL

>TaFAB2.33

MATAAAVKMSPRSTGYPPSCKPSNTGCYCKPPAAAPMSTLRCRSAVSSRGGPTAGRREEE

EEWRRYLAPERLEVLARLEPWAEANMLPLLKPADEAWQPADMLPDAAALGADGFHAACLE

LRARAEGVPDAQLVCLVGNMVTEEALPTYQSMSNRFEGTRDTTGADGTAWARWVRGWSAE

ENRHGDVLSRYMYLSGRLDMRQVERTVHRLIASGMAMHAPASPYHGFIYVAFQERATAIS

HGNTARQVRAHGDAALARICGAIAADEKRHEAAYTRVVAKLFEVDPDAAVRAMAYMMRRR

ITMPAALMDDGRDADLFAHYAAAAQQAGVYTASDYRGILEHLIRQWRVEELSAGLSGEGR

RARDYVCALPEKIRRMEERAHDRVRKEPTPVPFIWIFDRPVSVVLH

>TaFAB2.9

MAASWLLKHPCPPERPWTSTRNATGLQLITITYRRTTCTGRSAAVAVKHQEEGTDDEWLA

YLEPAKLEVFDQLEPWAEANVVPLLKPAEVAWQPTDLLPDPASLGADGFHAACCDIRERA

AGLPDAHLVCLVGNMVTEEALPSYQSMANRFEAVHDLTGSSGTAWARWTRGWSAEENRHG

DVLNRYLYLSGRVDMRQVERTIHNLIRSGMVLNAARSPYHGFIYVAFQERATFISHGNTA

RRAKEHGDVALARICGAIAADEKRHELAYTRIVAKLFEIDPDGAVRALAYMMRRRIVMPA

SLMTDGRDDDLFAHYGAVAQQAGIYTASDYRGILEHLIKQWGVEELVAAGLSDEGRRARD

YVCALPQKIRRLEEKAHERRRHKAQPTTPIPFSWIYDRPVNITVA

>TaFAB2.19

MAKLGLAVGTSARFSCFFSNKSDAGSGRATRLGWILFPANPRCSSLATRWTAAAAAAVEA

PPRSVDMGCAPVPREQVEIVQSLNGWVAENMLPLLNPVESSWQPHDFLPCSVAAPGASEE

EALSAFTEGVAALRMGAAGVPDEILVCLVGNMVTEEALPSYQSMGNRTEGTADDTGASSL

PWAQWIRGWTAEENRHGDLLNRYLYLSGRVDMRQVETTVHHLLRNGMEMLVPKSPYHSVI

YGAFQERATFVSHVHTARLAGQHGDQALAKICGVIAADEKRHEAGYTRVCAKLFEVDPDG

MVRALAHVMRGKVTMPGLLMSDGRDADGSLFERFSAVAQRAGVYTARDYGDLVEHFVRRW

RVAELAGLSGEGRRAQEYVCGLPPKIRRMEELAHQRAARSELRPARFSWIFDRHVMVG

>TaFAB2.1

MAASWLLKHPCPPARPWTRTRNATGLRLQQVTTITYRRTTCTGRSAAVAVKHEEEGADEE

WLAYLDPAKLEVFDQLEPWAEANVVPLLKPAEVAWQPTDLLPDPASLGADGFHAACRDLR

ARAAGLPDAHLVCLVGNMVTEEALPSYQSMANRFQAVHDLTGSSGTAWARWTRGWSAEEN

RHGDVLNRYLYLSGRVDMRQVERTIHNLIRSGMVLNAARSPYHGFIYVAFQERATFISHG

NTARRAKEHGDVALARICGAIAADEKRHELAYTRIVAKLFEIDPDGAVRALAYMMRRRIV

MPASLMTDGRDDDLFAHYGAVAHQVGIYTASDYRGILEHLIKQWGVEELVAAGLSDEGRR

ARDYVCALPQNIRRLEEKAHERSRHKAHPMTSIPFSWIFDRPVSITVA

>TaFAB2.14

MAKLGLAVGTSTKFSCFFFSDKSNAGSGRATRLGWILCPANPRCRLARRWTAAAAVEAPP

RSIDMGCAPVPREQAEIVQSLNGWVAENMLPLLNPVESSWQPHDFMPCSAATPGASEEEA

LSAFMDGVAALRAGAARVPDEVLVCLVGNMVTEEALPSYQSMGNRTEGIADDTGASSLPW

AQWIRGWTAEENRHGDLLNRYLYLSGRVDMRQVETTVHHLLRNGMEMLVPKSPYHSVIYG

AFQERATFVSHVHTARLAGQHGDQALAKICGVIAADEKRHEAGYTRVCAKLFELDPDGMV

RALAHVMRGKVTMPGLLMSDGRDAASGENSLFERFSAVAQSAGVYTARDYGDLVEHFVRR

WRVAELAGLSGEGRRAQEYVCGLPPKIRRMEELAHQRAARSELRPARFSWIFDRHVTVG

>TaFAB2.16

MAKLGLAVGTSAKLCFFSNKSDAGSGRATRLGWILFPANHRCSLATRWTAAAAEAVEAPP

RSIDMGHAPVAREQVEIVQSLNGWVAENMLPLLNPVESSWQPHDFLPCSAAAPGASEEEA

LSAFTEGVAALRMGAAGVPDDILVCLVGNMVTEEALPSYQSMGNRTEGTADDTGASSLPW

AQWIRGWTAEENRHGDLLNRYLYLSGRVDMRQVETTVHHLLRNGMEMLVPKSPYHSVIYG

AFQERATFVSHVHTARLAGQHGDQALAKICGVIAADEKRHEAGYTRVCAKLFEVDPDGMV

RALAHVMRGKVTMPGLLMSDGRDAASGENSLFERFSTVAQSAGVYTARDYGDLVEHFVRR

WRVAELAGLSGEGRRAQEYVCGLPPKIRRMEELANQRAARSELRPARFSWIFDRHVMVG

>TaFAB2.6

MATSWLLRHPCPLARPWTRTRNDIGLQVTGITYCYWRCSRASAGGRIMADMSMNTTNCTA

EPALLPQADAADPPPRVSKRSPRTGRGAAVAARHEEEGTDDEWLMYLEPAKLEVFDHLEP

WAEANVVPLLKPAEVAWQPTDLLPDLASLGADGFHAACFDISARAAGLPDAHLVCLVGNM

VTEEALPTYQSIPNRFEAVRDLTGSSGTAWARWIRGWSAEENRHGDVLNKYLFLSGRVDM

RQVERTIHNLIQSGMVMNAARSPYHGFIYVAFQERATSISHGNTARRAKEYGDVALARIC

GAIAADEKRHELAYTRIVGKLFEIDPDGAVRALAYMMRRRIVMPASLMTDGRDGDLFAHY

AAVAQQAGIYTASDYRSILEHLIKQWGVEELAAAELSDDGRRARDYVCALPQKIRRLEEK

AHERSGQKAQPATSAPFSWIFDKPVNNTMG

>TaFAB2.10

MATSWLLRHPCPLARPWTRTRNDIGLHVTSIAYCYWRCTRTSGGGRIMADMSMNTTNCTA

EPALLPQADAAELAGGADHPPRLSKRSARTGRGAAVAARHEEEGTDDEWLMYLEPAKLQV

FDHLEPWAEANVVPLLKPAEVAWQPTDLLPDLASLGTDGFHAACSDITARAAGLPDAHLV

CLVGNMVTEEALPTYQSIPNRFEAVRDLTGSSGTAWARWIRGWSAEENRHGDVLNRYLFL

SGRVDMRQVERTIHNLIQSGMVMNAARSPYHGFIYVAFQERATSISHGNTARRAKEYGDV

ALARICGAIAADEKRHELAYTRIVGKLFEIDPDGAVRALAYMMRRRIVMPASLMTDGRDG

DLFAHYAAVAQQAGIYTASDYRSILEHLIKQWGVKELAAAELSDDGRRARDYVCALPQKI

RRLEEKAHERSGQKTQPATSAPFSWIFDKPVNNTMG

>TaFAB2.3

MATSQSSWLLRHACPLAMPWTRTGNNIGLQVTNITYWRCSRANGGRIMAGMTMSTANCLA

QPAQLQGEAVQVSKRSTRTGRGAAVAARHEEEGTDDEWLMYLEPAKLEVFDHLEPWAEAN

VVPLLKPAEVAWQPTDLLPDLASLGADGFHAACCDIRARAAGLPDAHLVCLVGNMVTEEA

LPTYQSIPNRFEAVRDLTGSSGTAWARWIRGWSAEENRHGDVLNKYLFLSGRVDMRQVER

TIHNLIQSGMVMNAARSPYHGFIYVAFQERATSISHGNTARRAKEHGDLSLARICGAIAA

DEKRHELAYTRIVGKLFEIDPDGAVRALAYMMRRRIVMPASLMTDGRDSDLFSQYGAVAQ

QAGIYTASDYRSILEHLINQWGVEELVATGLSDEGRRARDYVCALPRKIRRLEQKAHERN

DKKARPTASIPFSWIFDRPVNITMA

>TaFAB2.2

MATSWLLRHPCPLARPWTRTRNDIGLHVTSITYCYWRCTKASGGGRIMADMSMHTTSCKA

EPALLPQGDAAELAGGADPPPRASKRSARTGRAATVAARHDEEGTDDEWLMYLEPAKLEV

FDHLEPWAEANVVPLLKPAQVAWQPTDLLPDLASLGADGFHAACSDISARAAGLPDAHLV

CLVGNMVTEEALPTYQSIPNRFEAVRDLTGSSGTAWARWIRGWSAEENRHGDVLNRYLFL

SGRVDMRQVERTIHNLIQSGMVMNAARSPYHGFIYVAFQERATSISHGNTARHAKEHGDL

VLARICGAIAADEKRHELAYTRIVGKLFEIDPDGAVRALAYMMRRRIVMPASLMTDGRDG

DLFAHYAAVAQQTGIYTASDYRSILEHLMKQWGVEELAAAELSDDGRRAREYVCALPHKI

RRLEEKAHERSGQKAQPATSAPFSWIFDKPLNKSMG

>TaFAB2.27

MSMLKCFPHGLAMPAQATWCRGRAAARAGRWACKVTSTANFEDTVTGMAAQEQAEAEVVR

SLGLSGWVEEQLLPLLTPVDDAWQPSDLLPCFSPSAAGLSAEQQPSMMMTTEELQAQASG

VPDDVLVCLVGNMVTEEALPTYMCMGNRVPGYRDDTGCSDLPWARWLRGWMAEENRHGDL

LNRYLYLSGRVDMRQVERTVHHLLRNGMQMLRPSSPYHNAIYGSFQERATFISHAHTAKQ

AARHGDRCLAKICGVVAADEKRHETAYTKVAAKLFELDPDGMVQALAAVLRDKITMPGQL

MTDGREADLFEHFSAVAQRTGVYTARDYGDMVEHFVRRWKVADLAGGQLSGEGRRAQEYV

CGLPRKIRRVEELAHDRAIKAAKEPEFARFSWVFDRPVCIRA

>TaFAB2.24

MSMLKPFPHGLAMPAQAHPTWCRSRAAARAGRWACKVTATANFEGTVMGMASQEQAEAEV

VRGLSLSGWVEEQLLPLLTPVDDAWQPSDLLPCFSLSAAGLSDEQQPSMMMTTEELQAQA

SGVPDDVLVCLVGNMVTEEALPTYMCMGNRVPGYRDDTGCSDLPWARWIRGWTAEENRHG

DLLNGYLYLSGRVDMRQIERTVHHLLRNGMQMLRPSSPYHNAVYGSFQERATFISHAHTA

KQAARHGDRCLAKICGVVAADEKRHETAYTKVAAKIFELDPDGMVRALAAVLRDKITMPG

QLMTDGRDADLFEHFSAVAQRTGVYTARDYGDMVEHFVRRWKVADLGGGQLSSEGRRAQE

YVCGLPRKIRRVEELAHDRAIKAAKEAEFARFSWVFDRPVCIRA

>TaFAB2.28

MSMLMSFPHGLAMPAQAHPSWCRSRAAARAGRWACKSTATAHFDNAVTGMRPQEQAEAEV

ARTLNLSGWVEKQVLPLLTPVEDAWQPSDLLPCFSLTSAGRSAEQQPPPSMMMMTTEELQ

DQASGVPDDVLVCLVGNMVTEEALPTYMCVGNRVAGCSDDTGCSDLPWARWIRGWTAEEN

RHGDLLNRYLYLSGRVDMRQVERTVHHLLRNGMQMLRPSSPYHNIVYGSFQERATFISHT

HTAKHAARHGDRCLAKICGVVAADEKRHETAYTEAAAKLFELDPDGMVRALAAVLRDKIT

MPGQLMTDGRDADLFEHFSAVAQRTGVYTARDYGDMVEHFVRRWRVADLAGGQLSGEGRR

AQEYVCGLPRKIRRVEELAHDRAIKAAKEPEFARFSWVFDRSVCIRA

>TaFAB2.23

MSMLMPFPHGLAMPAQAHPSWCRSRAATRAGGWACKSTATARFDNTVTEMRAQEQAEAEV

ARTLNLSGWVEEQLLPLLTPVEDAWQPSDLLPCFSLSAAGLSAEQQPSMMMTREELQDQA

SSVPDDVLVCLVGNMVTEEALPTYMCVGNRVVGCNDDTGCSDLPWARWIRGWTAEENRHG

DLLNRYLYLSGRVDMRQIERTVHHLLRNGMQMLRPSSPYHNAVYGSFQERATFISHAHTA

KQAARHGDRCLAKICGVVAADEKRHETAYTKAAAKFFELDPDGMVRALAAVLRDKITMPG

QFMTDGRDADLFEHFSAVAQRTGVYTARDYGDMVEHFVRRWKVADLGGRQLSGEGRRAQE

YVCGLPRKIRRVEELAHDRAIKAAKDPEFARFSWVFDRPVCIRA

>TaFAB2.31

MSMLMSFPHGLAMPAQAHPSWCRSRVATRAGRWACKPTATAHFDNTVTGTRAQEQAEAEV

ARTLNLSGWVEKQLLPLLTPVEDAWQPSDLLPCFSLTSAGRSAEQQQPPSMMMMTTEELQ

AQASGVPDDVVVCLVGNMVTEEALPTYMCVGNRVVGCSDDTGCSDLPWARWIRGWTAEEN

RHGDLLNRYLYLSGRVDMRQVERTVHHLLRNGMQMLRPSSPYHNIVYGSFQERATFISHT

HTAKHAARHGDRCLAKICGVVAADEKRHETAYTKAAAKFFELDPDGMVRALAAVLRDKIT

MPGQFMTDGRDADLFEHFSAVAQRTGVYTARDYGDMVEHFVRRWKVADLGGGQLSVEGRR

AQEYVCGLPRKIRRVEELAHDRAIKAAKDPEFARFSWVFDRSVCIRA

>TaFAB2.11

MATSQSSWLLRHLCPLAMPWTRTGNNIGLKVTNITYWRCSRANNGGTMMEDMSMSTANCL

AQPAQLQGEAVEVAGVSKRSTRTGRCAAVAVRHEEEGTDDEWLMYLEPAKLEVFDHLEPW

AEANVLPLLKPAEVAWQPTDLLPDLASLGADGFRAACSDISARAAGLPDAHLVCLVGNMV

TEEALPTYQSIPNRFEAVRDLTGSSCTAWARWIRGWSAEENRHGDVLNRYLFLSGRIDMR

QVERTIHNLIHSGMVMNAARSPYHGFIYVAFQERATSISHGNTARRANEHGDVALARICG

AIAADEKRHELAYTRIVGKLFEIDPDGSVRALAYMMRRRIIMPASLVTDGCDGDLFAHYA

AVAQQAGIYTASDYRSILEHLIKQWGVEELVAAELSDDGRRARDYVCALPKKIRRLEEKA

HERNGKKAQPMTSVSFSWIFDRPVNIGMA

>TaFAB2.25

MSMLKSFPHGIAMPAQAHPSWFRNRVATRAGTRACKFTATAHFEDTVTGMPVPEQAEAEV

ARSLNLGGWVQEQMLPLLTSVEDAWQPSDLLPCFSLSSAGSVAEEQQPSMTMMVEELQAR

ASGVPDDVLVCLVGNMVTEEALPTYMCMSNRVVGNRDDTGCSEPPWARWLRGWTAEENRH

GDLLNRYLYLSGRVDMRRVERTVHHLLRNGMQMLRPSSPYHNVGYTSFQERDTFISHSHT

ARHAMRHGDRCLAKICGVVAADEKRHEAAYTKAAAKLFELDPDGMVRAVAAVLRDKITMP

GQLMTDGRDADLFAKFSAVAQRTGVYTARDYGDMVEHFVRRWKVADLAGGQLSGEGRRAQ

EYVCGLPRKIRRVEELAHDRAIKAAKEPEFARFSWVFDRSVCITGRA

>TaFAB2.7

MATSQSSWLLRHPCPVSMPWTWNNIGLKVTNITYWRCSRANNGGRIMADMSISTANCLAQ

PAQLQGETPTGRGSAVAARHEEEGTDDEWLMYLEPAKLEPFDHLEPWAEANVVPLLKPAQ

VAWQPTDLLPDLASLGADGFHAACSDIAARAADLPDAHLVCLVGNMVTEEALPTYQSIPN

RFEAVRDLTGSSATAWARWIRGWSAEENRHGDVLNRYLFLSGRVDMRQVERTIHNLIHPS

RQGARRRGAIAADEKRHELAYTRIVGKLFEIDPDGAVRALAYMMRRRIVMPASLVTDGRD

GDLFGHYAAVAQQAGIYTASDYRSILEHLIKQWGVEELAAAELSYDGRRARDYVCSLPKK

IYRLEEKAHTRNSKKAQRMTSVSFSWIFDRPINISVA

>TaFAD7.1

MARLALSDCRGLTPLRARSRGGAIALPSPPHLAAGPRRPAAAAIHRDWALRVAAPTRLAS

VFEEDKRSLGGAEEAGSSSAGFNPGAPPPFGLAEIRAAIPKHCWVKDPWRSMSYVLRDVL

VVLGLAAAAARVDSWLVWPLYWAAQGTMFWALFVLGHDCGHGSFSSNPKLNSVVGHILHS

SILVPYNGWRISHRTHHQNHGHVEKDESWHPLPQRLYNSLDNMTKKLRFSMPFPMLAFPL

YLFARSPGKEGSHFNPNSDLFQPNEKKDVLTSTASWLAMIGVLAGLTFVMGPLKMLKLYA

VPYVIFVMWLDFVTYLHHHGHEDKVPWYRGKEWSYLRGGLTTLDRDYGLINNIHHDIGTH

VIHHLFPQIPHYNLVEATEAAKPVLGKYYKEPEKSAPLPFHLLQVLSRSLKEDHYVSDTG

DIVYYQSESETSTSGQSSD

>TaFAD7.3

MTRLALSDCRGLTPLRARSRGSAIALPSPPHLAAGPRRPAAAAIHRDWALRVAAPTRLAS

VFEEDKRGLGGAEEAGGSSAGFNPGAPPPFGLAEIRAAIPKHCWVKDPWRSMSYVLRDVL

VVLGLAAAAARADSWLVWPLYWAAQGTMFWALFVLGHDCGHGSFSSNPKLNSVVGHILHS

SILVPYNGWRISHRTHHQNHGHVEKDESWHPLPQRLYNSLDNMTKKLRFSMPFPMLAFPL

YLFARSPGKEGSHFNPNSDLFQPNEKKDVLTSTASWLAMIGVLAGLTFVMGPLKMLKLYA

IPYVIFVMWLDFVTYLHHHGHEDKVPWYRGKEWSYLRGGLTTLDRDYGLINNIHHDIGTH

VIHHLFPQIPHYHLVEATEAAKPVLGKYYKEPEKSAPLPFHLLQVLSRSLKEDHYVSDTG

DIVYYQSESETSTCAQSSD

>TaFAD7.2

MARLALSDCRGLTPLRARSRGSAIALPSPPHLAAGPRRAAPAAIHRDWALRVAAPTRLAS

VFEEDRRGLGGAEEAGSSSAGFNPGAPPPFGLAEIRAAIPKHCWVKDPWRSMSYVLRDVL

VVLGLAAAAARADSWLVWPLYWAAQGTMFWALFVLGHDCGHGSFSSNPKLNSVVGHILHS

SILVPYNGWRISHRTHHQNHGHVEKDESWHPLPQRLYNSLDNMTKKLRFSMPFPMLAFPL

YLFARSPGKEGSHFNPNSDLFQPNENKDVLTSTASWLAMIGVLAGLSFVMGPLKMLKLYA

VPYVIFVMWLDFVTYLHHHGHEDKVPWYRGKEWSYLRGGLTTLDRDYGLINNIHHDIGTH

VIHHLFPQIPHYHLVEATEAAKPVLGKYYKEPEKSAPLPFHLLQVLSRSLKEDHYVSDTG

DIVYYQSESETSTSAQSSD

>TaFAD3.6

MAPAMRPEQEASCKATEDHRSEFDAAKPPPFRIGDVRAAVPAHCWRKSPLRSLSYVARDV

VVVAALAAAAWRADSWALWPLYWAVQGTMFWALFVLGHDCGHGSFSDSGTLNSVVGHLLH

TFILVPYNGWRISHRTHHQNHGHIEKDESWHPITEKVYQKLEPRTKTLRFSVPFPLLAFP

VYLWYRSPGKEGSHFNPSSDLFTPKERRDVIISTTCWFTMIALLIGMACVFGLVPVLKLY

GVPYIVNVMWLDLVTYLHHHGHQDLPWYRGEEWSYLRGGLTTVDRDYGWINNIHHDIGTH

VIHHLFPQIPHYHLVEATKAARPVLGRYYREPEKSGPLPMYLITVLLKSLRVDHFVSDVG

DVVFYQTDPSLSGDKWTGTDKQK

>TaFAD3.5

MAPAMRPEQEASCKATEDHRSEFDAAKPPPFRIGDVRAAVPAHCWRKSPLRSLSYVARDV

AVVAALAAAAWRADSWALWPLYWAVQGTMFWALFVLGHDCGHGSFSDSGTLNSVVGHLLH

TFILVPYNGWRISHRTHHQNHGHIEKDESWHPITEKVYQKLEPRTKTLRFSVPFPLLAFP

VYLWYRSPGKEGSHFNPSSDLFTPKERRDVIISTTCWFTMIALLIGMACVFGLVPVLKLY

GVPYIVNVMWLDLVTYLHHHGHQDLPWYRGEEWSYLRGGLTTVDRDYGWINNIHHDIGTH

VIHHLFPQIPHYHLVEATKAARPVLGRYYREPEKSGPLPMHLITVLLKSLRVDHFVSDVG

DVVFYQTDPSLSGDKWTGTDKQK

>TaFAD3.4

MAPAMRPEQEASCKATEDHRSEFDAAKPPPFRIGDVRAAVPAHCWRKSPLRSLSYVARDV

AVVAALAAAAWRADSWALWPLYWAVQGTMFWALFVLGHDCGHGSFSDSGTLNSVVGHLLH

TFILVPYNGWRISHRTHHQNHGHIEKDESWHPITEKVYQKLEPRTKTLRFSVPFPLLAFP

VYLWYRSPGKEGSHFNPSSDLFTPKERRDVIISTTCWFTMIALLIGMACVFGLVPVLKLY

GVPYIVNVMWLDLVTYLHHHGHQDLPWYRGEEWSYLRGGLTTVDRDYGWINNIHHDIGTH

VIHHLFPQIPHYHLVEATKAARPVLGRYYREPEKSGPLPVHLITVLLKSLRVDHFVSDVG

DVVFYQTDPSLSGDKWTGADKQK

>TaSLD.1

MARTGLADATAPEADAMPAASKDAADVRMISTKELQAHAAADDLWISISGDVYDVTPWLR

HHPGGEVPLITLAGQDATDAFMAYHPPSVRPLLRRFFVGRLSDYTVPPASADFRRLLAQL

SSAGLFERVGHTPKFLLVAMSVLFCIALYCVLACSSTGAHMFAGGLIGFIWIQSGWIGHD

SGHHQITKHPALNRLLQVVSGNCLTGLGIAWWKFNHNTHHISCNSLDHDPDLQHLPLFAV

STKLFNNLWSVCYERTLAFDAISKFFVSYQHWTFYPVMGFARINLLVQSIVFLITQKKVR

QRWLEIAGVAAFWVWYPLLVSCLPNWWERVAFVLASFVITGIQHVQFCLNHFSSAVYVGP

PKGNDWFERQTAGTLDIKCSPWMDWFHGGLQFQVEHHLFPRLPRCHYRMVAPIVRDLCKK

HGLSYGAATFWEANVMTWKTLRAAALQAREATTGAVPKNLVWEALNTHG

>TaFAD3.2

MGAVARRAPEQEQSCKATEDFDAAKPPPFRIGDVRAAVPAHCWHKSPLRSLSYVARDVAV

VAALAVAAWWLNSWAVWPLYWAAQGTMFWALFVLGHDCGHGSFSDSGTLNSVVGHLLHTF

ILVPYNGWRISHRTHHQNHGHIDKDESWHPITENVYKEMEPSTKKLRFSLPYPLLAFPVY

LWYRSPGKNGSHFNPSSDLFSPRERLDVIVSTTCWFTMIALLIAMACAFGPVPVLKLYGV

PYAVFVMWLDLVTYLHHHGHQDLPWYRGEEWSYLRGGLTTVDRDYGWINNIHHDIGTHVI

HHLFPQIPHYHLVEATKAARPVLGRYYREPEKSGPLPLHLFHVLLRSLRVDHFVSDVGDV

VFYQTDPSLNGDNWAKDGKHK

>TaFAD3.3

MGAAARRAPEQEQSCKATEDFDAAKPPPFRIGDVRAAVPAHCWRKSPLRSLSYVARDVAV

VAALAGAAWWLNSWAVWPLYWAAQGTMFWALFVLGHDCGHGSFSDSGTLNSVVGHLLHTF

ILVPYNGWRISHRTHHQNHGHIDKDESWHPITENLYKEMEPSTKKLRFSLPYPLLAFPVY

LWYRSPGKNGSHFNPSSDLFSPKERLDVIVSTTCWFTMIALLIAMACVFGLVPVLKLYGV

PYAVFVMWLDLVTYLHHHGHQDLPWYRGEEWSYLRGGLTTVDRDYGWINNIHHDIGTHVI

HHLFPQIPHYHLVEATKAARPVLGRYYREPEKSGPLPLHLFHVLLRSLRVDHFVSDVGDV

VFYQTDPSLNGDNWTKNGKHK

>TaSLD.2

MARTGLADATAPEADAMPAASKDAADVRMISTKELQAHAAADDLWISISGDVYDVTPWLR

HHPGGEVPLITLAGQDATDAFMAYHPPSVRPLLRRFFVGRLTDYTVPPASADFRRLLAQL

SSAGLFERVGHTPKFLLVAMSVLFCIALYCVLACSSTGAHMFAGGLIGFIWIQSGWIGHD

SGHHQITRHPALNRLLQVVSGNCLTGLGIAWWKFNHNTHHISCNSLDHDPDLQHLPLFAV

STKLFNNLWSVCYERTLAFDAISKFFVSYQHWTFYPVMGFARINLLVQSIVFLITQKKVR

QRWLEIAGVAAFWVWYPLLVSCLPNWWERVAFVLASFVITGIQHVQFCLNHFSSAVYVGP

PKGNDWFERQTAGTLDIKCSPWMDWFHGGLQFQVEHHLFPRLPRCHYRMVAPIVRDLCKK

HGLSYGAATFWEANVMTWKTLRAAALQAREATTGAAPKNLVWEALNTHG

>TaSLD.3

MARTGLADATAPEADAMPAASKDAADVRMISTKELQAHAAADDLWISISGDVYDVTPWLR

HHPGGEVPLITLAGQDATDAFMAYHPPSVRPLLRRFFVGRLSDYTVPPASADFRRLLAQL

SSAGLFERVGHTPKFLLVAMSVLFCIALYCVLACSSTGAHMFAGGLIGFIWIQSGWIGHD

SGHHQITRHPALNRLLQVVSGNCLTGLGIAWWKFNHNTHHISCNSLDHDPDLQHLPLFAV

STKLFNNLWSVCYERTLAFDAISKFFVSYQHWTFYPVMGFARINLLVQSIVFLITQKKVR

QRWLEIAGVAAFWVWYPLLVSCLPNWWERVAFVLASFVITGIQHVQFCLNHFSSAVYVGP

PKGNDWFERQTAGTLDIKCSPWMDWFHGGLQFQVEHHLFPRLPRCHYRMVAPIVRDLCKK

HGLSYGAATFWEANVMTWKTLRAAALQAREATTGAAPKNLVWEALNTHG

>TaFAD3.1

MGAAARRAAEQEQSCKATEDFDAAKPPPFRIGDVRAAVPAHCWRKSPLRSLSYVARDVAV

VAALAVAAWWLNSWAVWPLYWAAQGTMFWALFVLGHDCGHGSFSDSGTLNSVVGHLLHTF

ILVPYNGWRISHRTHHQNHGHIDRDESWHPITENVYKEMEPSTKKLRFSLPYPLLAFPVY

LWYRSPGKNGSHFNPSSDLFSPKERLDVIVSTTCWFTMIALLIAMACVFGPVPVLKLYGV

PYAVFVMWLDLVTYLHHHGHQDLPWYRGEEWSYLRGGLTTVDRDYGWINNIHHDIGTHVI

HHLFPQIPHYHLVEATKAARPVLGRYYREPEKSGPLPLHLFHVLLGSLRVDHFVSDVGDV

VFYQTDPSLNGDNWTKNGKHK

>TaFAD8.3

MARLLLPQCCCGLTPLPLPRRAVALPPPALFPSSSGAAASRRALSLRVAVAAPARLATAE

DDGSGSRAAGAQGGDEGPADGFDPGAPPPFGLADIRAAIPKHCWVKDPWRSMGYVVRDVV

VVLALAAAAARLDSWLAWPVYWAAQGTMFWALFVLGHDCGHGSFSNNAKLNSVVGHILHS

SILVPYNGWRISHRTHHQNHGHVENDESWHPLPEKLYRSLDSSTRKLRFALPFPMLAYPF

YLWSRSPGKSGSHFHPSSDLFQPNEKKDIVTSTTCWLAMAGLLAGLTVVMGPLQILKLYA

VPYWIFVMWLDFVTYLHHHGHNDKLPWYRGKAWSYLRGGLTTLDRDYGWLNKIHHDIGTH

VIHHLFPQIPHYHLVEATEAAKPVLGKYYREPDKSGPFPFHLFGALARSMKSDHYVSDTG

DIIYYQTDPKLAAGAHTSD

>TaFAD8.2

MARLLLPQCCCGLTPLPLPRRAVALPPPPSLLPSSGVAASRRALSLRVAVAAPARLVTAE

DDGSGSSSRAQGGDEGPSADGFDPGAPPPFGLADIRAAIPKHCWVKDPWRSMGYVVRDVV

VVLALAAAAARLDSWLAWPVYWAAQGTMFWALFVLGHDCGHGSFSNNAKLNSVVGHILHS

SILVPYNGWRISHRTHHQNHGHVENDESWHPLPEKLYRSLDSSTRKLRFALPFPMLAYPF

YLWSRSPGKSGSHFHPSSDLFQPNEKKDILTSTTCWLAMAGLLAGLTVVMGPLQILKLYA

VPYWIFVMWLDFVTYLHHHGHNDKLPWYRGKAWSYLRGGLTTLDRDYGWLNNIHHDIGTH

VIHHLFPQIPHYHLVEATEAAKPVLGKYYREPDKSGPFPFHLFGALARSMKSDHYVSDTG

DIIYYQTDPKLAAGAHTSD

>TaFAD8.1

MARLLLPQCCCGLTPLHLPRRAVALPPPPSLLPSSGVAASRRALSLRVAVAAPARLVTAE

DDGSGSGSSSRASAGQDGGEGPSADGFDPGAPPPFGLADIRAAIPKHCWVKDPWRSMGYV

VRDVVVVLALAAAAARLDSWLAWPVYWAAQGTMFWALFVLGHDCGHGSFSNNAKLNSVVG

HILHSSILVPYNGWRISHRTHHQNHGHVENDESWHPLPEKLYRSLDSATRKLRFALPFPM

LAYPFYLWSRSPGKSGSHFHPSSDLFQPNEKKDILTSTTCWLAMAGLLAGLTVVMGPLQI

LKLYAVPYWIFVMWLDFVTYLHHHGHNDKLPWYRGKAWSYLRGGLTTLDRDYGWLNNIHH

DIGTHVIHHLFPQIPHYHLVEATEAAKPVLGKYYREPDKSGPFPFHLFGALARSMKSDHY

VSDTGDIIYYQTDPKLAGGAQASD

>TaFAD6.2

MFTEPRRCSGRYRAPATSRCNLRRALASPLPGCCVHHNHHASAGPSPIQTISTLLSPGPR

RRLHTLYARPPRRGRMATASGLSAPLQLLSLRRAPAKNLSPRRAAAGALSGSLIVKRFSL

CNGRSHHQFLPLKQRGRLQAAVLPVTPPLLDDEEKRKQMSEDYGFNQIGEQLPDNITLKD

VMDTLPKEVFEIDDVKAWASVLISVTSYAFGLFLISKAPWYLLPVAWAWAGTAVTGFFVI

GHDCAHKSFSRNKLVEDIVGTLAFLPLIYPYEPWRFKHDRHHAKTNMLVEDTAWQPVWQN

EIESSSFLRKAIIFGYGPIRPWMSIAHWLMWHFDLKKFRPNELPRVKISLACVFAFMAIG

WPLIILQSGIAGWFKFWFMPWMVYHFWMSTFTMVHHTAPHIPFKSSKEWNAAQAQLNGTV

HCSYPRWIEILCHDINVHVPHHISPRIPSYNLRAAHDSIKQNWGKYINEASWNWRLMKTI

LTTCHVYDKERYYVSFDELVPEESQPIRFLKKFMPDYA

>TaFAD2.11

MGAGGRMTEKEREKQELLGRTGAGAVFQRSPTDKPPFTLGQIKMAIPPHCFQRSLVKSSS

YLVHDLVIVAALLYAALVWIPALPSMLQLGAWPLYWVAQGCVMFGVWVIAHECGHHAFSD

YPLLDDVVGLVLHSWLLVPYFSWKHSHRRHHSNTGSLERDEVFVPRPKEALPWYTPYIHD

NPVVRVVLIVVQLTLGWYMYLSLNTWGRPYPRFACHFDPYSPIYNDRERAQIFISDIGVL

AVSFAMLKLVSTFGFWWMMRVYGVPLMIVNAWLVLVTYLHHTHPALPHYDSTEWDWLRGA

LATMDRDYGIILNRVFHNITDTHILHHLFSNIPHYHAMEATKAIKPILGEYYQIDRTPLA

KATWREAKECLYIEREDSKGIFWYSNKF

>TaFAD2.2

MRYARFHIWRRDPKNLFLHHHRVCSCRACLDALSCARRAEVHCIMGAGGRMTEKERELLG

RGGASTTFQRSPTDKPPFTLAQIKKAIPPHCFERSVIKSFSYLGHDLVIVAALLYVALVW

IPTLPSVLQLGAWPLYWVVQGCVMTGVWVIAHECGHHAFSDSSLLDDIVGLVLHSWLLVP

YFSWKYSHRRHHSNTGSLERDEVFVPKQKETLAWYAPYIYSNPVGRLGLLVVQLTIGWPM

YLSLNTCGRPYLRFACHFDPYSPIYNDRERAQVSISDVGVLAVSLALSKLASALGFWWVV

RVYGVPLLIVNAWLVLVTYLQHTHPALPHYDSTEWDWLRGALATMDRDYGILNRVFHNIT

DTHVAHHLFSTMPHYHAMEATKAIKPILGEYYQFDPTPVAKATWREAKECIYVRPEDRKG

VFWYSNKFSAP

>TaFAD2.9

MGAGGRMTEKERELLGRGGAGATFQRSPTDKPPFTLAQIKKAIPPHCFERSVIKSFSYLV

HDLVIVATLLYAALVWIPTLPSVLQLGAWPLYWVVQGCVMTGVWVIAHECGHHAFSDSSL

LDDIVGLVLHSWLLVPYFSWKYSHRRHHSNTGSLERDEVFVPKQKEALAWYAPYIYSNPV

GRLGLLAVQLTIGWPMYLSLNTCGRPYPRFACHFDPYSPIYNDRERAQVFISDVGVLAVS

LALFKLASSFGFWWVVRVYAVPLLIVNAWLVLITYLQHTHPALPHYDSTEWDWLRGALAT

MDRDYGILNRVFHNITDTHVAHHLFSNMPHYHAMEATKAIKPILGEYYHFDATPFAKATW

REAKECIYVEPEDRKGVFWYSNKFSAP

>TaFAD2.1

MGAGGRMTEKEREKQEQLGRANGGAAYQRSPTDKPPFTLGQIKKAIPPHCFQRSIIKSFS

YVVHDLVIVAALLYAALVWIPTLPSVLQLGAWPLYWVVQGCVMTGVWVIAHECGHHAFSD

YSLLDDTVGLVLHSWLLVPYFSWKYSHRRHHSNTGSLERDEVFVPKQKEALAWYTPYIYN

NPVGRLVHIVVQLTLGWPLYLALNASGRPYPRFACHFDPYGPIYNDRERAQIFISDVGVL

AVSLALLKLVSSFGFWWVVRVYGVPLLIVNAWLVLITYLQHTHPALPHYDSTEWDWLRGA

LATMDRDYGILNRVFHNITDTHVAHHLFSTMPHYHAMEATKAIKPILGEYYQFDPTPVAK

ATWREAKECIYVEPEDRKGVFWYSNKF

>TaFAD2.5

MPQLWYFRAIYSHHRGCSCRACLDALSCARRAEVHCIMGAGGRMTEKERELFGRGGAGAT

FQRSPTDKPPFTLAQIKKAIPPHCFQRSVIKSFSYLVHDLVIVGALLYVALVWIPTLPSV

LQLGAWPLYWVLQGCVMTGVWVIAHECGHNAFSDSSLLDDIVGLVLHSWLLVPYFSRKYS

HRRHHSNTGSLERDEVFVPKQKEALAWYAPYIYNNPVGRLGLLVVQLTIGWPMYLSLNTC

GRPYPRFACHFDPYSPIYNDRERAQVFISDVGVLAVSLALFKLASAFGFWWVVRVYGVPL

LIVNAWLVLITYLQHTHPALPHYDSTEWDWLRGALATMDRDYGILNRVFHNITDTHVAHH

LFSNMPHYHAMEATKAIKPILGEYYQFDPTPVAKATWREAKECIYVEPEDRTGVFWYSNK

FAAP

>TaFAD2.10

MGAGGRMTEKEREKQELLGRAGAGEIFQRAPTDKPAFTLAQIKKAIPPHCFQRSVIKSFS

YVVYDLVIIASLLYAALVWIPALPAMQQLGAWPLYWAAQGCVMTGVWVLAHECGHHAFSD

YLLLDNILGLVLHSCLLVPYFSWKYSHRRHHANTGSLEHDEVYVPKKKEALPWYTPYIYN

NPVGRLGYIVVQLTLGWPMYLALNTSGRPYPRFVCHYDPYGPMYSELERAQVFISDAGVL

AVSLALLKLASAFGFWWVVRVYGVPLLIVNAWLVVITYLAHTHPALPHYDSTEWDWLRGA

LATMDRDLGVLNRVFHNTTDTHVAHHLFSSIPHYHAMEATKAIKPILGEYYQLESNPLAK

ATWRSAKECIYVQPEDRKGVFWYSNKF

>TaFAD2.8

MGAGGRMTEKEREKQEQLGRANGGAAYQRSPTDKPPFTLGQIKKAIPPHCFQRSIIKSFS

YVVHDLVIVAALLYAALVWIPTLPTVLQLGAWPLYWIVQGCVMTGVWVIAHECGHHAFSD

YSLLDDTVGLVLHSWLLVPYFSWKYSHRRHHSNTGSLERDEVFVPKQKEALAWYTPYIYN

NPIGRLVHIVVQLTLGWPLYLALNASGRPYPRFACHFDPYGPIYNDRERAQIFISDVGVL

AVSLALLKLVSSFGFWWVVRVYGVPLLIVNAWLVLITYLQHTHPALPHYDSTEWDWLRGA

LATMDRDYGILNRVFHNITDTHVAHHLFSTMPHYHAMEATKAIKPILGEYYQFDPTPVAK

ATWREAKECIYVEPEDRKGVFWYSNKF

>TaFAD6.1

MATASGLSAPLQLLSFRRAPAKNLSPRRAAAGALSGSLIVKRFSLCNGRSHHQFLPLKQR

GRLQAAVLPVTPPLLDDEEKRKQMSEDYGFNQIGEQLPDNITLKDVMDTLPKEVFEIDDV

KAWASVLISVTSYAFGLFLISKAPWYLLPVAWAWAGTAVTGFFVIGHDCAHKSFSRNKLV

EDIVGTLAFLPLIYPYEPWRFKHDRHHAKTNMLVEDTAWQPVWQNEIESSSFLRKAIIFG

YGPIRPWMSIAHWLMWHFDLKKFRPNELPRVKISLACVFAFMAIGWPLIILQSGIAGWFK

FWFMPWMVYHFWMSTFTMVHHTAPHIPFKSSKEWNAAQAQLNGTVHCSYPRWIEILCHDI

NVHVPHHISPRIPSYNLRAAHDSIKQNWGKYINEASWNWRLMKTILTTCHVYDKERYYVS

FDELVPEESQPIRFLKKFMPDYA

>TaFAD2.6

MIEYNITSFMPSDHLCFFVVCSIMGAGGRMTEKEREKQEQLGRANGGAAYQRSPTDKPPF

TLGQIKKAIPPHCFQRSIIKSFSYVVHDLVIVAALLYAALVWIPTLPTVLQLGAWPLYWI

VQGCVMTGVWVIAHECGHHAFSDYSLLDDTVGLVLHSWLLVPYFSWKYSHRRHHSNTGSL

ERDEVFVPKQKEALAWYTPYIYNNPVGRLVHIVVQLTLGWPLYLALNASGRPYPRFACHF

DPYGPIYNDRERAQIFISDVGVLAVSLALLKLVSSFGFWWVVRVYGVPLLIVNAWLVLIT

YLQHTHPALPHYDSTEWDWLRGALATMDRDYGILNRVFHNITDTHVAHHLFSTMPHYHAM

EATKAIKPILGEYYQFDPTPVAKATWREAKECIYVEPEDRKGVFWYSNKF

>TaFAD2.7

MGAGGRMAEKEREKQELLGRTGAGTAFQRSPMDKPPFTLGQIKMAIPPHCFQRSLVKSSS

YLVHDLVIVAALLYAALVWIPALPGMLQLGAWPLYWVAQGCIMFGVWVIAHECGHHAFSD

YPLLDDIVGLVLHSWLLVPYFSWKHSHRRHHSNTGSLERDEVFVPRPKEALPWYTPYIHD

NPVVHVVLIVVQLTLGWYMYLPLNTWGRPYPRFACHFDPYSPIYNDRERAQIFISDVGVL

AVSFAMLKLVSTFGFWWVVRVYGVPLMIVNAWLVLVTYLHHTHPALPHYDSTEWDWLRGA

LATMDRDYGILNRVFHNITDTHILHHLFSNIPHYHAMEATKAIKPILGEYYQLDATPLAK

ATWREAKECLYIEREDSKGIFWYSNKF

>TaFAB2.4

MYLMAYMPSCGTFHAPLVSSCLRRKFTVVATASKAKVGTPKKASTQVRFTQPFPPEKKEI

FDSLERWAEENILVLLKPVEKSWQPQDYLPDPSSDGFYDEVKELRERAKEIPDDYLVCLV

GDMVTEEALPTYQTMLNILDGGVGDDTGTSPASWAVWTRAWTAEENRHGDLMNKYMYLTG

RVDMRQIEKTIQYLLGAGMDPKTEGNPYEGYIYTSFQERATFISHGNTARHARKYGDLKL

AQICGTIAADEKRHETAYTKIVEKLFEVDPDYTVLAFAAMMRKKVTMPAHLMYDGQDDNL

FEHFSAVAQRLGVYTAMDYADILDFLVQRWNVANLTGLSGEGRRAQDFLCSLGPRFRKLE

ERAQGRAKQLPVVPFSWIHGRQVQL

>TaFAD2.3

MGAGGRMTEKEREKQELLGRTGVGAAFQRSPTDKPPFTLGQIKMAIPPHCFQRSLVKSSS

YLVHDLVIVAALLYAALVWIPALPSMLQLGAWPLYWVAQGCVMFGVWVIAHECGHHAFSD

YPLLDDIVGLVLHSWLLVPYFSRKHSHRRHHSNTGSLERDEVFVPRPKEALPWYTPYIHD

NPVVRVVLIMVQLTLGWYMYLSLNTWGRPYPRFACHFDPYSPIYNDRERPQIFISDVGVL

AVSFAMLDFVSTFGFWWMMRVYGAPLMIVNAWLVLVTYLHHTHPALPHYDSTEWDWLRGA

LATMDRDYGILNRVFHNITDTHILHHLFSNIPHYHAMEATKAIKPILGEYYQIDRTPLAK

ATWREAKECLYIVREDSKGIFWYSNKF

>TaDES.1

MGATRADADEKEEGVMTTDFFWSYTDEPHASRRREILAKYPQIKVLFGPDPLAFIKIAAV

VSLQLWTATLLRDAGWSKILPVAYFFGSFLNHNLFLAIHELSHNLAFATPSLNRWLGIFA

NLPIGVPMSVTFQKYHLEHHRFQGVDGIDMDIPSQTEAHVVKNTVSKSIWVVLQLFFYAL

RPLFLKPKPPGLWEFTNLMIQVALDAAMVYLYGWKSLAYLILSTFLGGGMHPMAGHFISE

HYVFSPEQETYSYYGPLNLMTWHVGYHNEHHDFPRIPGAKLHKVKEIAPEYYDSLKSYRS

WSQVIYMYVMDQTVGPFSRMKRKAPKKDL

>TaDES.3

MGATRADADEKEEGVMATDFFWSYTDEPHASRRREILAKYPQIKELFGSDPLAFIKIAAV

VSLQLWTATLLRDAGWSKILPVAYFFGSFLNHNLFLAIHELSHNLAFATPSLNRWLGIFA

NLPIGVPMSVTFQKYHLEHHRFQGVDGIDMDIPSQTEAHVVKNTVSKSIWVVLQLFFYAL

RPLFLKPKPPGLWEFTNLTIQVALDAAMVYLYGWKSLAYLILSTFLGGGMHPMAGHFISE

HYVFSPEQETYSYYGPLNLMTWHVGYHNEHHDFPRIPGAKLHKVKEIAPEYYDSLKSYRS

WSQVIYMYVMDQTVGPFSRMKRKAPKKDL

>TaDES.2

MGATRADADEKDADEKEEGVMATDFFWSYTDEPHASRRREILAKYPQIKELFGPDPLAFI

KIAAVVSLQLWTATLLRDAGWSKMLVVAYFFGSFLNHNLFLAIHELSHNLAFATPSLNRW

LGIFANLPIGVPMSVTFQKYHLEHHRFQGVDGIDMDIPSQTEAHVVKNTVSKSIWVVLQL

FFYALRPLFLKPKPPGLWEFTNLTIQVALDAAMVYLYGWKSLAYLILSTFLGGGMHPMAG

HFISEHYVFSPEQETYSYYGPLNLMTWHVGYHNEHHDFPRIPGTKLHKVKEIAPEYYNSL

KSYRSWSQVIYMYVMDQTVGPFSRMKRKAPKKDS

>TaFAD2.4

MGAGGRMTEKEREKQELLGRAGAGEISQRAPTDKPAFTLAQIRKAIPPHCFQRSVIKSFS

YVVYDLAIIASLLYAALVWIPALPTMQQLGAWPLYWAAQGCVMTGVWVLAHECGHHAFSD

YLLLDNIVGLVLHSCLLVPYFSWKYSHRRHHANTGSLEHDEVYVPKKEALPWYTPYIYNN

PVGRLGYIVVQLTLGWPMYLALNTSGRPYPRFVCHYDPYGPLYSDLERAQVFISDVGVLA

VSFALLKLASAFGFWWVVRVYGVPLLIVNAWLVVITYLAHTHPALPHYDYDSPPWTVTWA

SSIACSTTPRTRTSRTISSPAYRTTTPWRPPRRSSPSSASTTSSNRTPSPGPHGARPRSA

STSSPRIARESFGTATSSSRHHPTK

>TaFAD4.3

MPAMYALTPRCTLPPHRRAPPPCRAASPTPAALARADPDELRSTWPQRAWTLAGSAAVLS

SLSASASLAADSGSYAEPLAAALAAYTVADLATGVYHWLVDNYGDASTPLVGAQIAAFQG

HHRHPSTITRREPCNNLHALARAVALALPAVEAAAAAAHAPAAAHAFAGTFAACVVLSQQ

FHAWAHEKRRRLPPGVEALQAAGVLVSRAQHAAHHRQPYSTNYCILSGMWNGVLDRHKVF

EALEMVVFFRTGVRPRSWDETQAAWMEDTSGSVTAVAVTDSS

>TaFAD4.2

MPAMYALTPRCTLPPHRRAPPPCRAASPTPAALARADPDELRSTWPQRAWTLAGSAAVLS

SLSASASLAADSGSYAEPLAAALAAYTVADLATGVYHWLVDNYGDASTPLVGAQIAAFQG

HHRHPSTITRREPCNNLHALARAVALALPAVEAAAAAAHAPAAAHAFAGTFAACVVLSQQ

FHAWAHEKRRRLPPGVEALQAAGVLVSRAQHAAHHRQPYSTNYCILSGVWNGVLDRHKVF

EALEMVVFFRTGVRPRSWDETQAAWMEDTGSAAAVALTDSS

>TaFAD4.1

MPAMYALTPRCTLPPVHRRAPPPCRAASPTPAALARADPDELRSTWPQRAWTLAGSAAVL

SSLSASASLAADSGSYAEPLAAALAAYTVADLATGVYHWLVDNYGDASTPLVGAQIAAFQ

GHHRHPSTITRREPCNNLHALARAVALALPAVEGAAAAAHAPAAAHAFAGTFAACVVLSQ

QFHAWAHEKRRRLPPGVEALQAAGVLVSRAQHAAHHRQPYSTNYCILSGVWNGVLDRHKV

FEALEMVVFFRTGVRPRSWDETQAAWMEDTSGAAAVTAIAVTDSS

>GmFAD2.3

MGLAKETTMGGRGRVAKVEVQGKKPLSRVPNTKPPFTVGQLKKAIPPHCFQRSLLTSFSY

VVYDLSFAFIFYIATTYFHLLPQPFSLIAWPIYWVLQGCLLTGVWVIAHECGHHAFSKYQ

WVDDVVGLTLHSTLLVPYFSWKISHRRHHSNTGSLDRDEVFVPKPKSKVAWFSKYLNNPL

GRAVSLLVTLTIGWPMYLAFNVSGRPYDSFASHYHPYAPIYSNRERLLIYVSDVALFSVT

YSLYRVATLKGLVWLLCVYGVPLLIVNGFLVTITYLQHTHFALPHYDSSEWDWLKGALAT

MDRDYGILNKVFHHITDTHVAHHLFSTMPHYHAMEATNAIKPILGEYYQFDDTPFYKALW

REARECLYVEPDEGTSEKGVYWYRNKY

>GmSLD.5

MEPPTSYISTDELRKHNTRHDAWISIHGKIYDVSSWLHRHPGGPLPLLTLAGTDATDAFL

AFHPPNTASLLLPAFSTSHRLSDHTVSAASSDYRKLFSDLSALNLFNRKGHTTSILLSLI

LTLFPLSVCGVLFSDSTFVHVLSAALIGFLWIQSGWIGHDSGHYNVMLSRRLNRAIQILS

GNILAGISIGWWKWNHNAHHIACNSLDYDPDLQHLPFFVVSSRFFNSLTSRFYDRKLNFD

SFARFLVSYQHWTFYPVMCFARVNLFAQSFFLLLSKRKVENRGSELLGLLAFWVWYPLLV

SFLPNWWERLLFVFVSFSVTGIQHVQFCLNHFSSEVYLGPPSGGDWFEKQTKGTLGVDCS

PWMDWFHGGLQFQVEHHLFPRLPRCHLRKIAPLVKDLCKKHNLPYNCVSFWKANVLTIQT

LRNAALQARDGSNPVPKNLVWEAVNTHG

>GmFAD2.2

MGGGGRSSATLKHQNSIKNHSKKKRVPHAKPPFTLSQLKKAISPHCFHRSTFRSFSYVLY

DLTIASCLFYAAVNYIPTLPHENLSLLAWPLYWFIQGSILTGVWVIAHECGHHAFSDHQW

LDDLVGLILHSLLLVPYFSWKYSHRRHHSNTGSLERDEVFVPKTKSSMGWYSKYLNNSPG

RVLTLAITLTLGWPLYLAFNVSGRSYERFACHYDPYGPIYSNRERLQIYVSDAGILAVCF

GLYKAVLAKGLVWVVCVYGVPLLVVNGFLVLITFLQHTHPAVPHYDSSEWDWLRGALATV

DRDYGILNKVLHNITDTHVAHHLFSTMPHYHAMEATKAIKPILGEYYHFDETPIYKAMWR

EAKECMYVEPDKGSNGKGVYWYNNKL

>GmFAB2.2

MLPLLHLTHSSMALRLNPIPTQTFSLPQMPSLRSPRFRMASTLRSGSKEVENIKKPFTPP

REVHVQVTHSMPPQKIEIFKSLEDWADQNILTHLKPVEKCWQPQDFLPDPSSDGFEEQVK

ELRERAKEIPDDYFVVLVGDMITEEALPTYQTMLNTLDGVRDETGASLTSWAIWTRAWTA

EENRHGDLLNKYLYLSGRVDMKQIEKTIQYLIGSGMDPRTENSPYLGFIYTSFQERATFI

SHGNTARLAKEHGDIKLAQICGMIASDEKRHETAYTKIVEKLFEVDPDGTVMAFADMMRK

KIAMPAHLMYDGRDDNLFDNYSAVAQRIGVYTAKDYADILEFLVGRWKVEQLTGLSGEGR

KAQEYVCGLPPRIRRLEERAQARGKESSTLKFSWIHDREVLL

>GmADS.1

MALLTSQPKKPILHRFTTLHRPLPRLDRTVLGLGSFSHNTLSIKKFNFNPKQTKISLKNN

PFTYNLAVTAKANANAAFGEGLEAGEVTLKHRKILLSEVEVKRQRRVFFGRKWNSLDVGT

AGIVLAMHLLCLFAPFHFNWPAFRVAVALYIVTGLFGITLSFHRNLSHRSFKLPKWLEYF

FAYCGVLALQGNPIDWVSTHRYHHQFCDSERDPHSPTEGFWFSHMSWLFDTSSVIERCGE

ANNVGDLEKQSFYRFLRSSYLVHPFALGALLYAAGGFPFLVWGMGVRIVWVYHITWFVNS

ACHVWGNQAWNTRELSRNNWWVALLAFGEGWHNNHHAFEYSARHGLEWWQLDMTWYVVRF

LEAIGLATDVKLPTESHKQKMALNSDPIDT

>GmFAD7.1

MATWVLSECSLKPLAPVIPRPRTGAVLSSTSKVGFLETNKVLEGSKFQPLRCNLRERNWG

LKVSVPLRIASIEEEEQKSVDVINGSNGVEHEKLPEFDPGAPPPFNLADIRAAIPKHCWV

KDPLKSMSYVVRDVIAVFGLASAAAYLNNWLVWPLYWAAQGTMFWALFVLGHDCGHGSFS

NNPKLNSVVGHLLHSSILVPYHGWRISHRTHHQHHGHVENDESWHPLPEKLFRSLDTVTR

MLRFTAPFPLLAYPVYLWGRSPGKTGSHFDPSSDLFVPNERKDVITSTACWAAMLGLLVG

LGFVMGPIQLLKLYGVPYVIFVMWLDLVTYLHHHGHEGKLPWYRGKEWSYLRGGLTTLDR

DYGLINNIHHDIGTHVIHHLFPQIPHYHLVEATEAAKPVFGKYYREPKKSSPLPFHLIGE

LIRSFKTDHFVSDKGDVVYYQTDSEIIGSSKSE

>GmDES.2

MGRGEDREGVAVAGGFFWSYTDEPHATRRRQILSKYPQIKQLFGPDHSAFFKISGVVLLQ

LGTGALLHDAGWLKIFLVAYFFGSFLNHNLFLAIHELSHNLAFSTPVYNRWLGIFANLPI

GVPMSVTFQKYHLEHHRFQGVDGIDMDVPSLTEVRLVKNIIAKTIWVFLQLFFYALRPLF

LKPKPPGIWEFINFSVQIALDVSMVYFFGWKALAYLILSTFVGGGMHPMAGHFISEHYVF

NPDQETYSYYGPLNYLTWHVGYHNEHHDFPRIPGNKLHKVKEIAPEFYDNLASYRSWSQI

IYMYIMDRTVGPFSRMKRKSSKAE

>GmFAB2.3

MQIQTLPLASPAPRITRHHFPRPPKCAVTATPPPLKERRNHSMPPEKIEIFKSLEGWASQ

RVLPLLKPVEQCWQPQKFLPDSTLPFDEFIEAVRSLRHRTKELSDEYFLVLVGDMVTEEA

LPTYQTIMSGLDGVGDKCGSNPSPWAVWTRAWSAEENRHGDLLRTYLYLSGRVDMKMIER

TIHNLIAAGMDPGFENNPYLGFVYTSFQERATFVSHGNTARLAKEGGDPVLACICGTIAA

DEKRHENAYSRIVEKLLEVDPTGAMLAIGTTMEKKITMPAHLMYDGEDPRLFEHYSTVAQ

RMGVYTAKDYADILEFLIGRWRLEKLEGLTAEGKRAQDFVCGLAPRIRKLQERADERARK

MKPHGVKFSWIFNKELHL

>GmDES.1

MGRGEDREGVAAAGGFFWSYTDEPHATRRRLILSKYPQIKQLFGPDHSAFFKISGVVLLQ

LGTGALLHDAGWLKIFLVAYFFGSFLNQNLFLAIHELSHNLAFSTPVYNRWLGIFANLPV

GVPMSVTFQKYHLEHHRFQGVDGIDMDVPSLTEVRLVKNMIAKTIWVFLQLFFYAFRPLF

LKPKPPGIWEFINFSVQIALDVSMVYFFGWKALAYLILSSFLGGGMHPMAAHFISEHYVF

NPDQETYSYYGPLNYLAWHVGYHNEHHDFPRIPGNKLYKVKEIAPEFYDSLASYRSWSQI

IYMYIMDRTVGPFSRMKRKSSKAE

>GmFAB2.4

MAPEKIEIFKSLEGWASQQVLPLLKPVEQIRNFFIGIFYLIENRPLAIDSKQAINHIKRK

YELENMEIVNKEKEKVRYDFLVFVECNFHQHDFVPNSSLPFKEFTDEVRALRERTMELPD

EYFVVLVGDMITEEALPTYQTTMNNLDGVRDEYGACQSPWAVWTRAWSVEENRHGDLLKT

YMYLSGRVDMERVEKTIHYLIASGWDVGMENNPYLGFVYTSFQERATFVAHGNTARLAKE

GGDPVLARICDTRTQVDPTGAMLAIGKMMQKKIIMPAHLMYDGDDPRLFEHYSAVAQRIG

VYTANDYANILDFLVGRWRLEKLESLTAEGKRAQDYVCELPPRIRKLQERADERARKMKP

NSFKFNWIFNKELLL

>GmSLD.2

MEVVEKEKKKYITSEELKGHNKEGDLWISIQGKVYNVSDWVKEHPGGDVPISNLAGQDVT

DAFIAYHPGTAWSHLDKFFTGYHLSDFKVSEVSKDYRKLASEFSKLGLFDTKGHVTSCTL

ASVAVMFLIVMYGVLRCTSVWAHLGSGMLLGLLWMQSAYVGHDSGHYVVMTSNGFNKVAQ

ILSGNCLTGISIAWWKWTHNAHHIACNSLDHDPDLQHMPVFAVSSRFFNSITSHFYGRKL

EFDFIARFLICYQHFTFYPVMCVARVNLYLQTILLLFSRRKVQDRALNIMGILVFWTWFP

LLVSFLPNWPERVMFVLASFAVCSIQHIQFCLNHFAANVYVGLPSGNDWFEKQTSGTLDI

SCSSSMDWFFGGLQFQLEHHLFPRLPRCQLRNISPLVSDLCKKHNLPYRSLSFWEANQWT

IRTLRTAALQARDLTNPAPKNLLWEAVNTHG

>GmFAD6

MACTLADSLLLFKGSYQKPVLRRDIAARYSPGIFSLNSNGLIQKRFRRQRNFVTRNKVTV

IHAVAIPVQPAPVESAEYRKQLAEDYGFRQVGEPLPDDVTLKDVINSLPKEVFEIDDVKA

WKSVLISVTSYALGLFMISKAPWYLLPLAWVWTGTAITGFFVIGHDCAHRSFSSNKLVED

IVGTLAFMPLIYPYEPWRFKHDRHHAKTNMLREDTAWHPVWKDEFESTPLLRKAIIYGYG

PFRCWMSIAHWLMWHFDLKKFRPSEVPRVKISLACVFAFIAIGWPLIIYKTGIMGWIKFW

LMPWLGYHFWMSTFTMVHHTAPHIPFKYSEEWNAAQAQLNGTVHCDYPKWIEILCHDINV

HIPHHISPRIPSYNLRAAHKSLQENWGQYLNEASWNWRLMKTIMTVCHVYDKEQNYVAFD

ELAPEDSRPITFLKETMPDYA

>GmFAB2.1

MALRLNPIPTQTFSLPQMASLRSPRFRMASTLRSGSKEVENIKKPFTPPREVHVQVTHSM

PPQKIEIFKSLEDWAEQNILTHLKPVEKCWQPQDFLPDPSSDGFEEQVKELRERAKELPD

DYFVVLVGDMITEEALPTYQTMLNTLDGVRDETGASLTSWAIWTRAWTAEENRHGDLLNK

YLYLSGRVDMKQIEKTIQYLIGSGMDPRTENSPYLGFIYTSFQERATFISHGNTARLAKE

HGDIKLAQICGMIASDEKRHETAYTKIVEKLFEVDPDGTVMAFADMMRKKIAMPAHLMYD

GRDDNLFDSYSSVAQRIGVYTAKDYADILEFLVGRWKVEQLTGLSGEGRKAQEYICGLPP

RIRRLEERAQARVKESSTLKFSWIHDREVLL

>GmFAD3.1

MVKDTKPLAYAANNGYQQKGSSFDFDPSAPPPFKIAEIRASIPKHCWVKNPWRSLSYVLR

DVLVIAALVAAAIHFDNWLLWLIYCPIQGTMFWALFVLGHDCGHGSFSDSPLLNSLVGHI

LHSSILVPYHGWRISHRTHHQNHGHIEKDESWVPLTEKIYKNLDSMTRLIRFTVPFPLFV

YPIYLFSRSPGKEGSHFNPYSNLFPPSERKGIAISTLCWATMFSLLIYLSFITSPLLVLK

LYGIPYWIFVMWLDFVTYLHHHGHHQKLPWYRGKEWSYLRGGLTTVDRDYGWINNIHHDI

GTHVIHHLFPQIPHYHLVEATQAAKPVLGDYYREPERSAPLPFHLIKYLIQSMRQDHFVS

DTGDVVYYQTDSLLLHSQRD

>GmFAD2.4

MGAGGRTAVPPANRKSEADPLKRVPFEKPQFSLSQIKKAIPPHCFQRSVLRSFSYVVYDL

TIAFCLYYVATHYFHLLPGPLSFVAWPIYWAVQGCILTGVWVIAHECGHHAFSDYQLLDD

IVGLILHSALLVPYFSWKYSHRRHHSNTGSLERDEVFVPKQKSSIMWYSKYLNNPPGRVL

TLAVTLTLGWPLYLAFNVSGRPYDRFACHYDPYGPIYSDRERLQIYISDAGVLAVCYGLF

CLAMAKGLAWVVCVYGVPLLVVNGFLVLITFLQHTHPALPHYTSSEWDWLRGALATVDRD

YGILNKVFHNITDTHVAHHLFSTMPHYHAMEATKAIKPILGEYYRFDGTPFVKAMWREAR

ECIYVEPDQSTQSKGVFWYNNKL

>GmFAD8.2

MATWVLSECGLRPLPPVFPRSTRPISCQKPSKSRFLSTNKGVPDLNLQARGLTCCSFRDR

KWELGVSAPLKFATNEGEEEERTNGANNGVGEEVSEFDPSAPPPFKLADIRAAIPKHCWV

KDPWKSMSYVVRDVIVVFGLAAAAAYLNNWVVWPLYWAAQGTMFWALFVLGHDCGHGSFS

NNPKLNSVAGHLLHSSILVPYHGWRISHRTHHQNHGHVENDESWHPLPEKIFKSLDNVTR

ILRFTLPFPLLAYPIYLWSRSPGKTGSHFNPDSDLFVPSERKDVITSTICWTAMAALLVG

LGFVMGPVQLLKLYGIPYAIFVMWLDLVTYLHHHGHEDKLPWYRGEEWSYLRGGLTTIDR

DYGWINNIHHDIGTHVIHHLFPQIPHYHLIEATEAAKPVLGQYYREPKKSSPLPIYLIGE

LLRSMKKDHFVSDSGDIVYYQTDPTLSSSSTSQ

>GmFAD2.1

MGAGGRTDVPPANRKSEVDPLKRVPFEKPPFSLSQIKKVIPPHCFQRSVFRSFSYVVYDL

TIAFCLYYVATHYFHLLPSPLSFLAWPIYWAVQGCILTGVWVIAHECGHHAFSDYQLLDD

IVGLVLHSGLLVPYFSWKYSHRRHHSNTGSLERDEVFVPKQKSCIKWYSKYLNNPPGRVL

TLAVTLTLGWPLYLALNVSGRPYDRFACHYDPYGPIYSDRERLQIYISDAGVLAVCYGLF

RLAMAKGLAWVVCVYGVPLLVVNGFLVLITFLQHTHPALPHYTSSEWDWLRGALATVDRD

YGILNKVFHNITDTHVAHHLFSTMPHYHAMEATKAIKPILGEYYRFDETPFVKAMWREAR

ECIYVEPDQSTESKGVFWYNNKL

>GmFAD2.5

MGLAKETIMGGGGRVAKVEIQQKKPLSRVPNTKPPFTVGQLKKAIPPHCFQRSLLTSLSY

VVYDLSLAFIFYIATTYFHLLPHPFSLIAWPIYWVLQGCILTGVWVIAHECGHHAFSKYP

WVDDVMGLTVHSALLVPYFSWKISHRRHHSNTGSLDRDEVFVPKPKSKVAWYTKYLNNPL

GRAASLLITLTIGWPLYLAFNVSGRPYDGFASHYHPYAPIYSNRERLLIYVSDVALFSVT

YLLYRVATMKGLVWLLCVYGVPLLIVNGFLVTITYLQHTHYALPHYDSSEWDWLRGALAT

MDRDYGILNKVFHHITDTHVAHHLFSTMPHYHATEATNAMKPILGEYYRFDDTPFYKALW

REARECLYVEPDEGTSEKGVYWYRNKY

>GmFAD3.2

MVQAQPLQHVGNGAGKEDLAYNFDPSAPPPFKIADIRAAIPKHCWEKNTLRSLSYVLRDV

LIVSALVAAAIGFNSWLFWPPYWSAQGTMFWALFVLGHDCGHGSFSNSPMLNSIVGHILH

SSILVPYHGWRISHRTHHQNHGHVEKDESWVPLSEKVYKNLDNMTRMMRFTLPFPIFAYP

FYLWSRSPGKEGSHFNPYSNLFSPGERRDVITSTLCWGIMLSLLLYLSLTLDPLFMFKLY

GVPYLIFVVWLDFVTYLHHHGYKQKLPWYRGQEWTYLRGGLTTVDRDYGWINNIHHDIGT

HVIHHLFPQIPHYHLVEATKAAKAVLGKYYREPQKSGPLPLHLIKYLLHSISQDHFVSDY

GDIVYYQTDSQFHKDSWTKSN

>GmFAB2.5

MPPEKKEIFKSLEGWASEWVLPLLKPVEQCWQPQNFLPDPSLPHEEFSHQVKELRERTKE

LPDEYFVVLVGDMVTEDALPTYQTMINNLDGVKDDSGTSPSPWAVWTRAWTAEENRHGDL

LRTYLYLSGRVDMAKVEKTVHYLISAGMDPGTDNNPYLGFVYTSFQERATFVAHGNTARL

AKEGGDPVLARLCGTIAADEKRHENAYSRIVEKLLEVDPTGAMVAIGNMMEKKITMPAHL

MYDGDDPRLFEHYSAVAQRIGVYTANDYADILEFLVERWRLEKLEGLMAEGKRAQDFVCG

LAPRIRRLQERADERARKMKKHHGVKFSWIFNKELLL

>GmFAD3.3

MVKDTKPLAYAANNGYQKEAFDPSAPPPFKIAEIRVAIPKHCWVKNPWRSLSYVLRDVLV

IAALMAAASHFNNWLLWLIYWPIQGTMFWALFVLGHDCGHGSFSDSPFLNSLVGHILHSS

ILVPYHGWRISHRTHHQNHGHIEKDESWVPLTEKIYKNLDNMTRLVRFTVPFPLFVYPIY

LFSRSPGKEGSHFNPYSNLFPPSERKGIAISTLCWVTMFSMLIYLSFITSPVLLLKLYGI

PYWIFVMWLDFVTYLHHHGHHQKLPWYRGKEWSYLRGGLTTVDRDYGWINNIHHDIGTHV

IHHLFPQIPHYHLVEATQAAKSVLGEYYREPERSAPLPFHLIKYLIQSMRQDHFVSDTGD

VVYYQTDSLHLHSHRD

>GmSLD.4

MDPPTSYISSDELRKHNTRHDAWISIHGKVYDVSSWLHRHPGGPLPLLSLAGTDATDAFL

ALHPPNSASLLLPAFATPHLLSDHSVSPASSDYRKLFSDLSSLNLFNRKGHTTSILLSLI

LVLFPLSLCGVLLSDSTCVHVLSGALVGFLWIQSGWIGHDSGHYNVMLNPRLNRAIQILS

GNVLSGISIGWWKWNHNAHHIACNSLDFDPDLQHLPFFVVSSSFFNSLTSRFYERKMNFD

SFSRFLVSYQHWTFYPVMCFARINLFAQSFFLLLSKRKVENRWSELLGLLAFWVWYPLLV

SFLPNWWERVLFVFVSFSVTGIQHVQFCLNHFSSEVYLGPPSGGDWFEKQTKGTLGVDCS

PWMDWFHGGLQFQVEHHLFPRLPRCHLRKIAPLVKDLCKKHNLPYNCVSFWKANVLTIQT

LRNAALQARDGSKPVPKNLVWEAVNTHG

>GmADS.2

MALLTSQPKNPILHRFTTLHCPLPRLHRTVLGLGSFSHNSALSTKKLNFNPTQTKISLQN

NPFTYNLAVTAKANANAAFGEGFKEEGASRHRKILLSEVEVKRERRVFFGRKWNSLDFGT

AGIVLAMHLLCVFAPFHFNWPAFWVAVALYIVTGLFGITLSFHRNLSHRSFKLPKWLEYF

FAYCGVLALQGNPIDWVSTHRYHHQFCDSERDPHSPTEGFWFSHMSWLFDTNSVLERCGE

ANNVGDLEKQSFYRFLRSTYLAHPFALGALLYAAGGFPFLVWGMGVRIVWVYHITWFVNS

ACHVWGNQAWNTRDLSRNNWWVALLAFGEGWHNNHHAFEYSARHGLEWWQIDMTWYVVRF

LQAIGLATDVKLPTESQKQKMAFNSDSIAT

>GmSLD.3

MEGENKKYITSEELKEHNKPGDLWISIQGKVYNVSDWAKDHPGGEVPLLNLAGQDVTDAF

VAYHPGSAWKYLDPFFTGYHLRDFKVSEVSKDYRKLVSEFVKMGLFEKKEHVTLYTLSSV

AVMFSIVVYGVIGCSSVWAHLGAALLLGLLWMQSTYVGHDSGHYEVMSSPGYNKLAQILC

GNCMTGISIAWWKWTHNAHHISCNSLDYDPDLQHIPVFAVSTRFFNSIKSCFYGRKLVFD

SLSRFLISYQHFTFYPVLCFARVNLYLQTLLLLFSRRKVPDRAFNIMGILVFWVWFPLLI

SSLPNWGERVMFVLASFAVCSIQHLQFCLNHFSADVYEGPPNGNDWFEKQTGGTLDISCS

SWMDWFFGGLQFQLEHHLFPRLPRAQLRKISPLVIDLCKKHNLPYRSLTFVEANLWTLKT

LRTAALQARNLSNPSSQNLLWEAFNTHG

>GmFAD4

MYSLAQHKYTPNFHHQVCKNHPPRHPSRVHCSTTTTTTTTSRSKSNAKSLVIETRLVPVP

PMPTVVTTEIHRPMNNDPSLQSTWSHRAWVAAGCSTLVISLGESIKGAIDLNMWVEPIVA

GWVGYILADLGSGVYHWAIDNYGDGSTPIVGAQIEAFQGHHKWPWTITRRQFANNLHALA

RAVTLAVLPVVLLCHDPIVEGFVVVCSGCIMFSQQFHAWSHGTKSRLPPLVVALQEAGVL

VSRWQHAAHHRAPYNNNYCIVSGVWNEFLDKHKVFEAMEMVLYFKTGVRPRSWSEPAPEW

VEEIETPSQIQIQTQ

>GmSLD.1

MEVVEKEKKYITSEELKGHNKEGDLWISIQGKVYNVSDWVKEHPGGDVPISNLAGQDVTD

AFIAYHPGTAWSHLEKFFTGYHLSDFKVSEVSKDYRKLASEFSKLGLFDTKGHVTSCTLA

SVAVMFLIVLYGVLRCTSVWAHLGSGMLLGLLWMQSAYVGHDSGHYVVMTTNGFNKVAQI

LSGNCLTGISIAWWKWTHNAHHIACNSLDHDPDLQHMPVFAVSSRFFNSITSHFYGRKLE

FDFIARFLICYQHFTFYPVMCVARVNLYLQTILLLFSRRKVQDRALNIMGILVFWTWFPL

LVSCLPNWPERVMFVLASFAVCSIQHIQFCLNHFAANVYVGPPSGNDWFEKQTSGTLDIS

CASSMDWFFGGLQFQLEHHLFPRLPRCQLRKISPLVSDLCKKHNLPYRSLSFWEANQWTI

RTLRTAALQARDLTNPAPKNLLWEAVNTHG

>GmFAD8.1

MATWVLSECGLRSLPPVFPRPTRPISCQKPSKFRFLSINKGVADLNLQPRGFTCYNFRER

KWESGVSAPLKVATTEGEEEEGINGANGVVEEVPEFDPSAPPPFKLADIRASIPKHCWVK

DPWKSMSYVVRDVIVVFGLAVAAAYLNNWVVWPLYWAAQGTMFWALFVLGHDCGHGSFSN

NPKLNSVAGHLLHSSILVPYHGWRISHRTHHQNHGHVENDESWHPLPEKIFKSLDNVTRI

LRFTLPFPLLAYPIYLWSRSPGKTGSHFNPDSDLFVPSERKDVITSTVCWTAMAALLVGL

GFVMGPVQLLKLYGIPYVIFVMWLDLVTYLHHHGHEDKLPWYRGEEWSYLRGGLTTIDRD

YGWINNIHHDIGTHVIHHLFPQIPHYHLIEATEAAKPVLGLYYREPKKSSPLPIYLIGEL

LRSMKKDHFVSNTGDIVYYQTDPTLSSSSTSQ

>GmFAD3.4

MVQAQPLQHVGNGAGKEDQAYFDPSAPPPFKIANIRAAIPKHCWEKNTLRSLSYVLRDVL

VVTALVAAAIGFNSWFFWPLYWPAQGTMFWALFVLGHDCGHGSFSNSPLLNSIVGHILHS

SILVPYHGWRISHRTHHQNHGHVEKDESWVPLTEKVYKNLDNMTRMMRFTLPFPIFAYPF

YLWSRSPGKEGSHFNPYSNLFSPGERRDVLTSTLCWGIMLSVLLYLSLTMGPLFMLKLYG

VPYLIFVMWLDFVTYLHHHGYKQKLPWYRGQEWSYLRGGLTTVDRDYGWINNIHHDIGTH

VIHHLFPQIPHYHLIEATKAAKAVLGKYYREPQKSGPLPLHLIKYLLHSISQDHFVSDSG

DIVYYQTDSQLHKDSWTQSN

>GmSLD.6

MKEFEEEGGVNPIIIGWRRRTRLLVAVGFDLVRNHEIQYRLWRVEFKELKEHNKSGDLWI

SIQGKVYNVSDWAKDHPGGEVPLLNLAGQDVTDAFIAYHPGSAWKYLDPFFTGYHLRDFK

VSEVSKDYRKLVSEFAKVGLFEKKEHVTLYTLSSVAVMFSIVVYGVIGCNSVWAHLGAAL

LLGLLWMQSTYVGHDSGHYEVMSSPGYNKLAQILCGNCMTGISIAWWKWTHNAHHISCNS

LDYDPDLQHIPVFAVSTRFFNSIKSCFYGRKLVFDSLSRFLISYQHWTFYPVLCFARVNL

YLQTLLLLFSRRKVPDRAFNIMGILVFWIWFPLLISSLPNWGERVMFVLASFAVCSIQHL

QFCLNHFAADVYEGPPNGNDWFEKQTGGTLDISCSTWMDWFFGGLQFQLEHHLFPRLPRA

QLRKISPLVIDLCKKHNLPYRSLTFVEANLWTLKTLRTAALQARNLSNPSSQNLLWEAFN

THG

>GmFAD7.2

MATWILSECGLKPLAPVIPRPRTGAALSSTSRVEFLDTNKVVAGPKFQPLRCNLRERNWG

LKVSAPLRVASIEEEQKSVDLTNGTNGVEHEKLPEFDPGAPPPFNLADIRAAIPKHCWVK

DPWRSMSYVVRDVIAVFGLAAAAAYLNNWLVWPLYWAAQGTMFWALFVLGHDCGHGSFSN

NSKLNSVVGHLLHSSILVPYHGWRISHRTHHQHHGHAENDESWHPLPEKLFRSLDTVTRM

LRFTAPFPLLAFPVYLFSRSPGKTGSHFDPSSDLFVPNERKDVITSTACWAAMLGLLVGL

GFVMGPIQLLKLYGVPYVIFVMWLDLVTYLHHHGHEDKLPWYRGKEWSYLRGGLTTLDRD

YGWINNIHHDIGTHVIHHLFPQIPHYHLVEATEAAKPVFGKYYREPKKSAAPLPFHLIGE

IIRSFKTDHFVSDTGDVVYYQTDSKINGSSKLE

>OsFAB2.1

MQVVGTVRVSGCGAVVAPSRRQCRVSAAVLTAAETATATRRRVTHSMPPEKAEVFRSLEG

WARSSLLPLLKPVEECWQPTDFLPDSSSEMFEHQVHELRARAAGLPDEYFVVLVGDMITE

EALPTYQTMINTLDGVRDETGASACPWAVWTRTWTAEENRHGDILGKYMYLSGRVDMRMV

EKTVQYLIGSGMDPGTENNPYLGFVYTSFQERATAVSHGNTARLARAHGDDVLARTCGTI

AADEKRHETAYGRIVEQLLRLDPDGAMLAIADMMHKRITMPAHLMHDGRDMNLFDHFAAV

AQRLNVYTARDYADIVEFLVKRWKLETLETGLSGEGRRARDFVCGLAKRMRRAAERAEDR

AKKDEQRKVKFSWIYDREVIV*

>OsFAB2.2

MASRMALRPNDVTLRLTPPLAAAARRNRRAAAGGVRVYAVASGAVSTKVENKKPFAPPRE

VHVQVTHSMPPQKIEIFKSLDDWARDNILSHLKPVEKCWQPQDFLPDPASDGFHDEVKEL

RERAKEIPDDYFVCLVGDMITEEALPTYQTMLNTLDGVRDETGASPTAWAVWTRAWTAEE

NRHGDLLNKYLYLTGRVDMRQIEKTIQYLIGSGMDPRTENNPYLGFIYTSFQERATFISH

GNTARHAKDFGDLKLAQICGIIASDEKRHETAYTKIVEKLFEIDPDGTVLAFADMMKKKI

SMPAHLMFDGEDDKLFEHFSMVAQRLGVYTAKDYADILEFLVSRWKISDLTGLSSEGNKA

QDYLCTLAARIRRLDERAQSRAKKAGTLPFSWVYGREVQL*

>OsFAB2.3

MASTAGVGGIGNPTPRGKKPFAPWREVPPQVTHTLPPEKKEVFDSLEGWAADTILPYLKP

VEESWQPQDHLPDPRSPSFGDEVAALRERAAGLPDDHLVCLVGDMVTEEALPTYQTMLNT

MDGGVRDETGAGGSAWAVWTRAWAAEENRHGDLMNKYLYLTGRVDMRQVEKTIQYLIGSG

MVVGRALLTGVLKFLEADPRTENDPYMGFIYTTFQERATSISHGNTARHAGRHGDAALAR

VCGTVAADEKRHEAAYAAIVAKLFEVDPDYTVRAFARMMRRKVAMPARLMYDGADDRLFA

RFAAVAQRLGVYTAADYAGIIEFLVARWGVPGLAAGLSGEGRRAQDFVCSLGPRFRRMEE

RAQEAAKRAPPAAAAPFSWIHGRQVQL*

>OsDES1

MGAAAGDGREEEGVMATDFFWSYTDEPHATRRREILAKHPQIKELFGPDPLAFLKIAAVV

SLQLWTATLLRDASWVKILTVAYFFGSFLNHNLFLAIHELSHNLAFTTPSYNRWLGIFAN

LPIGVPMSITFQKYHLEHHRFQGVDGIDMDIPSQAEAHAVKNTLSKSVWVVFQLFFYALR

PLFLKPKPPGLWEFTNLIIQIALDASMVYFFGWKSLAYLILSTFVGGGMHPMAGHFISEH

YVFNPDQETYSYYGPLNLMTWHVGYHNEHHDFPRIPGTRLYKVREIAPEYYNNLKSYKSW

SQVIYMYIMDQTVGPFSRMKRKAPKKDS*

>OsFAD2.1

MLLNSCIRNLLLIGLVARYFCSCGVSVLLHYNIAQIAPSDPTTILQSILTFSSPAIVIVV

VSSKMGAGGRMTEKEREEQQKLLGRAGNGAAVQRSPTDKPPFTLGQIKKAIPPHCFQRSV

IKSFSYVVHDLVIVAALLYFALVMIPVLPSGMEFAAWPLYWIAQGCVLTGVWVIAHECGH

HAFSDYSVLDDIVGLVLHSSLLVPYFSWKYSHRRHHSNTGSLERDEVFVPKQKSAMAWYT

PYVYHNPIGRLVHIFVQLTLGWPLYLAFNVSGRPYPRFACHFDPYGPIYNDRERVQIFIS

DVGVVSAGLALFKLSSAFGFWWVVRVYGVPLLIVNAWLVLITYLQHTHPALPHYDSSEWD

WLRGALATVDRDYGILNKVFHNITDTHVAHHLFSTMPHYHAMEATKAIRPILGEYYQFDP

TPVAKATWREAKECIYVEPEDNKGVFWYNNKF*

>OsFAD7

MARLVLSECCGLTPLRLRGRGAIALPAPPSLAAGPRRPVSAAAAGGAIHREWALRVSAPT

RLTSVVEEDNRGEEVVEEEARGSLAAAEAAAGEVGGDGDGFDPGAPPPFGLAEIRAAIPK

HCWVKDPWRSMSYVLRDVVVVLGLAAAAARVDSWLVWPLYWAAQGTMFWALFVLGHDCGH

GSFSSNAKLNSVVGHILHSSILVPYHGWRISHRTHHQNHGHVEKDESWQPLSERLYNSLD

YMTKKLRFTMPFPMLAFPLYLFARSPGKKGSHFNPSSDLFQPNEKKDVITSTASWLAMVG

ILAGLTFVMGPLKMLKLYAVPYVIFVMWLDFVTYLHHHGHEDKLPWYRGKEWSYLRGGLT

TLDRDYGWINNVHHDIGTHVIHHLFPQIPHYHLVEATEAAKPVLGKYYKEPEKSAPLPFH

LLGVLAKSLNSDHYVSDTGDVVYYQTDLKTSSSAQSSD*

>OsFAB2.4

MASSGLAVAATASSAWLCCPNHHIHTSSSRSRKHLLLHGLYGSAPARTRGRRPPVWTAAA

ATAAAPADTAASARREQVEIARSLNAWVEENMLPLLTPVDSAWQPHDFLPCSAAGGGEAL

AAFTEGVAELRAGAAGVPDEVLVCLVGNMVTEEALPTYQSMGNRAEGLADGTGVSPLPWA

RWLRGWTAEENRHGDLLNRYLYLSGRVDMRQVEATVHRLLRNGMEMLAPASPYHGLIYGA

FQERATFISHGHTARLAGQHGDRALAKICGVIAADERRHEAGYTMASGRLFELDPDGMAR

ALADVMRGKVTMPGQLMSDGRDGDGEHSLFARFSAVAERAGVYTARDYGDLVEHFVRRWR

VAELAAGLSGEGRRAQEYLCGLAPKIRRMEELAHRRAARIEPAMARFSWIFDRPVMLG*

>OsFAB2.5

MAFAASHTASPSSCGGVAQRRSNGMSPVVAMASTINRVKTAKKPYTPPREVHLQVKHSLP

PQKREIFDSLQPWAKENLLNLLKPVEKSWQPQDFLPDPSSDGFYDEVKELRERAKEIPDD

YFVCLVGDMVTEEALPTYQTMLNTLDGVRDETGASPTTWAVWTRAWTAEENRHGDLLNKY

MYLTGRVDMKQIEKTIQYLIGSGMDPGTENNPYLGFLYTSFQERATFISHGNTARHAKEY

GDLKLAQICGTIAADEKRHETAYTKIVEKLFEIDPDYTVLAFADMMRKKISMPAHLMYDG

KDDNLFEHFSAVAQRLGVYTARDYADILEFLVQRWKVADLTGLSGEGRRAQDFVCTLAPR

IRRLDERAQARAKQAPVIPFSWVYDRKVQL*

>OsFAD2.2

MGTSSRPTTVKEGKKLEAPRRAGSHAAVKRSPVDKPPFTLGDIRKAIPPHCFHRSVIKSF

SYLLHDLAIAAGLLYFALVVIPALPGVLRLVAWPFYWAAQGCFLFGVWIIAHECGHHAFS

GHALLDDTLGLVLHSWLLAPYFSWKYTHQRHHSNTSSQERDEVFVPRFKSDLPWYSPYVY

KYNNPVARLLLLVVQLTVGWPMYLVFNTWGRQYPRFASHFDPSGPIYKGRERVFIAISDI

GMLAVSLALYRLAEGYGFWWVVRVYGVPLLVVNAWLVVVTYLHHTHRAIPHYDSSEWDWL

RGALATVDRDYSFLNRVFHNITDTHVVHHLFPTIPHYHAVEATKAIRPILGEYYQFDPTP

IVKAIWREAKECIYIQSEDHKGVFWYSNKF*

>OsFAD2.3

MTRRGCTCSLRASQPRTSPLSSGPTAGPSRTATILCLSSSGVQAADAAELTILCCFLSKM

LLPTTYVQEQLRTVINRNTGTSGRRTTTVVEGKKQELLLRRSGSSAAMQRSPVDKPPFTL

GDIKKAIPPHCFHRSVIKSFSYLLHDLAIAAGLLYFALVGIPALPSILRLVAWPLYWAAQ

GSVLTGVWVIGHECGHHAFSDYLLLDNLVGLVLHSALLTPFFSWKYSHRRHHANTGSMEK

DEVYVAKKKSALPWYTPYVFGNPVGRLVYIALQLTLAWPLYLAFNLSGQPYPRLVTCHYD

PYSPLFSDQERVQVLVSDAAILAVLLALHRLTAAYGLWWVVRVYGVPVMIVGALFVLITY

LHHTHRALPHYDSSEWEWLRGSLATVDRDYGVLNRVLHNVTDTHVLHHLFPSMPHYHAME

ATRAARPVLGEYYKFDRTPIIEATWREAKECMYVEPRERDGIYWYNNKF*

>OsFAD8

MARLLLSGVAPLPLLPCRRRAIAFALPLGNVRLRLRVAAPTSRVATVEEDDNENNAPPPP

CEDFDPGAAPPFGLADIRAAIPKHCWVKDPWRSMGYVLRDVVVVFALAAAAARLHSCLAW

PLYWAAQGTMFWALFVLGHDCGHGSFSNNSRLNSVMGHILHSSILVPYHGWRISHRTHHQ

NHGHVDKDESWHPLPERLYRSLNRATRMLRFSIPFPMLAYPFYLWSRSPGKSGSHFHPSS

DLFQPNERNDVLTSTACWVAMAALLAGLTFLMGPLLMLNLYFVPYWIFVMWLDFVTYLHH

HGHNDKLPWYRGKEWSYLRGGLTTVDRDYGWINNIHHDIGTHVIHHLFPQIPHYHLIEAT

EAAKGVMGKYYREPDKSGPFPLHLFGALSRSLKRDHYVSDTGDVVYYQTDPAN*

>OsFAD4

MYAMTPRCHLPPPCRAASTTPATSTALSTPPPSRAGPDELRSTWAHRAWTLAGSAAVLSS

LSTSATLAAADADANGAAAAFAAPLAAALAAYSLADLATGVYHWLVDNYGDADTPVLGPQ

IAAFQGHHRHPSTITRREPCNNLHALARAVALALPPAGAALAAAGAPASAHAFAAVFAAC

VVLSQQFHAWAHGNPRRLPPGVGAMQRAGVLVSRAQHGAHHRAPYDNNYCIVSGMWNATL

DRHRVFEAMEMVVFLRTGVRPRSWDEPDAAWTEDYDDTAAVAGGDTSLDTQ*

>OsFAB2.6

MAATATMAMPLANRLRCKPNTNSSSPSRTLFGRRVTMISSSRWMCRGSAVSGSAIMSAAA

DVAAAVRREEDEEMRSYLSPEKLEVLTQMEPWVEEHVLPLLKPVEAAWQPSDLLPDPAVL

GGEGFHAACAELRERAAGVPDLLLVCLVANMVTEEALPTYQSSLNRVRAVGDLTGADATA

WARWIRGWSAEENRHGDVLNRYMYLSGRFDMAEVERAVHRLIRSGMAVDPPCSPYHAFVY

TAFQERATAVAHGNTARLVGARGHGDAALARVCGTVAADEKRHEAAYTRIVSRLLEADPD

AGVRAVARMLRRGVAMPTSPISDGRRDDLYACVVSLAEQAGTYTVSDYCSIVEHLVREWR

VEELAAGLSGEGRRARDYVCELPQKIRRMKEKAHERAVKAQKKPISIPINWIFDRHVSVM

LP*

>OsFAB2.7

MAASATTSTLAVTMFGYPNRNCHLKPPATATLRFWRSAAAAAVATSRREAEAEEADEVRR

CLAPARLEVLEQMEPWVEAHVLPLLKPAEEAWQPADLVPDAAALGADGFHAACVELRGRA

AGVPDAHLVCLVGNMVTEEALPTYQSMANRFESARDVTGADATAWARWIRGWSAEENRHG

DVLNRYMYLSGRLDMRQVERTVHRLIGSGMAMHAPASPYHGFIYVAFQERATAISHGNTA

RNVRAHGDDALARICGAIASDEKRHEAAYTRVVERLLEADPDTTVRALAYMMRRRITMPA

ALMDDGRDADLFAHYAAAAQQAGTYTASDYRGILEHLIRRWRVAELEAGLSGEGRRARDY

VCALPQKIRRMEEKAHDRAAQMRKRPTAIPFSWIFDKPVDLMLP*

>OsFAD6

MATASGVSTPLQLPSTRRVGGCCSRPGSPAPGKNAFPRRAAGGAPAGTFFLKRDSVYKGQ

SCHQFLPLKQSGRLQAAVLPVTPPLLDDEEKRKQMCEDYGFKQIGEQLPDNVTLKDVMDS

LPKEVFEIDDLKSWTSVLISVTSYALGIFLISKAPWYLLPLAWAWTGTAVTGFFVIGHDC

AHKSFSRNKLVEDIVGTLAFLPLIYPYEPWRFKHDRHHAKTNMLIEDTAWQPVFSKEFRT

NSLLRKAMIFAYGPIRPWMSIAHWLIWHFDLKKFRPNELPRVKISLASVLAFMAIGWPLI

ILKSGIAGWFKFWFMPWMVYHFWMSTFTMVHHTAPHIPFKTSEEWNAAQAQLNGTVHCDY

PRWIEILCHDINVHVPHHISPRIPSYNLRAAYDSIKQNWGKYINEASWNWRLMKTILTKC

HVYDKDRYYVPFDEVAPEESQPIKFLKKVMPDYA*

>OsSLD1

MPPAIPTATMPASPKEAQSRAGAGVRMISSEELRAHASRDDLWISISGDVYDVTAWVPHH

PGGDIPLLTLAGQDATDAFAAYHPPSARPLLGRFLVGRLEDYTVSPASADFRRLLAQLSS

AGLFERVGPTPKVQVAGMLLLLCAALYCVLACASAWAHLLAGGLIGFIWIQSGWMGHDSG

HHRITGHAALDRLLQVLSGNCLTGLSIAWWKCNHNTHHIACNSLDHDPDLQHMPLFAVSS

KLFGLWSYFYQRTLVFDAASKFLISYQHWTFYPVMCFARINLLIQSAVFLLSSRKVPQRG

LEIAGVAAFWVWYPMVVSCLPNWWERVAFVVASFVITGIQHVQFCLNHFSSEVYVGPPKG

NDWFEKQTAGTLDIQCSPWMDWFHGGLQFQIEHHLFPRLPRCHLRKVSPFVRDLCKKHGL

PYAAASFWQANVLTWKTLRAAALQARKATSGAAPKNLVWEAVNTHG*

>OsFAD3.1

MLNNVVGHLLHSFILVPYHGWRISHRTHHQNHGHIERDESWHPITEKLYWQLETRTKKLR

FTLPFTLLAFPWYRSPGKTGSHFLPSSDLFSPKEKSDVIVSTTCWCIMISLLVALACVFG

PVPVLMLYGVPYLVFVMWLDLVTYLHHHGHNDLPWYRGEEWSYLRGGLTTVDRDYGWINN

IHHDIGTHVIHHLFPQIPHYHLVEATKAARPVLGRYYREPEKSGPLPLHLFGVLLRSLRV

DHFVSDVGDVVYYQTDHSLNGTDWAEDAKHK*

>OsFAD3.2

MAASATQEADCKASEDARLFFDAAKPPPFRIGDVRAAIPAHCWRKTPLRSLSYVARDLLI

VAALFAAAATRIDVSVAWAAWPLYWAAQGTMFWALFVLGHDCGHGSFSDSAMLNNVVGHL

LHSFILVPYHGWRISHRTHHQNHGHIEKDESWHPITEKLYRKLETRTKKLRFTLPFPLLA

FPVYLWYRSPGKTGSHFLPSSDLFSPKEKSDVIVSTTCWCIMISLLVALACVFGSVPVLM

LYGVPYLVFVMWLDLVTYLHHHGHNDLPWYRGEEWSYLRGGLTTVDRDYGWINNIHHDIG

THVIHHLFPQIPHYHLVEATKAARPVLGRYYREPEKSGPLPLHLFGVLLRSLRVDHFVSD

VGDVVYYQTDHSLNGTDWAEDAKHK*

>AtADS1

MSLSASEKEENNKKMAADKAEMGRKKRAMWERKWKRLDIVKAFASLFVHFLCLLAPFNFT

WPALRVALIVYTVGGLGITVSYHRNLAHRSFKVPKWLEYFFAYCGLLAIQGDPIDWVSTH

RYHHQFTDSDRDPHSPNEGFWFSHLLWLFDTGYLVEKCGRRTNVEDLKRQWYYKFLQRTV

LYHILTFGFLLYYFGGLSFLTWGMGIGVAMEHHVTCLINSLCHVWGSRTWKTNDTSRNVW

WLSVFSFGESWHNNHHAFESSARQGLEWWQIDISWYIVRFLEIIGLATDVKLPSESQRRR

MAMVR*

>AtADS8

MGDTTKDDGSSQSKAVRGEKRAFFFRKWTRIDIARASAVGAVHLLCLLAPFNYKWEALRF

GVILAIVTSLSITFSYHRNLTHKSFKLPKWLEYPFAYSALFALQGHPIDWVSTHRFHHQF

TDSDRDPHSPIEGFWFSHVFWIFDTSYIREKCGGRDNVMDLKQQWFYRFLRNTIGLHILT

FWTLVYLWGGLPYLTCGVGVGGTIGYNGTWLINSACHIWGSRAWNTKDTSRNIWWLGPFT

MGESWHNNHHAFEASARHGLEWYQVDLTWYLICFFQALGLATDVKLPTDAQKRKLAFAR*

>AtADS7

MSETTKDDGSSQKKSVRKEKRAYVLRKWTQFDVGRASTVGTVHLLCLLAPFNYKWEAFRF

GIILAILTNLCITFSYHRNLTHRSFKLPKWLEYPFAYSALLALQGDPLDWVSIHRFHHQF

TDSDRDPHSPIEGFWFSHVLWIFDTDYIREKCGRRNNVMDLKQQWFYRFLKKTLVLHILA

FWTLIYLWGGLPYLTWTVGFGGVIGYHGTWLVNSACHICGSQAWQTNDTSRNVWWLALLT

MGESWHNNHHAFETSARHGLEWYQLDITWYLIWFFQALGLATNVKLPTDAQKRKMAIRR*

>AtADS9

MGDKNKDDSSSQSKAVRKEKRAFLFRKWTRVDVMRVSAVGAVHLLCLLAPFNYTWEAFRF

AAMVGISTNLSITFSYHRNLTHRSFKLPKWLEYPFAYSALFALQGHPIDWVSTHRFHHQF

TDSDRDPHSPIEGFWFSHVFWIFDTSYIREKCGGRDNVMDLKQQWFYRFLQNTIGLHILT

FWILVYLWGGLPYLTWSVGVGGAIGYHATWLINSACHIWGSRAWNTKDTSRNIWWLGPFT

MGESWHNNHHAFEASARHGLEWYQVDLTWYLIWFFQVLGLATDVKLPTDAQKRKMSLAR*

>AtADS4

MCDPTRDDGSSRSRVVSTMQKRAYFQRQWPLVDVVRASVVVIVHFLCLLAPFNFKWEALR

FGLVLFALTTLSITFSFHRNLSHRSFKIPKWLEYPWAYSAVFALQGDPMDWVSIHRFHHQ

FTDSDRDPHSPKEGLLFSHILWIFDTQYIKYKCGGRDNVLDLKKQWFYKFLRRTIAVHIL

MFWTILYLYGGLPYLTCGGGVGIFIGYHVTWLVNSACHIWGSRSWNTKDTSRNVWWLSLF

TMGESWHNNHHAFESSARQGLEWWQIDITWYLIRLFEVLGIATDVKLPSELQKQKMALVR

*

>AtADS6

MCDPIREDGSNKRGAVSKEKRPYIHREWSWADIIRALTVINVHFLCLLAPFNYKWEALRF

GFVLYALTSLSITFSYHRNLAHRSFKLPKWLEYPLAYFAVFALQGDPLDWVSIHRFHHQF

TDSDRDPHSPIEGFWFSHVWWICDTRYIKYKCGGRNNVMDLKQQWFYWFLRMTIGFHVLM

FWTVLYLYGGLPYLTCGGGVGGVIGYHVTWLVNSACHIWGSRSWKTKDTSRNVWWLSLFT

MGESWHNNHHAFESSARQGLEWWQIDITWYLIRLFEVLGLATDVKLPSEIQKQKLALTR*

>AtFAB2.1

MLAHKSLLSFTTQWATLMPSPSTFLASRPRGPAKISAVAAPVRPALKHQNKIHTMPPEKM

EIFKSLDGWAKDQILPLLKPVDQCWQPASFLPDPALPFSEFTDQVRELRERTASLPDEYF

VVLVGDMITEDALPTYQTMINTLDGVRDETGASESAWASWTRAWTAEENRHGDLLRTYLY

LSGRVDMLMVERTVQHLIGSGMDPGTENNPYLGFVYTSFQERATFVSHGNTARLAKSAGD

PVLARICGTIAADEKRHENAYVRIVEKLLEIDPNGAVSAVADMMRKKITMPAHLMTDGRD

PMLFEHFSAVAQRLEVYTADDYADILEFLVGRWRLEKLEGLTGEGQRAQEFVCGLAQRIR

RLQERADERAKKLKKTHEVCFSWIFDKQISV*

>AtFAD4.3

MAVSFQTKNPLRPITNIPRSYGPTRVRVTCSVTTTNPQLNHENLVVEKRLVNPPLSKNND

PTLQSTWTHRLWVAAGSTTIFASFAKSIIGGFGSHLWLQPALACYAGYVFADLGSGVYHW

AIDNYGGASTPIVGAQLEASQGHHKYPWTITKRQFANNSYTIARAITFIVLPLNLAINNP

LFHSFVSTFAFCILLSQQFHAWAHGTKSKLPPLVMALQDMGLLVSRKDHPGHHQAPYNSN

YCVVSGAWNKVLDESNLFKALEMALFFQFGVRPNSWNEPNSDWTEETETNFFTKI*

>AtFAD4.4

MATSLQTKYTLNPITNNIPRSHRPSFLRVTSTTNSQPNHEMKLVVEQRLVNPPLSNDPTL

QSTWTHRLWVAAGCTTVFVSFSKSIIGAFGSHLWLEPSLAGFAGYILADLGSGVYHWATD

NYGDESTPLVGIHIEDSQDHHKCPWTITKRQFANNLHFMARGTTLIVLPLDLAFDDHVVH

GFVSMFAFCVLFCQLFHAWAHGTKSKLPPLVVGLQDIGLLVSRIHHMNHHRAPYNNNYCV

VSGVWNKVLDESNVFKAMEMVLYIQLGVRPRSWTEPNYE*

>AtFAD3

MVVAMDQRTNVNGDPGAGDRKKEERFDPSAQPPFKIGDIRAAIPKHCWVKSPLRSMSYVV

RDIIAVAALAIAAVYVDSWFLWPLYWAAQGTLFWAIFVLGHDCGHGSFSDIPLLNSVVGH

ILHSFILVPYHGWRISHRTHHQNHGHVENDESWVPLPERVYKKLPHSTRMLRYTVPLPML

AYPLYLCYRSPGKEGSHFNPYSSLFAPSERKLIATSTTCWSIMFVSLIALSFVFGPLAVL

KVYGVPYIIFVMWLDAVTYLHHHGHDEKLPWYRGKEWSYLRGGLTTIDRDYGIFNNIHHD

IGTHVIHHLFPQIPHYHLVDATKAAKHVLGRYYREPKTSGAIPIHLVESLVASIKKDHYV

SDTGDIVFYETDPDLYVYASDKSKIN*

>AtADS2

MSVTSTVEENHQKNPSTPAAVEEKKKRRWVFWDRRWRRLDYVKFSASFTVHSLALLAPFY

FTWSALWVTFLFYTIGGLGITVSYHRNLAHRSFKVPKWLEYLLAYCALLAIQGDPIDWVS

THRYHHQFTDSERDPHSPKEGFWFSHLLWIYDSAYLVSKCGRRANVEDLKRQWFYRFLQK

TVLFHILGLGFFLFYLGGMSFVTWGMGVGAALEVHVTCLINSLCHIWGTRTWKTNDTSRN

VWWLSVFSFGESWHNNHHAFESSARQGLEWWQIDISWYIVRFFEIIGLATDVKVPTEAQR

RRMAIVR*

>AtFAB2.2

MALKFNPLVASQPYKFPSSTRPPTPSFRSPKFLCLASSSPALSSGPKEVESLKKPFTPPR

EVHVQVLHSMPPQKIEIFKSMENWAEENLLIHLKDVEKSWQPQDFLPDPASDGFEDQVRE

LRERARELPDDYFVVLVGDMITEEALPTYQTMLNTLDGVRDETGASPTSWAIWTRAWTAE

ENRHGDLLNKYLYLSGRVDMRQIEKTIQYLIGSGMDPRTENNPYLGFIYTSFQERATFIS

HGNTARQAKEHGDIKLAQICGTIAADEKRHETAYTKIVEKLFEIDPDGTVMAFADMMRKK

ISMPAHLMYDGRNDNLFDNFSSVAQRLGVYTAKDYADILEFLVGRWKIQDLTGLSGEGNK

AQDYLCGLAPRIKRLDERAQARAKKGPKIPFSWIHDREVQL*

>AtSLD2

MADQTKKRYVTSEDLKKHNKPGDLWISIQGKVYDVSDWVKSHPGGEAAILNLAGQDVTDA

FIAYHPGTAWHHLEKLHNGYHVRDHHVSDVSRDYRRLAAEFSKRGLFDKKGHVTLYTLTC

VGVMLAAVLYGVLACTSIWAHLISAVLLGLLWIQSAYVGHDSGHYTVTSTKPCNKLIQLL

SGNCLTGISIAWWKWTHNAHHIACNSLDHDPDLQHIPIFAVSTKFFNSMTSRFYGRKLTF

DPLARFLISYQHWTFYPVMCVGRINLFIQTFLLLFSKRHVPDRALNIAGILVFWTWFPLL

VSFLPNWQERFIFVFVSFAVTAIQHVQFCLNHFAADVYTGPPNGNDWFEKQTAGTLDISC

RSFMDWFFGGLQFQLEHHLFPRLPRCHLRTVSPVVKELCKKHNLPYRSLSWWEANVWTIR

TLKNAAIQARDATNPVLKNLLWEAVNTHG*

>AtFAB2.6

MKMALLLNSTITVAMKQNPLVAVSFPRTTCLGSSFSPPRLLRVSCVATNPSKTSEETDKK

KFRPIKEVPNQVTHTITQEKLEIFKSMENWAQENLLSYLKPVEASWQPQDFLPETNDEDR

FYEQVKELRDRTKEIPDDYFVVLVGDMITEEALPTYQTTLNTLDGVKDETGGSLTPWAVW

VRAWTAEENRHGDLLNKYLYLSGRVDMRHVEKTIQYLIGSGMDSKFENNPYNGFIYTSFQ

ERATFISHGNTAKLATTYGDTTLAKICGTIAADEKRHETAYTRIVEKLFEIDPDGTVQAL

ASMMRKRITMPAHLMHDGRDDDLFDHYAAVAQRIGVYTATDYAGILEFLLRRWEVEKLGM

GLSGEGRRAQDYLCTLPQRIRRLEERANDRVKLASKSKPSVSFSWIYGREVEL*

>AtFAB2.7

MALLLNSTMTVAMKQNPATAVSFMQTTCLGSSFSPPRHLQVSCVATNPSKTFRPIKEVSN

QVTHTITQEKLEIFKSMENWAQENLLSYLKPVETSWQPQDFLPETKDEDRFYEQVKELRD

RTKEIPDDYFVVLVGDMITEEALPTYQTVMNTLDGAKDETGVSLTPWAVWLRAWTAEENR

HGDLLNKYLYLSGRVDTRHVEKTIQYLIGSGMDTKYENNPYNGYIYTSFQERATFISHAN

TAKLATTYGDTTLAKICGTIAADEKRHEMAYTRIVEKLFEIDPDGTVQALASMMRKRITM

PAQLMHDGRDDNLFDHYAAVAQRIGVYTATDYAGILEFLLRRWEVEKLGMGLSGEGRRAQ

DYLCTLPQRIRRLEERADDRVKRASKSKPSVSFSWIYGREVEL*

>AtFAB2.3

MAMAMDRIVFSPSSYVYRPCQARGSRSSRVSMASTIRSATTEVTNGRKLYIPPREVHVQV

KHSMPPQKLEIFKSLEGWADETLLTYLKPVEKSWQPTDFLPEPESEGFYDQVKELRERCK

ELPDDYFVVLVGDMITEEALPTYQTMLNTLDGVRDETGASPTPWAIWTRAWTAEENRHGD

LLNKYLYLSGRVDMRQIEKTIQYLIGSGMDPKTENNPYLGFIYTSFQERATFISHGNTAR

LAKDRGDLKLAQICGTIAADERRHETAYTKIVEKLFEIDPDGTILGLADMMKKKISMPAH

LMYDGQDDNLFEHFSTVAQRLGVYTAKDYADILEFLVERWNVETLTDLSSEGHRAQDFVC

GLPARIRKIEERAQGRAKEAAKNIPFSWIFGRNIRA*

>AtFAD7

MANLVLSECGIRPLPRIYTTPRSNFLSNNNKFRPSLSSSSYKTSSSPLSFGLNSRDGFTR

NWALNVSTPLTTPIFEESPLEEDNKQRFDPGAPPPFNLADIRAAIPKHCWVKNPWKSLSY

VVRDVAIVFALAAGAAYLNNWIVWPLYWLAQGTMFWALFVLGHDCGHGSFSNDPKLNSVV

GHLLHSSILVPYHGWRISHRTHHQNHGHVENDESWHPMSEKIYNTLDKPTRFFRFTLPLV

MLAYPFYLWARSPGKKGSHYHPDSDLFLPKERKDVLTSTACWTAMAALLVCLNFTIGPIQ

MLKLYGIPYWINVMWLDFVTYLHHHGHEDKLPWYRGKEWSYLRGGLTTLDRDYGLINNIH

HDIGTHVIHHLFPQIPHYHLVEATEAAKPVLGKYYREPDKSGPLPLHLLEILAKSIKEDH

YVSDEGEVVYYKADPNLYGEVKVRAD*

>AtFAD2

MGAGGRMPVPTSSKKSETDTTKRVPCEKPPFSVGDLKKAIPPHCFKRSIPRSFSYLISDI

IIASCFYYVATNYFSLLPQPLSYLAWPLYWACQGCVLTGIWVIAHECGHHAFSDYQWLDD

TVGLIFHSFLLVPYFSWKYSHRRHHSNTGSLERDEVFVPKQKSAIKWYGKYLNNPLGRIM

MLTVQFVLGWPLYLAFNVSGRPYDGFACHFFPNAPIYNDRERLQIYLSDAGILAVCFGLY

RYAAAQGMASMICLYGVPLLIVNAFLVLITYLQHTHPSLPHYDSSEWDWLRGALATVDRD

YGILNKVFHNITDTHVAHHLFSTMPHYNAMEATKAIKPILGDYYQFDGTPWYVAMYREAK

ECIYVEPDREGDKKGVYWYNNKL*

>AtADS3

MASLLTKPKPVFLCSPSLSPRTLNTATPSLNFTRISFTHHQKLAPFKPPSLVVAFSEKGL

KRDVTTAAAATEGDYRRIMLSDVLVKKKEKVVWWEREWKAMDFGAVAVVLSMHLLSLLAP

FQFNWRAVSVAFGLYIVTGLLGITLSFHRNLSHKAFKLPKWLEYLFAYCGAQALQGNPID

WVSTHRYHHQFCDSDRDPHSPLDGFWFSHMNWMFDTNTITQRCGEPNNVGDLEKQPFYRF

LRTTYILHPLALAVALYAMGGFPFIVWGMGVRIVWVYHITWLVNSACHVWGKQAWNTGDL

SKNNWWVAALAFGEGWHNNHHAFEFSARHGLEWWQLDMTWYVVKFLQAIGLATDVKLPSE

AQKQRMAFTSD*

>AtADS5

MMSLSTTLKPLSHFSPFVKRHNPKTNNTLFTLDTHNFTNSFWSKRGGSVSHRKHTVVAVY

EAPDHVESSWRRLLSEVVVVRTKRSFWERSWTSWDVSKLVIFVGTHLLSLLAPFYFSWEA

FWVFPWLVFINGICITLSYHRNLSHRSFDLPKWLEYLFAYGGVLAFQGDPIEWVSNHRYH

HKHCETQRDPHSPTQGFWFSHMAWIFDTSSILENCGGEENVDDLVRQPFYRFLQRTVLLH

MMAYSFLFYFCGGMPLLVWGIGITIAVRLHLTFLVNSVCHIWGTRAWNTSDFSKNNWWVA

ILTLGEGWHNNHHAFEFSARHGLEWWQLDITWCLIRFLEAIGLATNVKLPTETQMKGKAL

V*

>AtSLD1

MAEETEKKYITNEDLKKHNKSGDLWIAIQGKVYNVSDWIKTHPGGDTVILNLVGQDVTDA

FIAFHPGTAWHHLDHLFTGYHIRDFQVSEVSRDYRRMAAEFRKLGLFENKGHVTLYTLAF

VAAMFLGVLYGVLACTSVFAHQIAAALLGLLWIQSAYIGHDSGHYVIMSNKSYNRFAQLL

SGNCLTGISIAWWKWTHNAHHLACNSLDYDPDLQHIPVFAVSTKFFSSLTSRFYDRKLTF

DPVARFLVSYQHFTYYPVMCFGRINLFIQTFLLLFSKREVPDRALNFAGILVFWTWFPLL

VSCLPNWPERFFFVFTSFTVTALQHIQFTLNHFAADVYVGPPTGSDWFEKQAAGTIDISC

RSYMDWFFGGLQFQLEHHLFPRLPRCHLRKVSPVVQELCKKHNLPYRSMSWFEANVLTIN

TLKTAAYQARDVANPVVKNLVWEALNTHG*

>AtDES1

MGKGGREKISSNEEEREGVMATDFFWSYTDEPHASRRRQILSCYPQIRQLFGPDPWAFLK

ITLVVILQLSTAAILHNSGWLKILSIAYFFGSFLNHNLFLAIHELSHNLAFSTPVYNRCL

GIFANLPIGVPMSVTFQKYHLEHHRFQGVDGIDMDVPTYTEAHLVTNIFAKTIWVFLQLF

FYALRPIFIKPKPPGYWEFINFLIQIVLDVSVVLFFGWRSFAYLILSTFVGGGMHPMAGH

FISEHYVFNPNQETYSYYGPLNLLTWSVGYHNEHHDFPRIPGNKLHLVKEIAGEYYEGLE

SYKSWSQVIYMYIMDTTVGPYSRMKRKLSKSD*

>AtFAD4.1

MAVSLPTKYPLRPITNIPKSHRPSLLRVRVTCSVTTTKPQPNREKLLVEQRTVNLPLSND

QSLQSTKPRPNREKLVVEQRLASPPLSNDPTLKSTWTHRLWVAAGCTTLFVSLAKSVIGG

FDSHLCLEPALAGYAGYILADLGSGVYHWAIDNYGDESTPVVGTQIEAFQGHHKWPWTIT

RRQFANNLHALAQVITFTVLPLDLAFNDPVFHGFVCTFAFCILFSQQFHAWAHGTKSKLP

PLVVALQDMGLLVSRRQHAEHHRAPYNNNYCIVSGAWNNVLDESKVFEALEMVFYFQLGV

RPRSWSEPNSDWIEETEISNNQA*

>AtFAD6

MASRIADSLFAFTGPQQCLPRVPKLAASSARVSPGVYAVKPIDLLLKGRTHRSRRCVAPV

KRRIGCIKAVAAPVAPPSADSAEDREQLAESYGFRQIGEDLPENVTLKDIMDTLPKEVFE

IDDLKALKSVLISVTSYTLGLFMIAKSPWYLLPLAWAWTGTAITGFFVIGHDCAHKSFSK

NKLVEDIVGTLAFLPLVYPYEPWRFKHDRHHAKTNMLVHDTAWQPVPPEEFESSPVMRKA

IIFGYGPIRPWLSIAHWVNWHFNLKKFRASEVNRVKISLACVFAFMAVGWPLIVYKVGIL

GWVKFWLMPWLGYHFWMSTFTMVHHTAPHIPFKPADEWNAAQAQLNGTVHCDYPSWIEIL

CHDINVHIPHHISPRIPSYNLRAAHESIQENWGKYTNLATWNWRLMKTIMTVCHVYDKEE

NYIPFDRLAPEESQPITFLKKAMPNYTA*

>AtFAD8

MASSVLSECGFRPLPRFYPKHTTSFASNPKPTFKFNPPLKPPSSLLNSRYGFYSKTRNWA

LNVATPLTTLQSPSEEDTERFDPGAPPPFNLADIRAAIPKHCWVKNPWMSMSYVVRDVAI

VFGLAAVAAYFNNWLLWPLYWFAQGTMFWALFVLGHDCGHGSFSNDPRLNSVAGHLLHSS

ILVPYHGWRISHRTHHQNHGHVENDESWHPLPESIYKNLEKTTQMFRFTLPFPMLAYPFY

LWNRSPGKQGSHYHPDSDLFLPKEKKDVLTSTACWTAMAALLVCLNFVMGPIQMLKLYGI

PYWIFVMWLDFVTYLHHHGHEDKLPWYRGKEWSYLRGGLTTLDRDYGWINNIHHDIGTHV

IHHLFPQIPHYHLVEATEAAKPVLGKYYREPKNSGPLPLHLLGSLIKSMKQDHFVSDTGD

VVYYEADPKLNGQRT*

>AtFAB2.5

MSMALLLTSPAMKQKPAVITSPRRGSSPSRRLRVSCVTTNPARKKNETCNHFRPIKEVNN

QLTHTIPQEKLEIFKSMENWAEQKLLPYLKPVEDSWQPQDFLPAPENDDEFYDRVKEIRE

RTKEIPDDYFVVLVGDMITEEALPTYQTTLNTLDGVKDETGGSLSPWAVWIRAWTAEENR

HGDLLNKYLYLTGRVDMRHVEKTIQYLIGSGMDSKFENNPYNGFIYTSFQERATFISHGN

TARLATTYGDVTLAKICGTIAADEKRHETAYTKIVEKLFEIDPDGSVQALASMMKKRITM

PAHLMHDGRDNDLFDHYAAVAQRIGVYTAADYAGILEFLLRRWKVESLGLGLSGEGRRAQ

EYLCTLPQRIKRLEERANDRVKLVSKPSVSFSWVFGRDVKL*

>AtFAB2.4

MVMAMDRIALFSSSSSVYHHGSSHSHGSKSSRVFTIRSDSTAVGRKLYIPPREVHLQVKY

SMPPQKLEIFKSLEGWANDNLLAYLKPVEKSWQPTDFLPEPESEGFYDQVKELRERCKEL

SDDYLIVLVGDMITEEALPTYQTMINTLDGVRDETGASPTPWAVWTRAWTAEENRHGDLL

NKYLYLSGRVDMRQIEKTIQYLIGSGMDPKTENNPYLGFIYTSFQERATFISHGNTARLA

KDLGDLTLGKICGTIAADERRHEHAYTKIVEKLFEIDPDTTVVGFADMMRKKISMPAHLM

YDGRDDNLFDHFSSVAQRLGVYTAKDYADILQHLVERWNVEKLSDLSSEGNRAQDYLCGL

PARIRKLEERAQGRTKEAAKNIPFSWIFGREVRA*
